# Unsupervised learning predicts human perception and misperception of gloss

**DOI:** 10.1038/s41562-021-01097-6

**Published:** 2021-05-06

**Authors:** Katherine R. Storrs, Barton L. Anderson, Roland W. Fleming

**Affiliations:** 1 Department of Experimental Psychology, Justus Liebig University Giessen, Giessen, Germany; 2 School of Psychology, University of Sydney, Sydney, Australia; 3 Centre for Mind, Brain and Behaviour (CMBB), University of Marburg and Justus Liebig University Giessen, Giessen, Germany

**Keywords:** Human behaviour, Object vision, Sensory processing

## Abstract

Reflectance, lighting and geometry combine in complex ways to create images. How do we disentangle these to perceive individual properties, such as surface glossiness? We suggest that brains disentangle properties by learning to model statistical structure in proximal images. To test this hypothesis, we trained unsupervised generative neural networks on renderings of glossy surfaces and compared their representations with human gloss judgements. The networks spontaneously cluster images according to distal properties such as reflectance and illumination, despite receiving no explicit information about these properties. Intriguingly, the resulting representations also predict the specific patterns of ‘successes’ and ‘errors’ in human perception. Linearly decoding specular reflectance from the model’s internal code predicts human gloss perception better than ground truth, supervised networks or control models, and it predicts, on an image-by-image basis, illusions of gloss perception caused by interactions between material, shape and lighting. Unsupervised learning may underlie many perceptual dimensions in vision and beyond.

## Abstract (web summary)

Storrs et al. train unsupervised generative neural networks on glossy surfaces and show how gloss perception in humans may emerge in an unsupervised fashion from learning to model statistical structure.

## Main

A photograph of a glass of water might consist of large bright patches, sparkling dots and low-contrast blurs—yet we immediately see these as reflections on the water’s surface, bubbles within and smudges on the glass. Identifying the distal physical causes of proximal image features is widely considered to be the central challenge of vision^1–7^. Yet how we infer the outside world from ambiguous sensory data remains mysterious.

Somehow, the visual system infers the combination of distal scene variables that most plausibly explains the proximal sensory input^4,8–11^. But a major unsolved question is how the candidate explanations of proximal inputs came to be known in the first place. Our visual systems did not—and do not—have access to ground truth information about the number or kinds of distal sources of image structure that operate in the world. Any knowledge about the world must have been acquired from exposure to proximal stimuli over evolutionary and/or developmental timescales^12–14^.

Here we explore the intriguing possibility that visual systems might be able to discover the operation of distal scene variables by learning statistical regularities in proximal images rather than by learning an explicit mapping between proximal cues and known distal causes. Specifically, we show that by learning to efficiently compress and spatially predict images of surfaces, an unsupervised generative deep neural network (DNN) not only spontaneously clusters inputs by distal factors such as material and illumination but also strikingly reproduces many characteristic ‘misperceptions’ of human observers.

Two general principles motivate our approach. The first is that variability in proximal sensory stimuli is caused by variations in a lower-dimensional set of environmental factors (such as shape, reflectance and illumination). This implies that the variation between images can be captured in a more compact or simple way when represented in terms of its underlying causes rather than, say, in terms of pixels^15–17^. A machine learning model encouraged to discover a compact representation of images might therefore converge on the finite sources that generate image variability. But identifying and disentangling these distal sources is possible only if a second principle holds: different distal sources must generate statistically distinguishable effects in the proximal input^18^. This seems intuitively true—for example, changes in illumination generate different kinds or patterns of variability in images than changes in surface material do. On the basis of these two principles, we reasoned that it should be possible for a sufficiently powerful statistical learning model to discover the existence of distal variables without a priori knowledge of either the number or kinds of distal variables that exist in the world, solely on the basis of the variability they generate in images.

The idea that our perceptual systems exploit statistical regularities to derive information about the world has a long and venerable history in both psychology and neuroscience^19–21^. For example, neural response properties in early visual cortex are well predicted by models trained to generate sparse codes for natural image patches^19,21–24^. Unfortunately, such ‘efficient coding’ approaches have not yet scaled beyond the initial encoding of images. One of the main motivations for the present work was to determine whether such ideas could provide leverage into mid-level scene understanding—that is, inferring the distal physical causes of sense data.

Even if different distal factors have different statistical effects on images, and a sufficiently powerful unsupervised neural network is able to learn these effects, its success in disentangling different factors is unlikely to be perfect—that is, the network would sometimes misattribute the distal causes responsible for the data. However, we regard such misattributions as a potential strength. There are well-documented examples where the human visual system systematically misattributes image structure to the wrong distal source—failures of ‘perceptual constancy’^1,5,25–33^. We were interested in whether the pattern of successful and unsuccessful attributions made by human observers would also be exhibited by networks that failed to fully disentangle distal scene variables. The goal was not to understand visual processes as an estimation of ground truth but rather to understand why our visual systems extract what they do about the world in the absence of access to ground truth knowledge.

One of the most striking patterns of successes and failures in estimating distal scene variables occurs in the perception of surface gloss^3,5,31,34–37^. Gloss perception is a paradigmatic case of a perceptual judgement where multiple physical effects must be separated. The pattern of specular reflections can change dramatically as a function of a surface’s three-dimensional (3D) shape, illumination and the observer’s viewpoint. Indeed, psychophysical evidence has shown that the perception of gloss in human observers depends not only on specular reflectance, as expected^32,38–44^, but also on lighting and shape^33–37,45^. We were interested in whether the specific pattern of these complex interactions could be a consequence of the visual system having learned to approximately disentangle distal sources from their effects on image structure.

Our work exploits DNN methods that have emerged for learning sophisticated models of image structure in the form of latent variables, which summarize how images differ from one another^46–50^. DNNs achieve complex transformations by passing input data through layers of units that apply weighted summations and nonlinearities, roughly mimicking the operations of biological neurons^51–55^. During training, connections between units are iteratively adjusted to improve the network’s performance on some learning objective.

In supervised learning, networks are directly told what label to output for every input image in a training dataset, from which they learn to subsequently output appropriate labels for new test images. Supervised DNNs have revolutionized computer vision, achieving near-human object and face recognition^56–59^, and are the best extant models of late ventral stream function^51–53,60–63^. However, unlike humans, they are often fragile to tiny image perturbations^64,65^ and over-rely on local texture^66,67^. As models of biological gloss perception, it is unclear from where the necessary training labels could come.

**Fig. 1:**
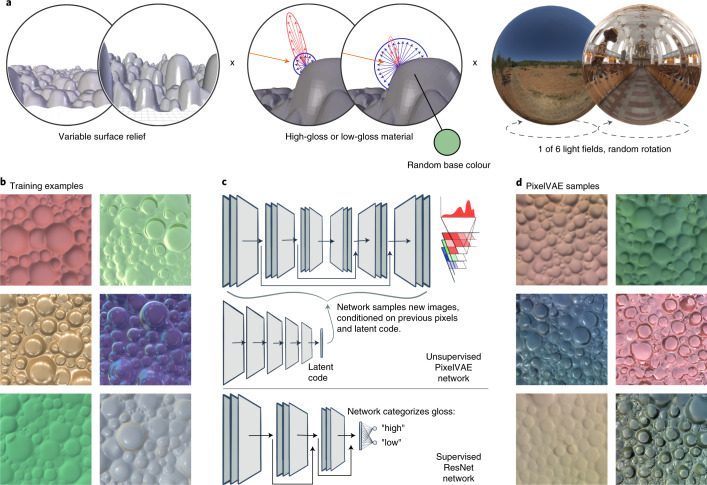
An unsupervised neural network learns to generate plausible novel images from a simulated visual world. **a**, The simulated physical world consisted of a bumpy surface viewed from above. The scene varied in depth of surface relief (left), surface base colour and whether the surface material had high or low gloss (middle), and illumination environment (right). **b**, Six examples from a training set of 10,000 images rendered from the simulated world by randomly varying the world factors. **c**, The dataset was used to train an unsupervised PixelVAE network (top). The network learns a probability distribution capturing image structure in the training dataset, which can be sampled from one pixel at a time to create novel images with similar structures. A substream of the network learns a highly compressed 10D latent code capturing whole-image attributes, which is used to condition the sampling of pixels. The same dataset was used to train a supervised ResNet network (bottom), which output a classification of ‘high gloss’ or ‘low gloss’ for each input image. The penultimate layer of the supervised network is a 10D fully connected layer, creating a representation of equivalent dimensionality for comparison with the unsupervised model’s latent code. **d**, Six example images created by sampling pixels from a trained PixelVAE network, conditioning on different latent code values to achieve diverse surface appearances. These images are not reconstructions of any specific images from the training set; they are completely novel samples from the probability distribution that the network has learned.

In unsupervised learning, training objectives encourage networks to learn statistical regularities in the training data without being given any explicit labels. For example, autoencoder networks are trained to compress training images into compact descriptions and then reconstruct them as accurately as possible^50,68,69^. Here, we use a variant known as a PixelVAE^47,48^, which learns to both summarize and spatially predict images, in two connected processing streams (Fig. 1c). It is a generative model that can create completely novel images with high-order statistical structure similar to the training data—in our case, images of glossy and matte surfaces.

Our main finding is that the representation of gloss learned by an unsupervised PixelVAE network closely matches the pattern of successes and failures in perceived gloss shown by human observers. The unsupervised models better match human data than do a range of supervised networks and simpler comparison models, suggesting that the learning process by which different distal sources are disentangled may play a fundamental role in shaping our visual experience of the world. This finding provides a potential answer to the conundrum of how we learn to see without explicit training.

## Results

To test whether an unsupervised DNN can learn human-like gloss perception, we rendered 10,000 images from a virtual world consisting of frontal views of bumpy surfaces with either high (‘gloss’) or low (‘matte’) specular reflectance. Using renderings grants tight control over the statistics of the training environment, allowing us to guarantee that reflectance could not be trivially decoded from raw images and that physical factors varied independently of one another. Each image had a different random configuration of bumps, depth of surface relief and colour, and was illuminated by one of six natural light fields (Fig. 1a,b). We then trained ten different instances of a PixelVAE^47,48^ network with different initial random weights on this dataset to ensure that the results were robust to representational differences between training instances of the same architecture^70^. The network culminates in a probability distribution over pixel values (Fig. 1c). Its training objective is to adjust the shape of this distribution in order to increase the likelihood of the training images under it, leading to a model of the structure and variability within and across images.

New images can be created from the unsupervised PixelVAE model. These are generated pixel-by-pixel from the top left corner by probabilistically sampling from the network’s learned distribution, conditioned both on previous pixels and on values in the model’s ten-dimensional (10D) latent code (Fig. 1c). The representations in this latent code are a highly compressed representation of whole-image properties and are the focus of all subsequent analyses. After training, all ten instances of the model could generate wholly novel images that look like plausible surfaces (Fig. 1d).

As a comparison DNN, we also trained ten instances of a supervised ResNet^71^ network to classify the same images as high or low gloss, using ground truth (high or low specular reflectance in the rendering parameters) as training labels. Its mean classification accuracy was 99.4 ± 0.001% (s.d.). This supervised model also contained a 10D fully connected layer prior to its two-unit output classification layer, which we treated as its high-level latent code for comparisons with the unsupervised model (Fig. 1c).

### An unsupervised generative model disentangles distal scene properties

**Fig. 2:**
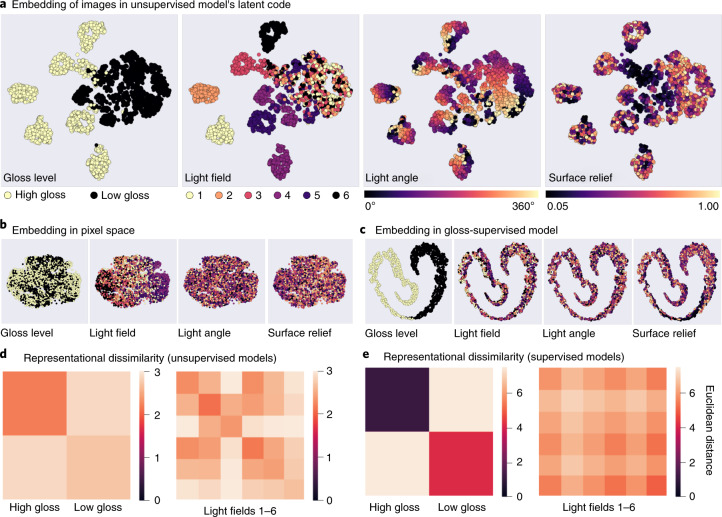
World factors are disentangled in the unsupervised model’s latent code. **a**, Visualizations of distances between 4,000 images, in the 10D latent code of one unsupervised PixelVAE network, projected into two dimensions using tSNE. Unsupervised learning spontaneously disentangles the underlying world factors, arriving at a nested representation. Images of low-gloss surfaces form one large cluster, while images of high-gloss surfaces form multiple small subclusters (left), according to the light field used to render the image (centre left). Lighting direction varies smoothly within each subcluster (centre right), and surfaces with low relief are closer to one another than are those with high relief (right). **b**, tSNE visualization of the same images in raw pixel space, where world factors are thoroughly entangled. **c**, tSNE visualization of the same images in the 10D latent code of one gloss-supervised ResNet network, showing a less rich representation. High- and low-gloss images form two clearly separated clusters, but other world factors are entangled. **d**, Dissimilarity matrices showing Euclidean distances between all pairs of images in the latent representations of all PixelVAE networks, averaged across surfaces with the same gloss level (left) or illumination (right). Pairs of images belonging to the same gloss or lighting condition (the diagonal blocks in each matrix) were represented more similarly than pairs of images belonging to different conditions (the off-diagonal blocks) (gloss: *t*_9_ = 16.73; *P* < 0.001; *d* = 0.97; 95% CI, 0.37–0.46; light field: *t*_9_ = 29.76; *P* < 0.001; *d* = 0.95; 95% CI, 0.36–0.41). **e**, Corresponding dissimilarity matrices calculated from the representations in the 10D latent codes of the supervised networks reveal stronger clustering by the task-relevant dimension of gloss and weaker clustering by light field (model × factor interaction: *F*_1,18_ = 9,878.34, *P* < 0.001, *η*^2^ = 0.99).

We are interested in the extent to which models transform raw images into a feature space within which different physical causes are disentangled^12,14,16,72^. Surfaces with similar reflectance properties may occupy very disparate points in raw pixel space but should cluster together in the feature space of a good perceptual model. Although the unsupervised PixelVAE model’s training objective deals only with proximal image data, after training on the rendered dataset, we found that distal scene properties—such as reflectance and lighting—spontaneously clustered within the networks’ 10D latent codes^47,50,73^. Visualizing in two dimensions using *t*-distributed stochastic neighbourhood embedding (tSNE)^74^ reveals that low-gloss images cluster together, while high-gloss images form multiple tight clusters, corresponding to different light fields (Fig. 2a). Within each light-field cluster, images are arranged by the angle of illumination, as well as by surface relief, with flatter surfaces occupying nearby points and bumpier surfaces more distant points. This shows that without explicit labels, the unsupervised model learns at least partially to reorganize stimuli by their physical properties, one of the core challenges of mid-level vision.

The emergence of this clustering of images by scene properties is far from trivial. It was not caused by raw image similarities, since tSNE visualization of the same images in raw pixel space showed a tight entangling of scene properties (Fig. 2b). Other linear and nonlinear pixel embeddings such as multidimensional scaling (MDS) and locally linear embedding (LLE)^75^ also failed to separate low-gloss from high-gloss surfaces (Supplementary Fig. 2). When the same visualization was applied to the 10D layer of the gloss-supervised models, high-gloss and low-gloss images were neatly separated, but other world factors were intermixed (Fig. 2c). Similar qualitative patterns held for all ten instances of both unsupervised and supervised models.

To quantify these clustering effects, we used representational similarity analysis^76^ (Fig. 2d). The results support the tSNE visualizations. Pairs of images belonging to the same gloss condition (both glossy or both matte) corresponded to closer points in the unsupervised models’ 10D latent codes than pairs of images belonging to different gloss conditions (repeated-measures *t*-test comparing average distances between same-gloss versus different-gloss image pairs, across network training instances: *t*_9_ = 16.73; *P* < 0.001; Cohen’s *d* = 0.97; 95% confidence interval (CI) of difference, 0.37–0.46). Likewise, pairs of images illuminated by the same light field had more similar latent representations than those lit by different light fields (*t*_9_ = 29.76; *P* < 0.001; *d* = 0.95; 95% CI, 0.36–0.41). In the supervised models, clustering was dominated by gloss (Fig. 2e; two-way mixed-effects analysis of variance (ANOVA) interaction between model type and scene factor: *F*_1,18_ = 9,878.34; *P* < 0.001; *η*^2^ = 0.99; follow-up tests show far stronger gloss clustering in supervised than unsupervised models, *t*_18_ = 99.39; *P* < 0.001; *d* = 44.45; 95% CI, 6.42–6.66; but stronger light-field clustering in unsupervised models, *t*_18_ = −19.90; *P* < 0.001; *d* = 8.90; 95% CI, 0.27–0.32). Thus, while the supervised model optimizes disentanglement of the single physical property on which it is trained, the unsupervised model spontaneously discovers multiple scene factors contributing to image structure.

### The unsupervised model predicts human gloss perception for novel images

**Fig. 3:**
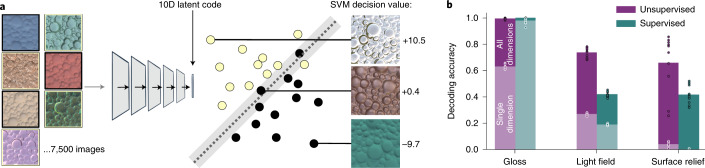
Disentangled world factors are available via a simple linear readout. **a**, To quantify how explicitly gloss was represented in each model, we trained linear SVM discriminants to classify gloss from the representations of images within the latent code of each network. As well as obtaining decoding accuracy, this allowed us to use decision values (that is, the distance from the SVM hyperplane) to make continuously valued predictions from the model regarding the gloss of novel images (three examples are shown), which was critical in subsequent experiments. **b**, Average decoding accuracy for each world factor from latent codes of unsupervised and supervised networks (the *y* axis shows the proportions correct for two-way gloss classification and six-way light field classification, as well as *R*^2^ for surface relief regression). The pale lower regions of the bars indicate the average decoding accuracy when using the best single latent dimension and give an impression of how distributed the representations are. The data points show the individual performance for each of the ten training instances of each model type (the black outline indicates full dimensionality; the white outline indicates the best single dimension).

Our central question was whether the spontaneous separation of high-gloss and low-gloss images in the unsupervised model could capture human gloss judgements. To derive quantitative gloss predictions from the models, a linear support vector machine (SVM) classifier was trained to find the hyperplane in the 10D latent code of each network that best separates high from low specular reflectance images (Fig. 3a). Although this evaluation step involves label-based decoding, it is simply a formal way of quantifying the degree and form of the disentanglement. In neuroscience, information that is available directly via a linear readout from units or neurons is generally considered to be explicitly represented by a model or brain region (for example, see refs. ^16,77–81^). The linear classifier does not provide the model with any new information but merely measures the relative placement of different classes of images within its existing feature space.

On the basis of this linear decoding, we find that gloss-classification accuracy for novel renderings across the ten unsupervised models was extremely good at 99.3% (±0.002%)—practically as good as decoding gloss from the 10D latent code of the supervised models (99.4 ± 0.002%; Fig. 3b). Light field and surface relief could also be decoded well above chance from the unsupervised networks (Fig. 3b) and significantly better than from the supervised networks (independent-measures *t*-test comparing light field decoding between unsupervised and supervised models: *t*_18_ = 23.25; *P* < 0.001; Cohen’s *d* = 10.40; 95% CI of difference, 0.28–0.34; surface relief: *t*_18_ = 3.30; *P* = 0.004; *d* = 1.48; 95% CI, 0.08–0.36). Thus, linear decoding further demonstrates that the unsupervised networks learn a compact representation that summarizes information about not only surface material but also other scene properties such as illumination and surface relief. The analysis also revealed that representations were distributed rather than sparse. The full latent code predicted scene properties much better than any individual dimension could (Fig. 3b and Supplementary Fig. 1c).

Crucially, we could now derive a predicted gloss value for any image by inputting it to a network and calculating the SVM decision value for its corresponding point in latent space (that is, the signed distance of the point from the network’s gloss-separating hyperplane; Fig. 3a). This allowed us to compare the model against human gloss perception.

**Fig. 4:**
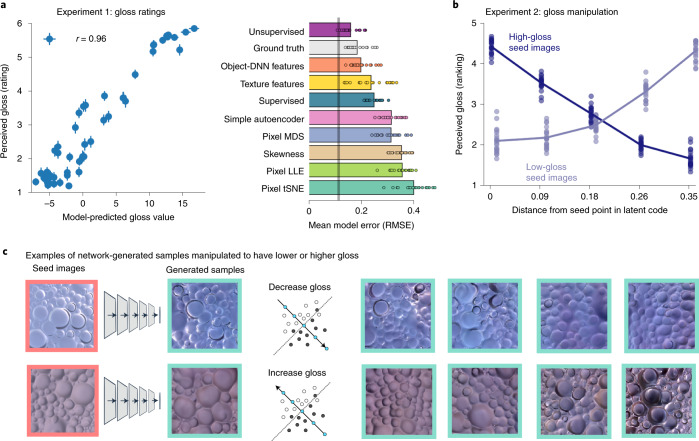
The unsupervised model can be used to modulate the gloss of generated samples, and it predicts human-perceived gloss of new surfaces. **a**, Gloss values in the unsupervised networks well predict human gloss ratings for 50 novel rendered surfaces of random gloss levels. The scatter plot shows the decision value on a glossy-versus-matte SVM classifier for each image in the PixelVAE networks on the *x* axis (averaged over ten training instances) and human gloss ratings on the *y* axis (*N* = 20). The error bars indicate the s.e.m. over 20 observers (vertical) or 10 model training instances (horizontal). The bar plot shows the average error in predicting each individual observer’s ratings, after normalizing ratings and model predictions into the range 0–1, for the unsupervised model and for diverse alternative predictors (see the text and Methods for details). The vertical grey line indicates how well individual human ratings can be predicted from the data of other observers, giving the minimum possible model error. The data points show the values for each observer. RMSE, root mean square error. **b**, Moving along the gloss-discriminating axis in a network’s latent code as shown in **c** successfully modulates perceived gloss, both when adding gloss to initially matte surfaces (pale blue line; *F*_4,76_ = 649.82; *P* < 0.001; *η*^2^ = 0.97; 95% CI for *r*, 0.97–0.98) and when removing it from initially glossy surfaces (dark blue line; *F*_4,76_ = 244.11; *P* < 0.001; *η*^2^ = 0.93; 95% CI, 0.87–0.93). The dots show each observer’s gloss rankings of each of five modulation steps, averaged over four test sequences starting from low-gloss initial seed images and four starting from high-gloss seed images. The lines indicate the mean over 20 observers. **c**, Example gloss-modulated sequences. A glossy or matte rendered image (left) is input to a network, and its 10D latent representation is recorded. New images are generated from the model, conditioned first on the original (‘seed’) point in latent space, and after stepping by small amounts along the model’s gloss-discriminating axis (that is, the axis orthogonal to the decision plane of a glossy-versus-matte SVM classifier), in either the ‘matte’ (top) or ‘glossy’ (bottom) direction.

For Experiment 1 (gloss ratings), we rendered 50 new images of surfaces with random magnitudes of specular reflectance, sampled uniformly from almost matte to almost mirror-like. Twenty observers rated the apparent gloss of each surface, from 1 (matte) to 6 (glossy). We compared their ratings with the gloss values predicted by the unsupervised model. Figure 4a shows that agreement was excellent (mean *R*^2^ over ten model training instances, 0.84) and was substantially better than for the supervised model (mean *R*^2^ = 0.40; independent-samples *t*-test *t*_18_ = 12.45; *P* < 0.001; Cohen’s *d* = 5.57; 95% CI of difference, 0.37–0.50). Notably, the unsupervised model even predicted human ratings better than ground truth (specular magnitude within the rendering engine; *R*^2^ = 0.73; one-sample *t*-test of difference, across model training instances: *t*_9_ = 4.74; *P* = 0.001; *d* = 1.50; 95% CI, 0.05–0.13).

We also considered a number of alternative models (bar graph in Fig. 4a), all of which predicted human judgements less well than ground truth. The best of these was a feature space consisting of the 1,000 final-layer features from a ResNet DNN trained on 1.2 million images to classify objects^58,71^. This is consistent with previous findings that representations in object-recognition DNNs capture perceptually relevant features of textures and images^61,82–84^. Other, less well-performing models included a multiscale texture description comprising 1,350 feature dimensions^85^; the 4,096 latent features from a relatively simple image autoencoder; 10D embeddings of raw images via tSNE^74^, MDS or LLE^75^; and luminance histogram skewness—a measure previously proposed to predict human gloss perception^86^ (Methods). Supplementary Fig. 2 shows visualizations of how reflectance and other scene factors are organized within each of these feature spaces.

Since PixelVAE networks are generative models, novel images can be generated by sampling from them (Fig. 1d). In Experiment 2 (gloss manipulation), we used such images to test whether perceived gloss varied systematically with an image’s location in the model’s latent space. From each of the model training instances, four sequences of five images were generated by conditioning the image-sampling process first on a low-gloss point in the latent space and then on progressively higher-gloss points (that is, moving along the model’s gloss-discriminating axis, orthogonal to the SVM hyperplane). Another four sequences progressed from high-gloss to lower-gloss points (Fig. 4c). The same 20 observers sorted the images within each sequence from least to most glossy. Figure 4b shows that moving along the model’s gloss-discriminating axis systematically reduced the apparent gloss of generated images when moving in the matte direction (one-way repeated-measures ANOVA: *F*_4,76_ = 244.11; *P* < 0.001; *η*^2^ = 0.93; 95% CI of correlation *r*, 0.87–0.93) and increased it when moving in the high-gloss direction (*F*_4,76_ = 649.82; *P* < 0.001; *η*^2^ = 0.97; 95% CI of *r*, 0.97–0.98).

We thus find that the unsupervised networks develop internal representations that not only disentangle distal causes that are impossible to tease apart in the raw input (Fig. 2b and Supplementary Fig. 2) but also, more remarkably, predict human gloss perception better than the true physical reflectance properties of surfaces, even though these networks are never given information about scene properties during training.

### The unsupervised model predicts failures of human gloss constancy

**Fig. 5:**
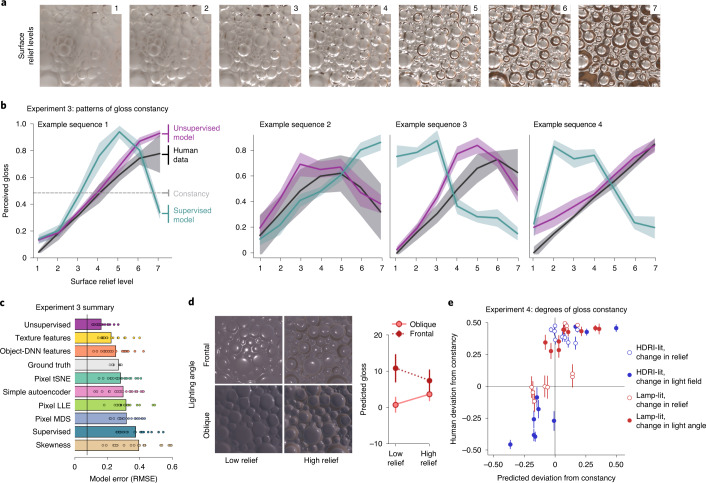
Unsupervised learning predicts human perception and misperception of gloss on an image-by-image basis. **a**, Material properties of the surface are identical in all seven images, but the surface relief increases. People often see bumpier surfaces as glossier than flatter ones, constituting a failure of gloss constancy^34,35^. **b**, Examples of various patterns of human gloss constancy failure for four sequences of images increasing in surface relief. The leftmost panel shows the data for the sequence in **a**. Black indicates the human data (the proportion of times each image step was reported as being glossier than others within each sequence), purple indicates the predictions of the unsupervised model and teal indicates the predictions of the supervised model. Veridical gloss perception would be a flat horizontal line. The shading indicates the standard deviation over 20 human observers or 10 model instances. See Supplementary Fig. 3 for the full data. **c**, Summary of the average error of each model’s prediction of each observer’s data, averaged across all 20 image sequences. The conventions are as in Fig. 4a. **d**, Changes in lighting also often change apparent material properties for human observers. The quartet of images shows the same surface material rendered on a low-relief versus a high-relief surface (left versus right columns) and illuminated straight-on versus at a 30-degree angle (top versus bottom rows). The plot on the right shows the unsupervised model predictions for these four images (the error bars indicate standard deviations over ten training instances). The model predicts a larger difference in apparent gloss for the low-relief images than for the high-relief images, and an interaction between changes in lighting and relief. **e**, Magnitude of deviations from constancy for 40 pairs of images depicting the same surface material with different scene properties (the proportion of times that image A of a pair was seen as glossier than image B). The scenes were illuminated by either natural high-dynamic-range image (HDRI) lighting (blue dots) or directional lamp (red dots). The image pairs differed in surface relief (unfilled dots), light field (blue filled dots) or lighting angle (red filled dots). The *y* axis shows the average human data over 20 observers, expressed in terms of raw proportion minus 0.5, so that zero indicates good constancy, +0.5 indicates that image A always ranked glossier than image B and −0.5 indicates the opposite. The error bars indicate the s.e.m. over observers. The *x* axis shows the difference in predicted gloss values between the images in each pair, averaged over ten training instances of the PixelVAE model.

Although human gloss perception generally aligns well with specular reflectance, it also exhibits some well-documented ‘errors’. For example, bumpier surfaces tend to look glossier than flatter surfaces, and specific combinations of lighting and surface relief yield specific patterns of misperception (Fig. 5a and refs. ^34,35^). Mimicking such perceptual errors is a key test of any computational model of biological vision^87^. We assessed how well different models capture the systematic failures of gloss constancy exhibited by human observers.

To do this, we rendered sequences where surface relief increased in seven steps, while reflectance and other scene properties remained fixed (Fig. 5a). For stimuli in Experiments 3a and 3b (patterns of gloss constancy), we selected two sets of ten sequences for which both the unsupervised and supervised models predicted deviations from constant gloss and the models made different predictions about the particular pattern of deviations (Methods). The rationale behind this is that cases where models disagree provide the strongest power to test which model is superior^88,89^.

With these image sequences in hand, in Experiments 3a and 3b, two groups of 20 observers judged gloss in a paired-comparison task (Methods). If the observers correctly estimated reflectance, all surfaces should appear equally glossy, yet we find that they do not. The observers exhibited strong failures of gloss constancy, usually reporting surfaces with deeper relief to be glossier, although perceived gloss was non-monotonic in 7 of the 20 sequences, being the highest for intermediate reliefs (four examples are shown in Fig. 5b; the complete data are shown in Supplementary Fig. 3). The unsupervised model, despite never being explicitly trained to represent gloss and without being fit to human data, predicted patterns of failures of gloss constancy remarkably well (median *R*^2^ across sequences and model training instances, 0.71). The model correctly predicted the qualitative pattern of constancy failure (monotonic versus non-monotonic) for 18 out of the 20 stimulus sequences. In contrast, the supervised model completely failed to predict human gloss constancy. For almost all sequences, it made predictions that were anticorrelated with human patterns (median *R*^2^, –1.45). Of the alternative models (Fig. 5c), a set of mid-level texture features^85^ provided the next-best performance (median *R*^2^, 0.54) but was significantly poorer than the unsupervised model (one-sample *t*-test of difference across model training instances: *t*_9_ = 10.48; *P* < 0.001; Cohen’s *d* = 3.31; 95% CI of difference, 0.14–0.20).

Human gloss constancy ranges from good to bad depending on interactions between lighting and shape^30,33–35,90^. For Experiment 4 (degrees of gloss constancy), we rendered 40 image pairs depicting surfaces with identical material but different surface relief or lighting (Fig. 5d and Methods), for which the unsupervised model predicted a wide range of degrees of gloss constancy, from excellent (near-identical predicted gloss for both images in a pair) to very poor (the images received very different predicted gloss values; see Methods).

Twenty observers indicated which surface in each pair appeared glossier, with each pair repeated eight times. The unsupervised model predicted the degree and direction of human (failures of) constancy reasonably well (mean *r* across model training instances, 0.70; Fig. 5e) and outperformed all alternative models (the next-best model was object-DNN features, *r* = 0.64; one-sample *t*-test of difference, across PixelVAE training instances: *t*_9_ = 4.00; *P* = 0.003; Cohen’s *d* = 1.27; 95% CI of difference, 0.03–0.08). Fitting a simple logistic function to relate model and human gloss values further improves the prediction (from an average *R*^2^ across model training instances of 0.60 for a linear fit, to an *R*^2^ of 0.74 for a logistic fit).

**Fig. 6:**
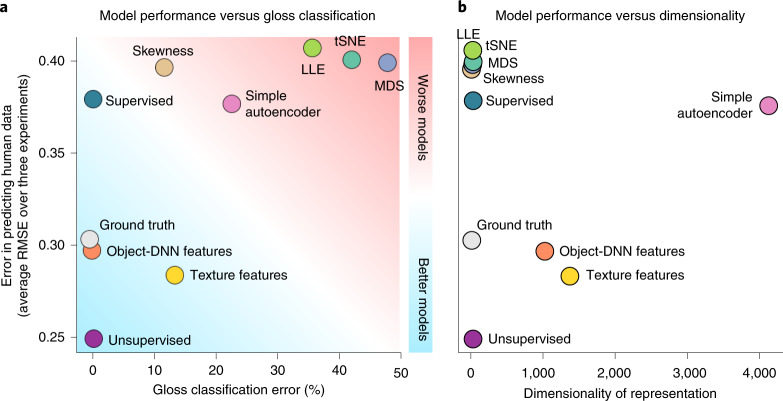
The unsupervised model outperforms diverse alternative models. **a**, Relationship between error at classifying surfaces as glossy or matte (*x* axis) and error in predicting human psychophysical gloss judgements (*y* axis) for all models. The data are averaged over the three psychophysical experiments in which the ground truth gloss of stimuli is defined (Experiments 1, 3 and 4, each with *N* = 20). **b**, Relationship between the dimensionality of each model’s representation and its error in predicting human judgements (the *y* axis is shared with **a**). Not only does the unsupervised PixelVAE model best explain the human data (difference from the next-best model: *t*_59_ = 10.73; *P* < 0.001; Cohen’s *d* = 1.39; 95% CI, 0.05–0.07), but it does so with around two orders of magnitude fewer dimensions than the nearest competing models (a 1,350-dimensional texture feature space^85^ and a 1,000-dimensional layer from an object-recognition supervised DNN^57^).

Aggregating the results across the three experiments using renderings (Experiments 1, 3 and 4) shows that the unsupervised model predicts human perceptual judgements better than all others (Fig. 6). It achieves near-perfect ground-truth gloss-classification accuracy while still predicting idiosyncratic errors of human gloss perception (Fig. 6a), with a feature space two orders of magnitude more compact than the next-best model (Fig. 6b). The next-best model was a set of texture features hand-engineered to efficiently capture higher-order statistical structure in images^85^, which performed significantly less well (one-sample *t*-test of difference in root mean square error predicting individual data across the three experiments: *t*_59_ = 10.73; *P* < 0.001; Cohen’s *d* = 1.39; 95% CI, 0.05–0.07). Simple image statistics, such as the skewness of the luminance histogram^86^, failed to capture human gloss perception under our deliberately challenging stimulus conditions, where differences in specular reflectance must be de-confounded from differences in lighting and surface shape.

### Model generalization and effects of training set

**Fig. 7:**
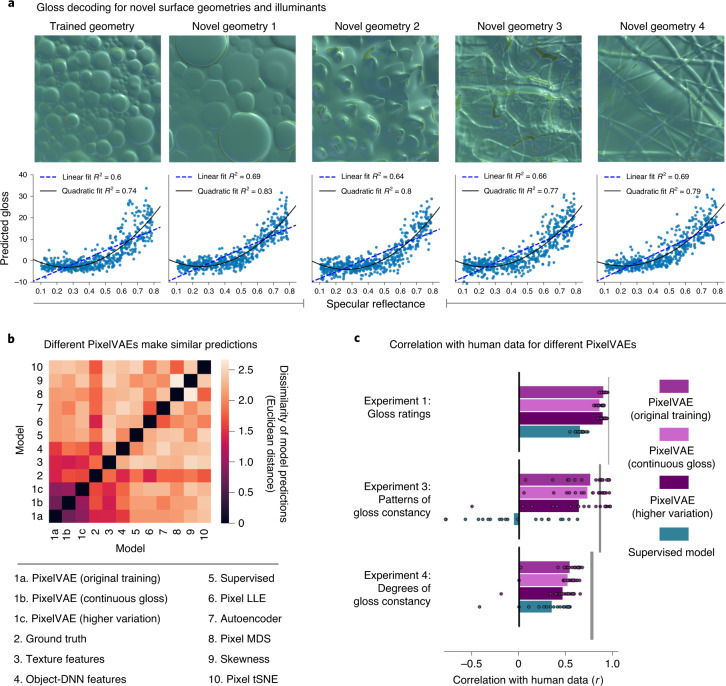
The unsupervised model generalizes well to novel scenes and makes similar gloss predictions after retraining on different visual environments. **a**, Scatter plots show predicted gloss (*y* axis) and ground-truth specular reflectance (*x* axis) for sets of 500 new images rendered with random continuously sampled gloss levels, using the trained surface geometry (leftmost panel) or four novel geometries unseen during training (right panels). A single example image from each set is shown above the plots (magnitude and concentration of specular component, 0.65). All scenes were illuminated by light fields never seen during training. **b**, New instances of the PixelVAE model with substantially different visual experiences during training all make similar predictions for the gloss levels of experimental stimuli. The matrix shows the dissimilarity between the gloss predictions made by the original set of ten PixelVAE networks, averaged over training instances (1a); five PixelVAE networks trained on a new set of 10,000 images using the same surface geometry and light fields, but with specular reflectance continuously sampled, averaged over training instances (1b); a PixelVAE network trained on a third set of 10,000 images using ten surface geometries and 50 light fields, non-overlapping with the geometry and light fields used in the main training dataset (1c); and alternative models, ordered by the similarity of their predictions to that of the original PixelVAE (2–10). The gloss predictions are for all stimuli used in Experiments 1, 3 and 4 and were normalized into the range 0–1 for comparability across models. **c**, Average correlation between model-predicted gloss and individual human judgements in each psychophysical experiment for the PixelVAE implementations described in **b**. The data points show the values for each observer (*N* = 20 in each experiment). All unsupervised models predicted the human data better than the supervised model in all experiments (across nine comparisons: *t*_19_ = 4.01–31.08; *P* < 0.001; Cohen’s *d* = 0.56–7.00; 95% CI, 0.06–0.18 to 0.60–1.07). The vertical grey lines indicate how well the data can be predicted on average from those of other observers, giving the highest possible average model correlation.

The composition of the training dataset has profound effects on all machine learning models. However, a good model of human mid-level vision should generalize over changes to training or test data. Our unsupervised networks were trained on a simulated environment with bimodally distributed specular reflectance (near-matte or high-gloss). Nevertheless, they well predicted gloss in new image sets containing continuously varying reflectance (Fig. 7a; mean *R*^2^, 0.79; s.d. of *R*^2^, 0.03; see also Supplementary Fig. 6a), and they predicted gloss for scenes with novel geometries and light fields as well as they did for familiar scenes (Fig. 7a).

Model predictions do not seem to depend on artefacts of the specific computer graphics techniques used to generate our images (real-time rasterized rendering), as gloss predictions were near identical for matched surfaces rendered using more time-intensive but physically faithful ray-traced rendering (Supplementary Fig. 6b). Remarkably, given their constrained training environments, the models even seem able to broadly categorize close-up photographs of real-world surfaces^91^ as being of high or low gloss, although they fail when shown surfaces far outside their training data, such as fabrics with high-contrast patterns (Supplementary Fig. 6c,d).

We performed two tests of robustness to different training datasets. First, five new PixelVAE networks were trained on 10,000 additional renderings in which gloss was sampled continuously rather than bimodally (continuously sampled gloss training dataset; Methods). Second, an additional PixelVAE was trained on a third dataset of 10,000 renderings in which surface geometry and lighting varied far more widely (higher-variation training dataset; Methods), with each scene comprising one of ten novel surface geometries combined with one of 50 novel light fields. Both new training environments produced models with latent codes that could well subserve gloss classification on the original bimodal gloss dataset (with mean accuracies of 96.7% and 91.4%, respectively). Importantly, we found that all three versions of the PixelVAE model (original, continuous-gloss training and higher-variation training) made highly similar predictions regarding the relative gloss levels of experimental stimuli. This indicates that the ability to predict human perception is not highly sensitive to the training set. The three versions of the unsupervised PixelVAE model, trained on non-overlapping datasets, made more similar gloss predictions to one another than to those made by any of the ten diverse alternative models (the dark cluster of low dissimilarity values in the bottom left of Fig. 7b). All three training environments led to unsupervised models that predicted human data reasonably well (Fig. 7c), both for gloss ratings of novel rendered images (Experiment 1) and for the more challenging task of predicting patterns (Experiment 3) and degrees (Experiment 4) of (failures of) gloss constancy. Each of the three model versions predicted human data significantly better than the supervised model in all experiments (*t*_19_ = 4.01–31.08; *P* < 0.001; Cohen’s *d* = 0.56–7.00; 95% CI, 0.06–0.18 to 0.60–1.07 in nine repeated-measures *t*-tests comparing unsupervised versus supervised model correlation with individual participants’ data; Bonferroni-corrected *α*, 0.006) and were significantly better than the most promising alternative model, texture features^85^, in six out of nine comparisons (*t*_19_ = 0.73–5.84; *P* = 0.47 to <0.001; *d* = 0.17–1.16; 95% CI, −0.12–0.25 to 0.12–0.31).

Several analyses were performed to assess how robustly unsupervised models outperformed their supervised counterparts. In building and training a DNN, values must be chosen for the many hyperparameters controlling network architecture and training. We evaluated the effects of some of these hyperparameters by training 28 additional models (14 unsupervised and 14 supervised) that differed from the original implementations in depth, learning rate, learning rate decay, training batch size and complexity of the learned model (for unsupervised PixelVAEs); see Supplementary Table 1 for the details. Eleven of the 14 unsupervised network variants outperformed all supervised network variants in predicting human gloss judgements; the three exceptions were networks that failed to train due to poor learning rate settings (Supplementary Fig. 5). We also found that representations in the unsupervised model better predicted human judgements than those in the supervised model for all intermediate layers (Supplementary Fig. 4a). Finally, we created a version of the supervised model that outputs continuous-valued gloss estimates rather than categorical decisions and trained it on a dataset with continuous rather than bimodal reflectances (Supplementary Methods and Results). This version predicted human gloss judgements better than the category-supervised model but less well than the unsupervised model, failing to exhibit the systematic errors that characterize human gloss perception (Supplementary Fig. 4b). Overall, unsupervised learning in PixelVAE models seems to converge on a representation that captures key aspects of human gloss perception and tolerates changes in the particular network hyperparameters, or the statistics, illuminations or geometries of the training and test sets.

### Features underlying gloss representation in the model

**Fig. 8:**
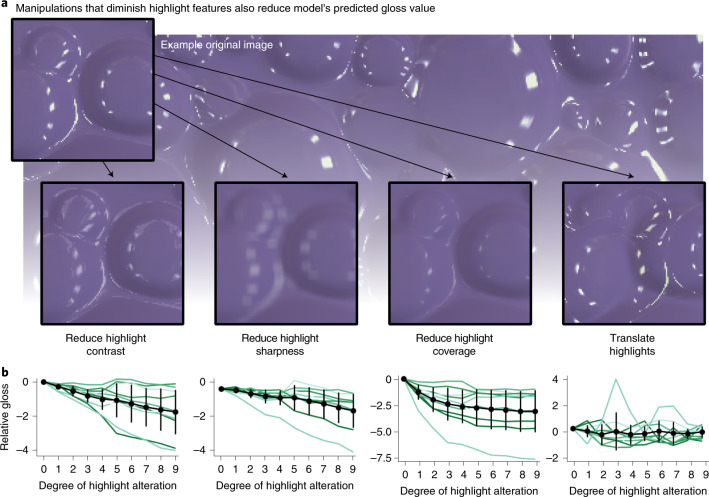
Reducing the coverage, sharpness or contrast of highlights reduces model-predicted gloss. **a**, We separately rendered the specular and matte components for ten probe images and manipulated the specular component by reducing contrast, by blurring it, by shrinking coverage via an image erosion algorithm or by laterally translating it, in ten progressive steps. We then recombined the two components to produce ten sequences of images and presented them to the unsupervised networks. The insets show each of these manipulations applied to part of an example image. **b**, Model-predicted gloss declined when any of the three highlight features was reduced (left three plots; contrast: *F*_9,81_ = 11.20; *P* < 0.001; *η*^2^ = 0.55; 95% CI of *r*, −0.65 to −0.41; sharpness: *F*_9,81_ = 9.65; *P* < 0.001; *η*^2^ = 0.52; 95% CI, −0.61 to −0.40; coverage: *F*_9,81_ = 18.14; *P* < 0.001; *η*^2^ = 0.67; 95% CI, −0.58 to −0.29). The green lines show the predicted gloss for each step in each sequence, relative to that of the original unmanipulated image, averaged over ten training instances of the model; the black lines show the mean and standard deviation over all sequences. Translating the specular components (rightmost plot), which does not affect the coverage, sharpness or contrast of the highlights, did not measurably affect predicted gloss (*F*_9,81_ = 0.48; *P* = 0.88; *η*^2^ = 0.05; 95% CI, −0.21–0.09).

Previous research^35^ identified specific image features—the coverage, contrast and sharpness of specular highlights—that predicted perceived gloss for surfaces like those evaluated here. To test whether the PixelVAE model was also sensitive to these cues, we measured the coverage, contrast and sharpness of highlights in 10,000 new renderings of surfaces with specular reflectance varying continuously from near-matte to near-mirror. All three cues could be decoded from the latent code of a PixelVAE trained on these images (mean *R*^2^ = 0.71) and could be increasingly well decoded from successive convolutional and fully connected layers (Supplementary Fig. 7a). A linear combination of the three cues in the layer immediately preceding the 10D latent code correlated with gloss predicted from the latent code (*r* = 0.80; Supplementary Fig. 7b). Moreover, manipulating the highlights in images to weaken each cue also reduced predicted gloss (Fig. 8a,b; one-way repeated-measures ANOVA for the effect of highlight contrast reduction: *F*_9,81_ = 11.20; *P* < 0.001; *η*^2^ = 0.55; 95% CI of correlation *r*, −0.65 to −0.41; sharpness: *F*_9,81_ = 9.65; *P* < 0.001; *η*^2^ = 0.52; 95% CI of *r*, −0.61 to −0.40; coverage: *F*_9,81_ = 18.14; *P* < 0.001; *η*^2^ = 0.67; 95% CI of *r*, −0.58 to −0.29).

Interestingly, we did not find evidence that the model’s predicted gloss decreased when the specular component of images was shifted so that highlights were misaligned with the geometry of the depicted surfaces (Fig. 8b; *F*_9,81_ = 0.48; *P* = 0.88; *η*^2^ = 0.05; 95% CI of *r*, −0.21–0.09). This manipulation preserves coverage, contrast and sharpness, yet infringes photogeometric constraints that are a precondition for humans to identify highlights as specularities and therefore to see gloss at all^6,92,93^. The fact that the model predicts human gloss constancy without seeming to be sensitive to such constraints suggests that although these constraints are crucial to many aspects of surface perception^7,93–96^, the degree of perceived gloss, within surfaces that are seen as having highlights, may be largely explainable in terms of image features.

## Discussion

The efficient representation of sensory data has long been hypothesized as a central goal of neural coding^15,19–24,97^. But while such approaches predict many aspects of low-level image encoding, they have not explained how we visually infer properties of the outside world. Unsupervised learning objectives in modern DNNs, such as data compression and spatial prediction, offer powerful new implementations of these statistical learning principles^17^. Our findings show that mid-level perceptual dimensions, such as gloss—which imperfectly map onto properties of the physical world—can emerge spontaneously by learning to efficiently encode images. Unsupervised learning may thus provide a bridge that links theoretical views emphasizing the importance of image statistics (for example, see refs. ^86,98,99^) to those that treat perceptual processes as a decomposition of images into distinct physical causes (for example, see refs. ^4,6,8,100^).

One of the fundamental unsolved questions in vision science is how the visual system became aware of the different physical sources that contribute to image structure. Perception is commonly framed as the optimal estimation of a set of known physical quantities^4,9–11^. But these quantities that the brain putatively estimates were not specified a priori; they must somehow be discoverable (over either the course of evolution or learning) on the basis of their manifestation in sensory experience^12–14^. Here, we suggest that different physical causes give rise to different high-order regularities in visual data, making them discoverable through data-driven unsupervised learning processes^15–18^. We provide a proof-of-principle that it is possible to learn to disentangle distal causes without prior knowledge about which classes of causes exist in the world, the cues that could be used to distinguish them or even how many different classes of causes there are. An unsupervised statistical learning model predicted the expected changes in perceived gloss caused by varying specular reflectance (Experiment 1; refs. ^32,33,39–41,43^), as well as illusory changes in perceived gloss that arise from varying lighting and shape (Experiments 3 and 4; refs. ^34,35^). We suggest that known systematic errors in gloss perception can be attributed to the particular pattern of partial disentanglement arising from unsupervised statistical learning of surface appearances.

One of our more intriguing results is that the unsupervised model predicted human perception better than the supervised model that we tested. It is important to note that this is not because humans and unsupervised networks were better at extracting ground truth in these stimuli. Categorization-supervised networks categorized gloss almost perfectly (Fig. 3b), and regression-supervised networks predicted continuous gloss levels almost perfectly (Supplementary Fig. 4b), yet both predicted human judgements less well than unsupervised networks. This implies that the systematic errors exhibited by humans and the PixelVAE model are not a trivial consequence of some inherent impossibility in recovering specular reflectance for these stimuli. Nor is this result explained by the supervised model reporting ground-truth specular reflectance too faithfully. Although the supervised model is near-perfect at coarsely categorizing surfaces as having high or low specular reflectance, it still predicts different degrees of glossiness for different images, including sometimes erroneously. Yet the supervised and unsupervised models make different predictions on an image-by-image basis, with the latter more closely matching those made by humans. We propose that this shared pattern of deviation from ideal performance may arise from shared characteristics in how the human visual system and the unsupervised model learn to encode images.

One of the most notable failures of the PixelVAE in capturing human data is its insensitivity to photogeometric constraints known to affect human surface perception, such as the alignment of specular highlights with diffuse shading^7,92,93^ (Fig. 8). We believe that this failure is probably due to the relative poverty of 3D shape information in its training set. The link between highlights and diffuse shading arises from constraints imposed by the 3D shape of a surface^94,96^. It seems implausible to expect any visual system trained solely on monocular, static images to develop good sensitivity to these constraints, and without a detailed representation of 3D shape, no model is likely to explain all aspects of human gloss perception^94,95,101^. We tailored the training sets towards modelling variations in perceived glossiness for physically realistic surfaces, where highlights are assumed to align with surface shading^32,33,39–41,43^. An important direction for future research is testing whether unsupervised DNNs can also learn photogeometric relationships, if training sets provide additional information about shape (for example, through motion, stereo, occlusion or larger variations in geometry).

In using deep learning models, we do not wish to imply that all material perception is learned during an individual lifetime. Unsupervised learning principles can also operate on an evolutionary timescale. For example, V1 cell receptive fields are predicted by simple unsupervised learning models such as independent components analysis^22^, and these seem to be present at birth in macaques^102^. The developmental trajectory of gloss perception is not fully known. There is evidence that five- to eight-month-old infants can distinguish between matte and specular surfaces^103^, but also evidence that material recognition and classification are still developing in five- to ten-year-old children^104,105^. Even five to eight months of visual experience provides a huge dataset for statistical regularity learning^106^. It could be that approximate versions of many perceptual dimensions are rapidly learned during the first days, weeks and months of life.

Although we do not propose the PixelVAE architecture as a physiological simulation of visual cortex, its computational principles are eminently achievable in biological brains. Data compression and spatial prediction are learning objectives that require no additional information beyond the incoming sensory data, and there are several mechanisms by which brains could represent the probability distributions used in the PixelVAE network^107–109^. At the same time, the brain certainly does not learn statistical distributions over RGB pixels. If the visual system uses learning objectives like the ones investigated here, they presumably operate on image representations that have undergone substantial retinal processing^110,111^.

In conclusion, unsupervised DNN models provide an ecologically feasible solution to the problem of how brains come to represent properties of the distal world without access to ground truth training data^12–14,17,112^. Nonlinear transformations reorganize inputs according to high-order regularities within and across images, allowing the visual system to better summarize and predict sensory data. Because regularities in images are caused by underlying physical objects and processes, these new configurations often end up (partially) disentangling physical properties from one another. Our results suggest that the imperfect nature of this disentanglement may account for the characteristic errors that humans make. Failures of constancy, which are rife in vision, may therefore offer clues to how we learn to see. Unsupervised learning may account for these failures not just in gloss perception but in perception more broadly.

## Methods

### Participants

Three groups of human naive observers reported perceived gloss across five experiments: Experiments 1 and 2 (*N* = 20; mean age, 23.45; age range, 19–32; 16 female, 4 male), Experiment 3a (*N* = 20; mean age, 23.45; age range, 19–31; 16 female, 4 male) and Experiments 3b and 4 (*N* = 20; mean age, 24.45; age range, 19–35; 14 female, 6 male). Six individuals participated in two different experimental groups but received no information about the experimental design or hypotheses after the first session. No statistical methods were used to predetermine sample sizes, but our sample sizes are larger than those reported in previous publications measuring gloss constancy^34,35,37^. All participants had normal or corrected-to-normal visual acuity. Two male participants self-reported poor red–green colour vision. The experiments were conducted in accordance with the Declaration of Helsinki (sixth revision), with prior approval from the ethics committee of Justus Liebig University, Giessen, Germany. The volunteers gave written informed consent and were paid €8 per hour.

### Stimuli

The stimuli were 800 pixel × 800 pixel images of bumpy surfaces rendered using Unity3D (v.2018.2.3; Unity Technologies, https://unity.com/). A virtual 40 cm × 40 cm sheet with irregularly positioned bumps was placed in a scene and illuminated by one of six high-dynamic-range image (HDRI) light probes. The light probes were white-balanced 8,192 pixel × 4,096 pixel images of four exterior (beach, bay, woodland and savannah) and two interior (church and conference hall) environments, captured by Unity Technologies (https://assetstore.unity.com/packages/2d/textures-materials/sky/unity-hdri-pack-72511). A virtual camera (60° field of view) hovered 12 cm above the sheet, viewing directly downwards. By randomly varying the camera’s location and orientation, an extremely large number of unique images could be generated from the scene.

#### Main training dataset

We rendered 10,000 images to create the main training dataset for the neural network models. For each rendering, one of the six HDRIs was randomly selected to illuminate the scene. Surface relief was scaled multiplicatively in depth by a uniformly sampled random scaling factor, so that the distance between the lowest and highest points on the surface was between 0.1 cm and 2.5 cm. The surface’s reflectance properties were controlled via the Unity3D standard shader, using the ‘specular setup’. The diffuse reflectance component of the surface material was chosen by selecting RGB values randomly uniformly within the interval 0.3 to 0.7, independently for each channel. The surface was either low or high gloss, with equal probability. For low-gloss surfaces, the specular reflectance component of the material was selected randomly uniformly between 0.1 and 0.3, and the concentration of the specular component was selected randomly uniformly between 0.2 and 0.4. For high-gloss surfaces, specular reflectance was between 0.3 and 0.5, and specular concentration was between 0.75 and 0.95. The training dataset therefore had a bimodal distribution of approximately 50% low-gloss and 50% high-gloss surfaces, with small variations in reflectance properties within each group. The same dataset was also used when training classifiers to decode gloss and other world factors from models.

#### Continuously sampled gloss training dataset

To verify that the representations learned by our models were not artefacts of the bimodal gloss sampling in the main dataset, we rendered 10,000 new images in which both the magnitude and concentration of the specular reflectance component were sampled randomly uniformly between 0.1 and 0.8, independently of each other. All other scene properties were varied as in the main dataset.

#### Higher-variation training dataset

To verify that less-constrained visual diets could lead to similar gloss representations, we created a third training dataset in which 10,000 images each randomly combined one of ten novel surface geometries with 50 novel light fields. The novel geometries were virtual 40 cm × 40 cm sheets with different shapes and sizes of irregularly placed ridges, bumps and indentations (examples are shown in Fig. 7a). The novel light fields were 4,096 pixel × 2,048 pixel HDR images (25 exterior and 25 interior) from the HDRI Haven database (http://www.hdrihaven.com). The distance of the virtual camera above the surface varied randomly uniformly between 8 cm and 12 cm to introduce scale variation. All other scene properties varied as in the main dataset.

#### Renderings with continuously sampled gloss levels (Experiment 1: gloss ratings)

The stimuli were 50 new rendered images with specular reflectance chosen randomly uniformly between 0.2 and 1.0, and specular concentration set to the same value. Other attributes were varied as in the main dataset. For all rendered images used as stimuli in the psychophysical experiments (Experiments 1, 3 and 4), the same geometry and set of light fields were used as in the main training dataset, but the images were novel renderings unseen by any model during training.

#### Gloss-modulated images generated from PixelVAE models (Experiment 2: gloss manipulation)

For each of the PixelVAE networks, we first determined the axis in 10D latent space along which gloss could be most strongly discriminated (‘Data analysis’). Eight images (four low-gloss and four high-gloss) from the main training dataset were input to each network, and their corresponding latent points were recorded. These were used as seed points to generate eight gloss-modulated sequences for each network. The first step in each sequence was generated by conditioning the model’s pixel-sampling process on the respective seed point in latent space and then sampling a 128 pixel × 128 pixel image (‘Neural network architectures and training’). The conditioning point was then moved 0.07 units in latent space along that model’s gloss-discriminating axis, in either the matte (for high-gloss seed images) or glossy (for low-gloss seed images) direction, and a second image was generated. This was repeated three more times, yielding a five-step sequence for each seed image from each model training instance (400 experimental images in total).

#### Sequences of renderings with increasing surface relief (Experiments 3a and 3b: patterns of gloss constancy)

To create a strong test of the different models, we wanted to probe human gloss constancy using stimuli for which there were clear differences in constancy patterns predicted by unsupervised versus supervised models^88,89^, and which were likely to produce diverse patterns of failures of constancy in human observers. We therefore first generated candidate stimuli and then selected those that best satisfied these desiderata.

For each experiment, we rendered 200 different candidate sequences of seven images with fixed random material, illumination and camera position, but exponentially increasing surface relief (maximum bump heights of 0.10, 0.18, 0.31, 0.55, 0.97, 0.70 and 3.0 cm). All surfaces had relatively high gloss. In Experiment 3a, specular reflectance was selected randomly uniformly between 0.2 and 1.0, and specular concentration was between 0.7 and 1.0. In Experiment 3b, reflectance was between 0.1 and 0.3, and concentration was between 0.8 and 1.0. Other attributes were varied as in the main dataset. All 1,400 images in each set were input to each of the unsupervised PixelVAE and supervised ResNet networks to obtain predicted gloss values (‘Data analysis’). For each candidate sequence of seven images, we then simulated the responses from each network under a two-alternative forced-choice (2AFC) experiment in which each possible pair of images was compared, and the image for which the model predicted a higher gloss value was selected. This was analogous to the ‘Which is more glossy?’ task performed by human observers (‘Psychophysical procedures’).

First, for each sequence we performed a 2 × 7 (model × surface relief) ANOVA between unsupervised and supervised model predictions, averaged over the training instances of each model. The sequences were sorted according to the *F* interaction statistic, prioritizing those with strong disagreements between model predictions^88,89^. In Experiment 3a, we visually inspected the top-ranked sequences and selected ten with diverse appearances and subjective failures of gloss constancy.

In Experiment 3b, we selected test sequences in an entirely automated manner by classifying the sequences into four qualitative groups on the basis of the average constancy pattern predicted by the unsupervised models:

- Group 1: the model predicts approximate constancy (range of predicted ‘proportion glossier’ values < 0.25, and a linear function fit achieved *R*^2^ > 0.70);
- Group 2: the model predicts an approximately linear increase in gloss with bump height (range > 0.50, and a linear fit achieved *R*^2^ > 0.90);
- Group 3: the model predicts a non-monotonic failure of constancy (range > 0.50, and a quadratic fit of *R*^2^ > 0.90 with a negative coefficient of the squared term);
- Group 4: the model predicts an upward-swinging nonlinear failure of constancy (range > 0.50, and a quadratic fit of *R*^2^ > 0.90 with a positive coefficient of the squared term).

We then selected the top sequences in each group (ranked by the *F* interaction term), in proportion to the size of each group (one constant, four linear, two non-monotonic and three nonlinear sequences).

#### Pairs of renderings with differing surface relief or lighting (Experiment 4: degrees of gloss constancy)

Here we sought pairs of stimuli with diverse scene differences (some differing in surface relief, some in lighting environment and some in angle of illumination) and for which the unsupervised model made a wide range of predictions, ranging from good constancy to strong failure of constancy.

We rendered 800 candidate image pairs with the same random material properties and camera position, but differing in either surface relief (0.40 cm versus 1.5 cm maximum bump height) or illumination. Pairs differing in illumination were either ‘naturally’ lit (differing in HDRI lighting environment, from among the six used in the main training dataset) or illuminated by a directional lamp (at a 30° versus 90° angle). Candidate pairs were generated in four groups of 200:

- Group 1: identical natural light field; different surface relief;
- Group 2: identical lamp angle; different surface relief;
- Group 3: identical surface relief; different natural light field;
- Group 4: identical surface relief; different lamp angle.

Average predicted gloss values were obtained for each image, across training instances of the unsupervised PixelVAE model (‘Data analysis’). Within each group, we ranked pairs by the absolute difference in predicted gloss of the images in the pair, and we selected pairs lying at each of the 10th percentiles, yielding 40 test pairs.

#### Geometry and lighting generalization test set

To test the generalization of models trained on the main dataset, we rendered five sets of 500 new images, one using the original surface sheet and four using novel surface geometries (Fig. 7a). Illumination was randomly one of eight novel 4,096 pixel × 2,048 pixel HDR light probes (six exterior and two interior) from the Dutch Skies 360° HDRI Project (http://www.dutch360hdr.com/). Specular reflectance was varied as in the continuously sampled gloss training dataset.

#### Highlight-feature manipulated images

To manipulate highlight features (Fig. 8), we manipulated the specular component image in four different ways, before combining with the diffuse component image by addition:

- Translated highlights: the specular component was shifted rightwards in ten five-pixel steps. To avoid edge artefacts after translation, the images were rendered at 1,024 × 1,024 resolution and then cropped to the lower-right 800 pixels × 800 pixels.
- Reducing highlight coverage: we applied image erosion to the specular component using the OpenCV package for Python. The kernel size was 2 × 2. Up to ten iterations were applied to create ten progressively reduced highlight maps.
- Reducing highlight sharpness: the specular image was convolved with a Gaussian filter, with the step size ranging from 1 × 1 to 11 × 11.
- Reducing highlight contrast: we created a ‘highlight mask’ by identifying pixels with values >4 (from a range of 0–255) in the greyscale version of the specular image. The standard deviation of pixel intensity within the highlight map was multiplicatively reduced in ten steps, from 1 to 0.1 times their original s.d., while retaining the original mean.

### Neural network architectures and training

The DNNs were implemented and trained in Python v.2.7 (Python Software Foundation, https://www.python.org/) with Tensorflow v.1.14 (https://www.tensorflow.org/) on a GPU-accelerated machine using 1-4 GTX1080 graphics cards. The networks were trained on the first 9,000 images of the main training dataset, using the next 500 for validation and the final 500 for testing the accuracy of the supervised networks. All images were input at a resolution of 128 pixels × 128 pixels. For both unsupervised and supervised models, ten independent instances of the same architecture were trained from different random initial weights and with different random sampling of training batches, to ensure robustness to representational differences between training instances^70^. We also trained five instances of the PixelVAE network on the continuously sampled gloss dataset and one on the higher-variation dataset (‘Stimuli’), dividing training and validation data in the same proportions. No data augmentation was used. All architectures used rectified linear activation functions.

#### Unsupervised PixelVAE model

We used the implementation from ref. ^47^ of the PixelVAE architecture^48^, available at https://github.com/ermongroup/Generalized-PixelVAE. The architecture consists of two streams of convolutional layers, which learn jointly via backpropagation. One stream is a ‘conditioning network’, which is a convolutional variational autoencoder that takes an image as input and outputs a 10D smooth latent representation. We chose a 10D latent code as being approximately the most compact representation that still allowed the network to learn a good model of the training images, on the basis of pilot data (Supplementary Fig. 1a,b). The other stream is an autoregressive PixelCNN++^113,114^ model that learns the structure of the training data in terms of a logistic mixture probability distribution over pixel values and takes as inputs both the image and the latent code output by the conditioning network. To generate new images, the autoregressive stream chooses an RGB value for each pixel, working from the top left to the bottom right of the image. Each pixel is sampled from the learned probability distribution, conditioning both on the values of pixels generated so far (which constrain the local structure of the image) and on the values provided in the latent code (which constrain more holistic image properties).

The conditioning network consisted of three convolutional layers of 64, 128 and 256 feature maps, followed by a fully connected layer of 512 units and a 10-unit latent code layer. The autoregressive network consisted of six ‘residual blocks’ of three layers of 64 convolutional feature maps each, with skip connections linking the first and sixth, and second and fifth blocks. Pixel likelihood distributions were modelled with a mixture of 12 logistic functions, and the networks were trained with a batch size of 5 and a learning rate of 0.001 for 200 epochs, around which point the validation error plateaued. The learning rate was gradually decayed by multiplying by 0.999995 after each epoch. No regularization was used during training, to encourage the network to depend on information in its latent code^47^.

#### Supervised ResNet model

We used the Tensorflow implementation of the ResNet architecture^57^ from https://github.com/wenxinxu/resnet-in-tensorflow. The networks consisted of three residual blocks each made up of three layers of 56 convolutional feature maps, with skip connections between each. The output of the final layer was passed to a ten-unit fully connected layer, which we treated as the ‘latent code’ of the model for analysis purposes. This passed, via one more nonlinearity, to a two-unit softmax output layer. Ten networks were trained, from different random initial weights, to classify images as renderings of surfaces with high or low specular reflectance (‘Stimuli’). The networks were trained with a batch size of 32 and a learning rate of 0.001 to minimize the sparse softmax cross-entropy between outputs and correct labels. The learning rate was gradually decayed by multiplying by 0.99995 after each epoch. The networks were trained for 21 epochs, by which point the validation accuracy plateaued above 99%.

#### Simple autoencoder

We also considered a far simpler unsupervised model, in the form of a non-variational convolutional autoencoder implemented using Keras v.2.2.5 (https://keras.io/) for Tensorflow. The architecture comprised four layers of 64, 32, 16 and 16 feature maps alternating with 2 × 2 pooling layers, leading to a 4,096-unit fully connected bottleneck layer, which we treated as the model’s latent feature space for analysis purposes. The compressed code was expanded through mirrored layers of 16, 16, 32 and 64 feature maps to an output of the original dimensionality (128 × 128 × 3). The network was trained for 1,000 epochs to minimize mean absolute error between its input and output images (batch size 32, other learning parameters used the default Adam optimizer values as implemented in Keras).

#### Object-trained DNN

Finally, we evaluated a pretrained DNN supervised on object recognition: an 18-layer ResNet^57^ model available from the Deep Learning toolbox (https://mathworks.com/help/deeplearning/ref/resnet18.html) for MATLAB 2019b (The MathWorks Inc.). The network had been pretrained on the ImageNet Large-Scale Visual Recognition Challenge database to classify 1.2 million images into 1,000 object and animal categories^58^. For the analyses, we used representations in the 1,000-unit fully connected layer immediately before softmax category readout.

### Additional comparison models

#### Histogram skewness

Skewness was defined as the skew (third moment) of the distribution of pixel intensities in a greyscale version of each image.

#### MDS, tSNE and LLE

MDS is a linear dimensionality reduction technique that finds the linear projection of a dataset that best preserves the distances between all data points. tSNE^74^ is a nonlinear technique that preferentially preserves the distances between nearby data points. LLE^75^ is a nonlinear technique that seeks a lower-dimensional manifold that best preserves all distances.

Each dimensionality reduction algorithm was used to create a 10D embedding of the 10,000 images from the main training dataset, as well as all 270 rendered images used in the experiments (50 images from Experiment 1, 140 from Experiment 3 and 80 from Experiment 4). The experimental probe images were included because it is not possible to project new data points into the reduced-dimensionality solution discovered by tSNE or LLE. Additional 2D embeddings were performed to create the visualizations (Fig. 2a–c and Supplementary Fig. 2) using 4,000 images randomly selected from the main training dataset. The default parameters were used, as implemented in the scikit-learn package for Python.

#### Texture features

For each image, we calculated a multiscale texture feature description^85^ using the TextureSynth package for MATLAB (www.cns.nyu.edu/~lcv/texture/). The images were rescaled to 256 pixels × 256 pixels and analysed at three scales, four orientations, with a spatial neighbourhood of seven, producing a description of each image in terms of 1,350 feature dimensions.

### Psychophysical procedures

The psychophysical experiments were conducted in a dimly lit room, sitting at a comfortable distance from the screen. The participants could freely view the screen and were given no fixation instructions. The stimuli were presented on an EIZO ColorEdge CG277 self-calibrating LCD monitor with a resolution of 2,560 × 1,440 and a refresh rate of 60 Hz. PsychoPy v.3.1 (https://www.psychopy.org/) with Python v.3.6 was used to present the stimuli and record the responses. At the beginning of each experiment, the observers were shown 4–12 example experimental stimuli, randomly arranged, to familiarize them with the appearance and range of material properties that they would be asked to judge. Response times were unconstrained, and the stimuli were displayed until a response was recorded.

#### Experiment 1: gloss ratings

Experiment 1 measured gloss ratings for novel rendered images. Fifty 800 pixel × 800 pixel (18.6 cm × 18.6 cm) images were presented singly in the centre of the screen. Each was repeated three times, for a total of 150 trials, presented in a random order. The participants were asked to rate the apparent gloss of each image using a six-point scale with endpoints labelled ‘1 = completely matte’ and ‘6 = extremely glossy’.

#### Experiment 2: gloss manipulation

Experiment 2 measured gloss rankings for gloss-modulated network-generated images. Eighty sets of five 128 pixel × 128 pixel (2.9 cm × 2.9 cm) PixelVAE-generated images were shown. The five images in each set were arrayed in a random order in a row in the centre of the screen. The participants were asked to use the keyboard number keys to sort the images into a row of initially empty boxes at the bottom of the screen, in order from least glossy to most glossy. Each set of stimuli was shown once, for a total of 80 trials, presented in a random order.

#### Experiments 3a and 3b: patterns of gloss constancy

Experiment 3 measured pairwise gloss comparisons for probe sequences differing only in surface relief. Experiments 3a and 3b differed only in the specific sequences shown to the participants. Both experiments consisted of 10 sequences of 7 images, within which all possible pairs were shown twice each, for a total of 420 trials. The mages appeared at a resolution of 800 pixels × 800 pixels (18.6 cm × 18.6 cm), side by side on screen. Side of screen and trial order were randomized, with all sequences interleaved with one another. The participants were asked to report, using the left and right arrow keys, which surface appeared more glossy.

#### Experiment 4: degrees of gloss constancy

Experiment 4 measured pairwise gloss comparisons for pairs of images differing in surface relief or illumination. Each of 40 pairs of images was shown 8 times, for a total of 360 trials. The images appeared at a resolution of 128 pixels × 128 pixels (2.9 cm × 2.9 cm), side by side, with side of screen and trial order randomized. The participants reported, using the left and right arrow keys, which surface appeared more glossy.

### Data analysis

All analyses of human and model data were performed in Python v.2.7 or v.3.6, using the numpy v.1.16.5 and/or scikit-learn v.0.21.3 packages.

#### Representational similarity analysis

We measured the average correlation distance (1 minus Pearson’s *r*) between the latent representations of all 10,000 images in the main training dataset, grouped by whether they used the same (diagonal blocks) or different (off-diagonal blocks) gloss/lighting conditions, for each network training instance. Representational dissimilarity matrices (Fig. 2d,e) were created by averaging these values over the training instances of each model. To visualize the similarity of the model predictions (Fig. 7b), we created a vector, for each model, of predicted gloss values for all 270 rendered images used as experimental stimuli (50 images from Experiment 1, 140 images from Experiment 3 and 80 images from Experiment 4), normalized into the range 0–1. The Euclidean distance between these prediction vectors was calculated for all pairs of models.

#### Decoding world factors from models and deriving gloss predictions

For models with multidimensional feature spaces (that is, all except histogram skewness), a linear SVM was used to classify the specular reflectance (high versus low) of the rendered images from their representations within the model. The SVMs were trained on a random sample of 7,500 of the main training dataset images and tested on the remaining 2,500 to derive gloss classification accuracies (Fig. 3, Fig. 6a and Supplementary Figs. 4a and 5a). To derive continuously valued gloss predictions for the experimental images, the images were input to each model, and the signed distance of their representation from the model’s SVM decision boundary was measured. Positive values indicate high-gloss classifications, and negative values indicate low-gloss, with the absolute magnitude indicating the strength of evidence for that classification (Fig. 3, Fig. 4b,c, Fig. 5d,e, Fig. 7a, Fig. 8b and Supplementary Figs. 6b–d and 7b).

For the unsupervised PixelVAE and supervised ResNet models, we also trained linear SVMs to perform a six-way light-field classification, and we fitted linear regressions to predict surface relief (Fig. 3b). Classifiers and regressions were performed once using the full 10D latent space for each network and again using each of the network’s individual latent dimensions (Fig. 3b; see also Supplementary Fig. 1c for a visualization of the individual dimensions in one unsupervised network).

For the histogram skewness model, the raw skew values were used as gloss predictors for the purposes of model comparison. Ground-truth gloss classification accuracy (Fig. 6a) was defined as the accuracy using an optimal threshold to separate high-specular-reflectance from low-specular-reflectance images on the basis of their skewness, fitting the threshold on 7,500 main training dataset images and testing on the remaining 2,500.

#### Deriving predicted 2AFC experimental data from models

The model predictions for Experiment 3 were derived by simulating responses of each model in the 2AFC task performed by humans. For each sequence of seven images, the predicted gloss values of each possible pair of images were compared, and the image for which the model predicted higher gloss was selected. The predicted responses were summarized as ‘proportion selected as being glossier’ for each level in the sequence, as for the human data (Fig. 5b and Supplementary Fig. 3).

#### Measuring highlight features

Coverage, sharpness and contrast of highlights (Fig. 8) were measured using a MATLAB package developed by Schmid et al.^115^. Briefly, coverage is defined as the proportion of pixels with higher intensities in the full image than in the diffuse component image, sharpness is defined as the local phase coherence^116^ within the specular component image and contrast is defined as the sum of the root mean square contrast across eight bandpass-filtered versions of the specular component image.

### Psychophysical data analysis

No participants or trials were excluded from the analysis. In Experiment 1, both human ratings and model-predicted gloss values were normalized to the range 0–1 before comparing, individually for each participant or model instance. In Experiment 2, the rankings were converted to average rank position of each image step for each participant, averaging within matte and glossy seed image sequences. In Experiments 3a and 3b, the pairwise comparisons were converted to the proportion of times each image in each sequence was judged as having higher gloss, across pairings and repetitions, for each participant. In Experiment 4, the pairwise comparisons were converted to the proportion of times that image A (arbitrarily labelled) was judged as having higher gloss than image B, and this proportion was subtracted from 0.5 (that is, 0 indicates equal apparent gloss and good constancy; deviations in either direction indicate deviations from constancy). The model predictions were obtained by subtracting the predicted gloss value of image B from that of image A and scaling this gloss difference into the range −0.5 to 0.5, retaining the original zero point. For models with multiple training instances, model predictions were always derived (and performances calculated) for each individual training instance.

### Statistical analysis

The statistical analyses were performed using the Pingouin^117^ package for Python (v.0.3.10). All tests were two-tailed. The CIs reported for mean differences and correlations were calculated by bootstrapping the respective estimate 10,000 times. The data distributions were assumed to be normal, but this was not formally tested. The gloss predictions of all models were fixed, with no free parameters in evaluation against human data, except for the analysis of the data in Fig. 5e, where the performance of a logistic transform of unsupervised model predictions is also reported.

### Reporting Summary

Further information on research design is available in the Nature Research Reporting Summary linked to this article.

## Supplementary information

Supplementary Information Supplementary Methods, Supplementary Results, Supplementary Table 1 and Supplementary Figs. 1–7.

Reporting Summary

Peer Review Information

## Data Availability

All human and model data are available on Zenodo at 10.5281/zenodo.4495586.

## Code availability

The custom analysis code that supports the findings of this study is available on Zenodo at 10.5281/zenodo.4495586.

## References

[1] Adelson, E. H. Lightness perception and lightness illusions. in *The New Cognitive Neurosciences* (ed. Gazzaniga, M.S.) 339–351 (MIT Press, 2000).

[2] Anderson BL (2020). Mid-level vision. Curr. Biol..

[3] Anderson, B. L. The perceptual representation of transparency, lightness, and gloss. in *Handbook of Perceptual Organization* (ed. Wagemans, J.) 466–483 (Oxford University Press, 2015).

[4] Barrow H, Tenenbaum J, Hanson A, Riseman E (1978). Recovering intrinsic scene characteristics. Comput. Vis. Syst..

[5] Fleming RW (2017). Material perception. Annu. Rev. Vis. Sci..

[6] Todd JT (2004). The visual perception of 3D shape. Trends Cogn. Sci..

[7] Todd JT, Norman JF, Mingolla E (2004). Lightness constancy in the presence of specular highlights. Psychol. Sci..

[8] Marr, D. *Vision* (Freeman, 1982).

[9] Kersten D, Mamassian P, Yuille A (2004). Object perception as Bayesian inference. Annu. Rev. Psychol..

[10] Geisler WS, Kersten D (2002). Illusions, perception and Bayes. Nat. Neurosci..

[11] von Helmholtz, H. *Handbuch der physiologischen Optik* Vol. 3 (1867). English edition: *Treatise on Physiological Optics* Vol. 3 (trans. Ladd-Franklin, C., Gullstrand, A. and von Kries, J.) (Courier Corporation, 2013).

[12] Anderson BL (2015). Can computational goals inform theories of vision?. Top. Cogn. Sci..

[13] Hoffman DD, Singh M, Prakash C (2015). The interface theory of perception. Psychon. Bull. Rev..

[14] Fleming RW, Storrs KR (2019). Learning to see stuff. Curr. Opin. Behav. Sci..

[15] Barlow H (2001). The exploitation of regularities in the environment by the brain. Behav. Brain Sci..

[16] DiCarlo JJ, Cox DD (2007). Untangling invariant object recognition. Trends Cogn. Sci..

[17] Storrs, K. R. & Fleming, R. W. Learning about the world by learning about images. *Curr. Dir. Psychol. Sci.* (in the press).

[18] Higgins, I. et al. Towards a definition of disentangled representations. Preprint at *arXiv* https://arxiv.org/abs/1812.02230 (2018).

[19] Barlow HB (1961). Possible principles underlying the transformation of sensory messages. Sens. Commun..

[20] Attneave F (1954). Some informational aspects of visual perception. Psychol. Rev..

[21] Simoncelli EP, Olshausen BA (2001). Natural image statistics and neural representation. Annu. Rev. Neurosci..

[22] Olshausen BA, Field DJ (1996). Emergence of simple-cell receptive field properties by learning a sparse code for natural images. Nature.

[23] Grossberg S (1976). Adaptive pattern classification and universal recoding: I. Parallel development and coding of neural feature detectors. Biol. Cybern..

[24] Földiak P (1990). Forming sparse representations by local anti-Hebbian learning. Biol. Cybern..

[25] Anderson BL (2011). Visual perception of materials and surfaces. Curr. Biol..

[26] Gilchrist A (1999). An anchoring theory of lightness perception. Psychol. Rev..

[27] Pont SC, te Pas SF (2006). Material—illumination ambiguities and the perception of solid objects. Perception.

[28] Adams WJ, Kucukoglu G, Landy MS, Mantiuk RK (2018). Naturally glossy: gloss perception, illumination statistics, and tone mapping. J. Vis..

[29] Foster DH (2011). Color constancy. Vis. Res..

[30] Motoyoshi I, Matoba H (2012). Variability in constancy of the perceived surface reflectance across different illumination statistics. Vis. Res..

[31] Chadwick AC, Kentridge R (2015). The perception of gloss: a review. Vis. Res..

[32] Obein G, Knoblauch K, Viéot F (2004). Difference scaling of gloss: nonlinearity, binocularity, and constancy. J. Vis..

[33] Fleming RW, Dror RO, Adelson EH (2003). Real-world illumination and the perception of surface reflectance properties. J. Vis..

[34] Ho Y-X, Landy MS, Maloney LT (2008). Conjoint measurement of gloss and surface texture. Psychol. Sci..

[35] Marlow PJ, Kim J, Anderson BL (2012). The perception and misperception of specular surface reflectance. Curr. Biol..

[36] Doerschner K (2011). Visual motion and the perception of surface material. Curr. Biol..

[37] Wendt G, Faul F, Ekroll V, Mausfeld R (2010). Disparity, motion, and color information improve gloss constancy performance. J. Vis..

[38] Toscani M, Guarnera D, Guarnera C, Hardeberg JY, Gegenfurtner K (2020). Three perceptual dimensions for specular and diffuse reflection. ACM Trans. Appl. Percept..

[39] Ferwerda JA, Pellacini F, Greenberg DP (2001). Psychophysically based model of surface gloss perception. Proc. SPIE Int. Soc. Opt. Eng..

[40] Lagunas M (2019). A similarity measure for material appearance. ACM Trans. Graph..

[41] Ingersoll LR (1921). The glarimeteran instrument for measuring the gloss of paper. J. Opt. Soc. Am..

[42] Ward, G. J. Measuring and modeling anisotropic reflection. In *Proc. 19th Annual Conference on Computer Graphics and Interactive Techniques* (ed. Thomas, J.J.) 265–272 (Association for Computing Machinery, 1992).

[43] Wills J, Agarwal S, Kriegman D, Belongie S (2009). Toward a perceptual space for gloss. ACM Trans. Graph..

[44] Serrano, A., Gutierrez, D., Myszkowski, K., Seidel, H.-P. & Masia, B. An intuitive control space for material appearance. *ACM Trans. Graph.* **35** (2016).

[45] Vangorp, P., Laurijssen, J. & Dutré, P. The influence of shape on the perception of material reflectance. *ACM SIGGRAPH 2007* **77** (2007).

[46] Salakhutdinov R (2015). Learning deep generative models. Annu. Rev. Stat. Appl..

[47] Zhao, S., Song, J. & Ermon, S. Towards deeper understanding of variational autoencoding models. Preprint at *arXiv* https://arxiv.org/abs/1702.08658 (2017).

[48] Gulrajani, I. et al. PixelVAE: a latent variable model for natural images. Preprint at *arXiv* https://arxiv.org/abs/1611.05013 (2016).

[49] Radford, A., Metz, L. & Chintala, S. Unsupervised representation learning with deep convolutional generative adversarial networks. Preprint at *arXiv* https://arxiv.org/abs/1511.06434 (2015).

[50] Higgins, I. et al. beta-VAE: learning basic visual concepts with a constrained variational framework. in *5th International Conference on Learning Representations (ICLR)* https://openreview.net/pdf?id=Sy2fzU9gl (2017).

[51] Lindsay, G. Convolutional neural networks as a model of the visual system: past, present, and future. *J. Cogn. Neurosci.*10.1162/jocn_a_01544 (2020). 10.1162/jocn_a_01544 32027584

[52] Yamins DL, DiCarlo JJ (2016). Using goal-driven deep learning models to understand sensory cortex. Nat. Neurosci..

[53] Storrs, K. R. & Kriegeskorte, N. Deep learning for cognitive neuroscience. in *The Cognitive Neurosciences* (eds. Poeppel, D., Mangun, G. R., & Gazzaniga, M. S.) 703–716 (MIT Press, 2020).

[54] Richards BA (2019). A deep learning framework for neuroscience. Nat. Neurosci..

[55] Kriegeskorte N (2015). Deep neural networks: a new framework for modeling biological vision and brain information processing. Annu. Rev. Vis. Sci..

[56] LeCun Y, Bengio Y, Hinton G (2015). Deep learning. Nature.

[57] He, K., Zhang, X., Ren, S. & Sun, J. Deep residual learning for image recognition. In *Proc. IEEE Conference on Computer Vision and Pattern Recognition* 770–778 (2016).

[58] Russakovsky O (2015). ImageNet Large Scale Visual Recognition Challenge. Int. J. Comput. Vis..

[59] Taigman, Y., Yang, M., Ranzato, M. & Wolf, L. Deepface: closing the gap to human-level performance in face verification. In *Proc. IEEE Conference on Computer Vision and Pattern Recognition* 1701–1708 (2014).

[60] Cadieu CF (2014). Deep neural networks rival the representation of primate IT cortex for core visual object recognition. PLoS Comput. Biol..

[61] Schrimpf, M. et al. Brain-score: which artificial neural network for object recognition is most brain-like? Preprint at *bioRxiv*10.1101/407007 (2018).

[62] Storrs, K. R., Kietzmann, T. C., Walther, A., Mehrer, J. & Kriegeskorte, N. Diverse deep neural networks all predict human IT well, after training and fitting. *J. Cogn. Neurosci.* (in the press). 10.1162/jocn_a_01755 34272948

[63] Khaligh-Razavi, S.-M. & Kriegeskorte, N. Deep supervised, but not unsupervised, models may explain IT cortical representation. *PLoS Comput. Biol.* **10** (2014). 10.1371/journal.pcbi.1003915 PMC4222664 25375136

[64] Nguyen, A., Yosinski, J. & Clune, J. Deep neural networks are easily fooled: high confidence predictions for unrecognizable images. in *Proc. IEEE Conference on Computer Vision and Pattern Recognition* 427–436 (2015).

[65] Geirhos R (2018). Generalisation in humans and deep neural networks. Adv. Neural Inf. Process. Syst..

[66] Geirhos, R. et al. ImageNet-trained CNNs are biased towards texture; increasing shape bias improves accuracy and robustness. Preprint at *arXiv* https://arxiv.org/abs/1811.12231 (2018).

[67] Geirhos R (2020). Shortcut learning in deep neural networks. Nature Machine Intelligence.

[68] Kingma, D. P. & Welling, M. Auto-encoding variational Bayes. in *International Conference on Learning Representations 2013* https://openreview.net/forum?id=33X9fd2-9FyZd (2013).

[69] Hinton GE, Salakhutdinov RR (2006). Reducing the dimensionality of data with neural networks. Science.

[70] Mehrer, J., Spoerer, C. J., Kriegeskorte, N. & Kietzmann, T. C. Individual differences among deep neural network models. *Nat. Commun*. **11** (2020). 10.1038/s41467-020-19632-w PMC7665054 33184286

[71] He, K., Zhang, X., Ren, S. & Sun, J. Delving deep into rectifiers: surpassing human-level performance on ImageNet classification. In *Proc. IEEE International Conference on Computer Vision* 1026–1034 (2015).

[72] Bengio Y, Courville A, Vincent P (2013). Representation learning: a review and new perspectives. IEEE Trans. Pattern Anal. Mach. Intell..

[73] Testolin A, Stoianov I, Zorzi M (2017). Letter perception emerges from unsupervised deep learning and recycling of natural image features. Nat. Hum. Behav..

[74] van der Maaten L, Hinton G (2008). Visualizing data using t-SNE. J. Mach. Learn. Res..

[75] Roweis ST, Saul LK (2000). Nonlinear dimensionality reduction by locally linear embedding. Science.

[76] Nili H (2014). A toolbox for representational similarity analysis. PLoS Comput. Biol..

[77] Kriegeskorte N, Kievit RA (2013). Representational geometry: integrating cognition, computation, and the brain. Trends Cogn. Sci..

[78] Kriegeskorte N, Diedrichsen J (2016). Inferring brain-computational mechanisms with models of activity measurements. Phil. Trans. R. Soc. B.

[79] Testolin A, Zorzi M (2016). Probabilistic models and generative neural networks: towards an unified framework for modeling normal and impaired neurocognitive functions. Front. Comput. Neurosci..

[80] Hong H, Yamins DL, Majaj NJ, DiCarlo JJ (2016). Explicit information for category-orthogonal object properties increases along the ventral stream. Nat. Neurosci..

[81] Naselaris T, Kay KN, Nishimoto S, Gallant JL (2011). Encoding and decoding in fMRI. NeuroImage.

[82] Gatys L, Ecker AS, Bethge M (2015). Texture synthesis using convolutional neural networks. Adv. Neural Inf. Process. Syst..

[83] Zhang, R., Isola, P., Efros, A. A., Shechtman, E. & Wang, O. The unreasonable effectiveness of deep features as a perceptual metric. In *Proc. IEEE Conference on Computer Vision and Pattern Recognition* 586–595 (2018).

[84] Rajalingham R (2018). Large-scale, high-resolution comparison of the core visual object recognition behavior of humans, monkeys, and state-of-the-art deep artificial neural networks. J. Neurosci..

[85] Portilla J, Simoncelli EP (2000). A parametric texture model based on joint statistics of complex wavelet coefficients. Int. J. Comput. Vis..

[86] Motoyoshi I, Nishida S, Sharan L, Adelson EH (2007). Image statistics and the perception of surface qualities. Nature.

[87] Funke CM (2021). Five points to check when comparing visual perception in humans and machines. J. Vis..

[88] Golan T, Raju PC, Kriegeskorte N (2020). Controversial stimuli: pitting neural networks against each other as models of human cognition. Proc. Natl Acad. Sci. USA.

[89] Wang Z, Simoncelli EP (2008). Maximum differentiation (MAD) competition: a methodology for comparing computational models of perceptual quantities. J. Vis..

[90] Havran V, Filip J, Myszkowski K (2016). Perceptually motivated BRDF comparison using single image. Comput. Graph. Forum.

[91] Wiebel CB, Valsecchi M, Gegenfurtner KR (2013). The speed and accuracy of material recognition in natural images. Atten. Percept. Psychophys..

[92] Beck J, Prazdny S (1981). Highlights and the perception of glossiness. Percept. Psychophys..

[93] Anderson BL, Kim J (2009). Image statistics do not explain the perception of gloss and lightness. J. Vis..

[94] Marlow PJ, Todorović D, Anderson BL (2015). Coupled computations of three-dimensional shape and material. Curr. Biol..

[95] Marlow PJ, Anderson BL (2015). Material properties derived from three-dimensional shape representations. Vis. Res..

[96] Marlow PJ, Anderson BL (2013). Generative constraints on image cues for perceived gloss. J. Vis..

[97] Simoncelli EP (2003). Vision and the statistics of the visual environment. Curr. Opin. Neurobiol..

[98] Sawayama M, Nishida S (2018). Material and shape perception based on two types of intensity gradient information. PLoS Comput. Biol..

[99] Nishida S, Shinya M (1998). Use of image-based information in judgments of surface-reflectance properties. J. Opt. Soc. Am. A.

[100] Adelson, E. H. & Pentland, A. P. in *Perception as Bayesian Inference* (eds Knill, D. S. & Richards, W.) 409–423 (Cambridge Univ. Press, 1996).

[101] Marlow PJ, Anderson BL (2016). Motion and texture shape cues modulate perceived material properties. J. Vis..

[102] Wiesel TN, Hubel DH (1974). Ordered arrangement of orientation columns in monkeys lacking visual experience. J. Comp. Neurol..

[103] Yang J, Otsuka Y, Kanazawa S, Yamaguchi MK, Motoyoshi I (2011). Perception of surface glossiness by infants aged 5 to 8 months. Perception.

[104] Balas B (2017). Children’s use of visual summary statistics for material categorization. J. Vis..

[105] Balas B, Auen A, Thrash J, Lammers S (2020). Children’s use of local and global visual features for material perception. J. Vis..

[106] Smith LB, Slone LK (2017). A developmental approach to machine learning?. Front. Psychol..

[107] Pouget A, Beck JM, Ma WJ, Latham PE (2013). Probabilistic brains: knowns and unknowns. Nat. Neurosci..

[108] Friston K (2009). The free-energy principle: a rough guide to the brain?. Trends Cogn. Sci..

[109] Deneve S (2008). Bayesian spiking neurons I: inference. Neural Comput..

[110] Brainard DH (2000). Functional consequences of the relative numbers of L and M cones. J. Opt. Soc. Am. A.

[111] Smirnakis SM, Berry MJ, Warland DK, Bialek W, Meister M (1997). Adaptation of retinal processing to image contrast and spatial scale. Nature.

[112] Fleming RW (2014). Visual perception of materials and their properties. Vis. Res..

[113] Salimans, T., Karpathy, A., Chen, X. & Kingma, D. P. PixelCNN++: improving the PixelCNN with discretized logistic mixture likelihood and other modifications. Preprint at *arXiv* https://arxiv.org/abs/1701.05517 (2017).

[114] Van den Oord A (2016). Conditional image generation with PixelCNN decoders. Adv. Neural Inf. Process. Syst..

[115] Schmid, A. C., Barla, P. & Doerschner, K. Material category determined by specular reflection structure mediates the processing of image features for perceived gloss. Preprint at *bioRxiv*10.1101/2019.12.31.892083 (2020).

[116] Hassen R, Wang Z, Salama MMA (2013). Image sharpness assessment based on local phase coherence. IEEE Trans. Image Process..

[117] Vallat R (2018). Pingouin: statistics in Python. J. Open Source Softw..

