# Supplementary material for: Unsupervised learning predicts human perception and misperception of gloss
Source: Nat Hum Behav. 2021 May 6;5(10):1402–17. doi: 10.1038/s41562-021-01097-6 (PMC8526360; doi:10.1038/s41562-021-01097-6)
Supplement: Supplementary file 1 — Supplementary Methods, Supplementary Results, Supplementary Table 1 and Supplementary Figs. 1–7. [file 41562_2021_1097_MOESM1_ESM.pdf]

nature portfolio

https://doi.org/10.1038/s41562-021-01097-6

# Supplementary information

# Unsupervised learning predicts human perception and misperception of gloss

In the format provided by the
authors and unedited

# Unsupervised learning predicts human perception and misperception of gloss

Katherine R. Storrs, Barton L. Anderson & Roland W. Fleming

## Supplementary Information

# Supplementary Methods and Results

***Pilot experiment to select latent dimensionality of unsupervised network models.*** We wanted to select a latent dimensionality for the PixelVAE model such that the network was forced to learn a highly compressed code, but still able to capture the majority of meaningful structure in the training images. To gauge where this dimensionality lay for our dataset, we trained five PixelVAE networks with latent dimensionalities of 2, 5, 10, 20 and 40. From each network we generated 50 images by conditioning the generative sampling process on each of 50 random bumpy surface images that did not belong to the training dataset (see **Supplementary Figure 1A**). We then performed a psychophysical pilot experiment in which we showed twenty observers the generated images. On a trial, one of the rendered seed images was shown at the top of the screen, and its corresponding five generated images (one from each network) were shown in a random order in a horizontal row beneath. Participants rearranged the five generated images in order of their apparent similarity to the rendered seed image, and we measured the average similarity ranking given to the images generated by each model. Results are shown in **Supplementary Figure 1B**. Apparent similarity of generated images to seed images increased with a model's latent dimensionality, but began to plateau after a dimensionality of ten, which we used for all subsequent PixelVAE models.

***Performance of intermediate layers in models.*** The analyses in the main work focus only on one 10-dimensional layer within both the supervised and unsupervised models. We also explored how well representations in the intermediate layers could predict both ground-truth gloss and human gloss perception, for one training instance of each model type. From the unsupervised PixelVAE architecture we trained an SVM to categorise high-vs-low gloss surfaces from the activations in each of the three convolutional layers of the autoencoder stream leading up to the latent code. From the supervised Resnet architecture we trained an SVM to categorise gloss from activations in the last convolutional layer of each each of the three residual blocks leading up to the output classification. Gloss classification accuracy was near-perfect for all layers of the supervised network, but increased across layers in the unsupervised network. Using the trained gloss classifiers, we then calculated predicted gloss values for each layer of each model and compared them to human gloss judgements in three psychophysical experiments as for all models in the main manuscript. The unsupervised network better predicted human gloss judgements across all layers, gradually improving across convolutional layers towards the 10-dimensional latent code (see **Supplementary Figure 4A**).

***Continuous gloss regression supervised network model.*** To explore whether a supervised network with a richer training objective might learn representations more similar to humans', we created a version of the ResNet supervised model architecture in which the final two "high-vs-low-gloss" categorical units were replaced with a single continuously-valued "gloss level" unit (see **Supplementary Figure 4B**). A new training set of 10,000 images was rendered, in which the magnitude of the specular component was sampled randomly uniformly between 0 and 1, and the concentration of the specular component was set to the same value. All other scene factors were varied as in the original training set. This created a continuously-sampled one-dimensional gloss space, ranging from completely matte to completely mirrored. The supervised regression model was trained for 21 epochs on 9,000 of the images to predict the true specular magnitude, by minimising mean absolute error (MAE) using the Adam optimiser with a learning rate of 0.0001 and a decay rate of 0.9999 after each epoch. Other network parameters were kept as for the categorisation-supervised models. Five model instances were trained from different random initial weight settings. Ability to predict specular magnitude was excellent (average MAE on 500 test images = 0.002). We then evaluated the model's ability to predict human data in the same way as for the categorisation-supervised ResNet models in the main manuscript, by training a gloss-classification SVM on the feature space in the 10D penultimate layer of the network, deriving gloss predictions for experimental images, and comparing them to human gloss judgements. (see **Supplementary Figure 4B**). The regression-supervised networks performed better than the categorisation-supervised networks, predicting human judgements on average as well as ground-truth specular reflectance (one-sample t-test of model RMSE against ground truth RMSE across model training instances $t_4 = 0.24$, $p = 0.82$, Cohen's d = 0.10, 95% CI of difference = [-0.02–0.02]). Unsupervised models, however, consistently outperform ground-truth, predicting *errors* in human gloss perception, as well as successes (one-sample t-test of the poorest-performing set of unsupervised networks—those trained on a continuously-varying gloss dataset**—**against ground truth $t_4 = -4.78$, $p = 0.009$, d = 2.14, 95% CI = [0.02–0.08]; independent-samples t-test against regression-supervised networks $t_8 = 3.98$, $p = 0.005$, d = 2.52, 95% CI = [0.02–0.08]).

***Effect of varying model hyperparameters.*** When working with deep neural networks one must choose values for a large number of hyperparameters, such as the number of layers and convolutional filters, the learning rate, the rate at which the learning rate decays as training progresses, and more. Fully exploring this hyperparameter space is prohibitively time consuming, so we performed a small exploration of some key factors by training 28 additional models (14 unsupervised PixelVAE and 14

supervised ResNet networks) and evaluating their ability to classify gloss and to predict human gloss perception (see **Supplementary Figure 5**). **Supplementary Table 1** lists the variants tested. For both the unsupervised and supervised models we tested a deeper and shallower architecture than the one originally used, as well as lower and higher learning rates, and lower and higher decay rates for the learning rate. Some hyperparameters of interest could not be varied in both model types. For batch size, we explored higher and lower sizes for the supervised model only, as memory constraints on the training GPUs limited the unsupervised model to the small batch size of 5. Finally, we explored higher and lower degrees of complexity for the pixel distribution learned by the unsupervised PixelVAE model, a hyperparameter not present in the supervised model. For each variant, we altered only the single hyperparameter of interest, and held all others at their original values. All models were trained for the same duration as the original models (200 epochs for PixelVAE models and 25 epochs for ResNet models, which was sufficient for convergence in each model type, see **Supplementary Figure 5B**). Once trained, each model instance was evaluated against human psychophysical data from Experiments 1, 3 and 4 identically to the model evaluation procedures in the main manuscript. Average error in predicting human data, across the three experiments, is shown for each network in **Supplementary Figure 5A** and **C**.

Unsupervised models generally predicted human perceptual data better than supervised models across the wide range of hyperparameter settings explored, with the exception of two unsupervised models with large learning rates that failed to train (as evidenced by poor convergence and noisy generated samples). The performances of the original unsupervised model training instances fall towards the better end of the range of performances found for hyperparameter variants, and those of the original supervised instances fall towards the worse end of the range of performances found for variants, but both are within the bounds apparently typical of their model types. The slightly larger difference between the original models may be due to having specifically selected stimuli for Experiments 3 and 4 in order to maximise the “disagreement” in gloss predictions made by the original models.

| Hyperparameter | Values tested in unsupervised PixelVAE models | Values tested in supervised ResNet models | Value in original unsupervised PixelVAE model | Value in original supervised ResNet model |
|---|---|---|---|---|
| Depth of network | 2 three-layer blocks, with 80 convolutional feature maps per layer<br>4 three-layer blocks, with 48 convolutional feature maps per layer | 2 three-layer blocks, with 80 convolutional feature maps per layer<br>4 three-layer blocks, with 48 convolutional feature maps per layer | 3 three-layer blocks, with 64 convolutional feature maps per layer (in encoder part of PixelCNN stream) | 3 three-layer blocks, with 56 convolutional feature maps per layer |
| Learning rate | 0.01<br>0.005<br>0.0005<br>0.0001 | 0.01<br>0.005<br>0.0005<br>0.0001 | 0.001 | 0.001 |
| Decay factor for learning rate | 0.25<br>0.50<br>2.00<br>4.00 | 0.25<br>0.50<br>2.00<br>4.00 | 1.0 | 1.0 |
| Batch size | | 12<br>22<br>42<br>50 | 5 (at memory capacity) | 32 |
| Complexity of PixelVAE's learned pixel distribution | 4 logistic functions<br>6 logistic functions<br>24 logistic functions<br>30 logistic functions | | 12 logistic functions | n/a |

**Supplementary Table 1**: Hyperparameter values for each of 28 alternative variant models. For each variant, only the specified hyperparameter was altered, and all others were kept at the values used in the original models. The learning rate decay factor was used to gradually reduce the learning rate during training by multiplying the learning rate after each epoch by $1 - decay\ factor\left(learning\ rate/total\ epochs\right)$.The PixelVAE model a pixel probability distribution over RGB space, which is parameterised as a mixture of logistic functions. The number of logistic functions is a hyperparameter which controls the complexity of the learned distribution.

***Tolerance to variations in rendering method.*** In order to generate large datasets in a reasonable timeframe, we used real-time rasterised rendering in the Unity3D engine. The resulting images have

good visual quality, but are not perfectly physically faithful. For example, they lack inter-reflections, and use an approximation of ambient occlusion. To test whether the unsupervised models trained on these rasterised images could make reasonable gloss predictions for surfaces rendered with more time-intensive but physically accurate methods, we generated 24 images of surfaces rendered once using rasterisation, and once physically faithful ray-tracing, via the Eevee and Cycles renders within the Blender engine, respectively. Each image was then input to all training instances of the original unsupervised PixelVAE model, and an average predicted gloss value was calculated. The models' gloss classifications were correct for all images rendered via both methods, although gloss values were systematically slightly higher for images rendered via the rasterised method familiar to the networks (see **Supplementary Figure 6B**).

***Generalisation to real-world photographs.*** We performed two tests of generalisation to real-world images. First, we took 20 colour close-up photographs of common surfaces, ten of which were highly glossy (e.g. tomatoes, plastic, metallic foil) and ten of which were strongly matte (e.g. chalk, fleece, styrofoam). Predicted gloss values for each image were calculated for each of the ten training instances of the original unsupervised PixelVAE models (**Supplementary Figure 6C**). The unsupervised models were able to correctly classify all but one of the surfaces as being high or low gloss. Second, we performed a broader test by inputting to the models all images from the Giessen Material Image Database (Wiebel, Valsecchi & Gegenfurtner, 2013), which comprises 300 close-up photographs of assorted wood, metal, stone, and fabric surfaces. Predicted gloss values for each image from each model training instance are shown in **Supplementary Figure 6D**. Although the unsupervised model was moderately successful at sorting the "metal" category images from dull brushed surfaces to highly polished ones, it also showed clear failure cases. For example, matte fabrics with high-contrast patterns often received high predicted gloss values. Given that textured or patterned surfaces never occurred in the model's training dataset, we would not expect it to have been able to learn the image structures associated with these.

# Supplementary Figures

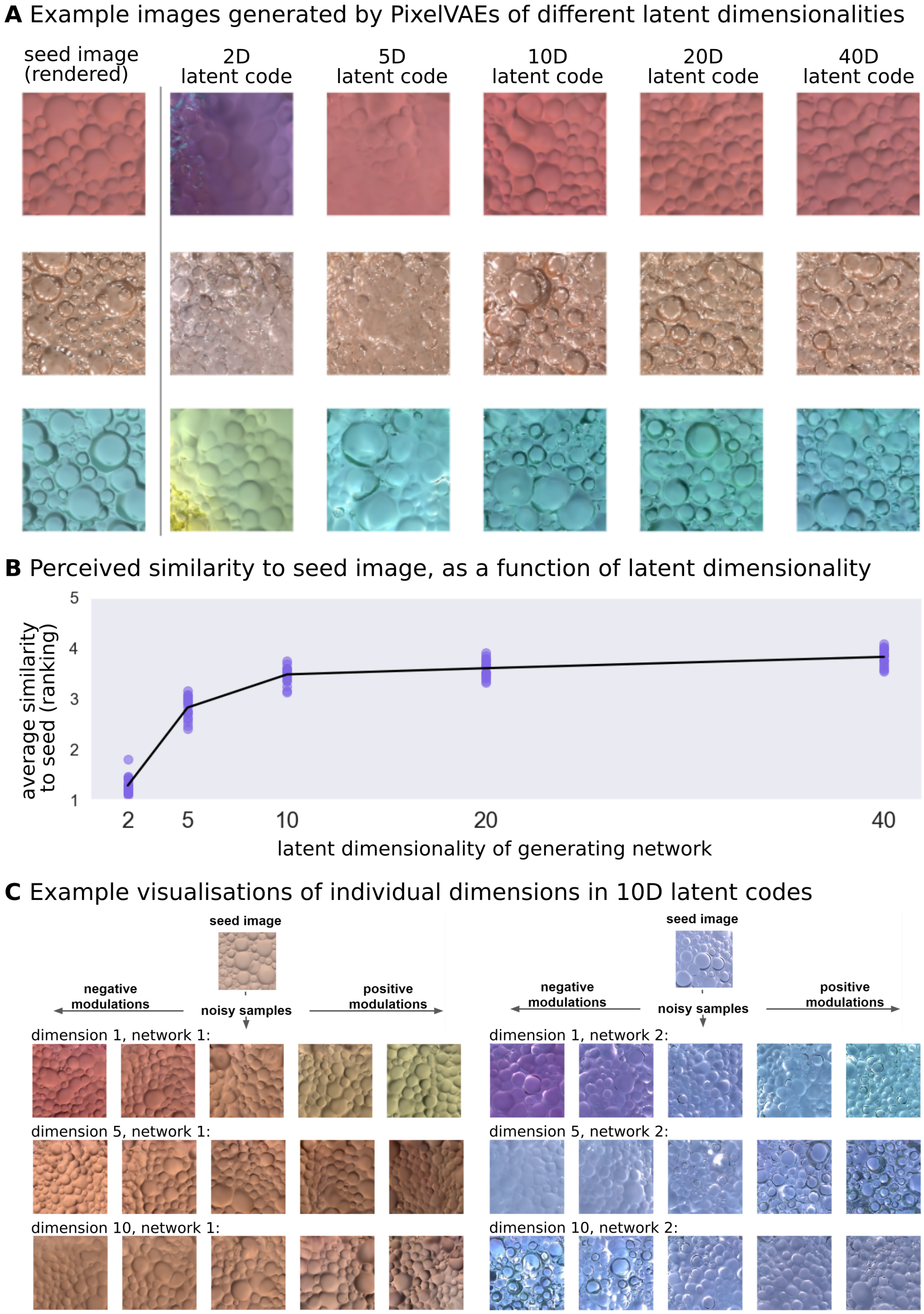

**Supplementary Figure 1 [previous page]: Choice of latent dimensionality, and visualisation of single dimensions.**

**(A)** Examples of images generated by networks with latent layers containing 2, 5, 10, 20, and 40 dimensions, using each of the three images in the leftmost column as "seeds" to select a point to sample in the latent space. Sample quality improves as latent dimensionality increases, up to a point.

**(B)** Mean ranking of samples (y-axis) from each network (x-axis) in terms of perceived similarity to seed images, for each observer (coloured dots) in a psychophysical pilot experiment. Line indicates mean over all observers. Above around ten latent dimensions, there are diminishing perceptual quality returns when adding further dimensions, for this image training set.

**(C)** Example visualisations of three individual dimensions within two different training instances of models with 10 latent dimensions, for two seed images. The central column beneath each seed image shows different samples generated by the network when sampled on the identical point in latent space. To generate the images in the two columns to the left of this, we conditioned on two points slightly negatively shifted, along only the stated dimension; to generate the images in the two right columns, we conditioned on two points slightly positively shifted along the same dimension. Individual dimensions capture variations in shape, colour, material, and lighting, and often combine multiple of these properties (i.e. the representation of world factors is distributed, not sparsely encoded by single dimensions).

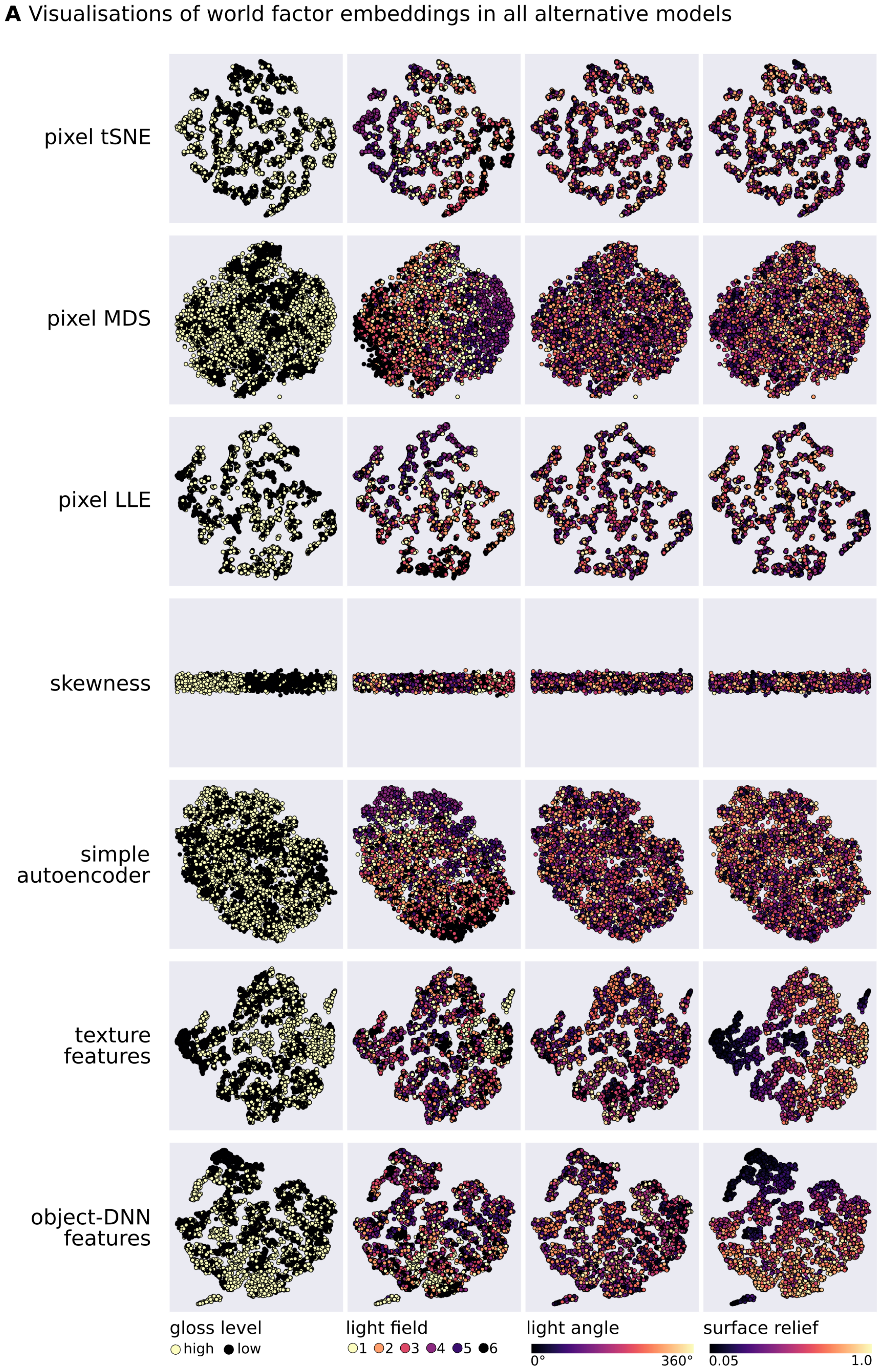
A Visualisations of world factor embeddings in all alternative models
pixel tSNE
pixel MDS
pixel LLE
skewness
simple autoencoder
texture features
object-DNN features
gloss level
high
low
light field
1
2
3
4
5
6
light angle
0°
360°
surface relief
0.05
1.0

**Supplementary Figure 2 [previous page]: Visualisations of how world factors are embedded within the feature spaces of all alternative models.**

**(A)** Shown are visualisations of 4,000 random images from the training dataset, projected into two dimensions via t-weighted stochastic neighbourhood embedding for all models not shown in Figure 2 of the main manuscript. For each model, the 2D embedding is coloured by (from left to right) gloss level of the surface, light field illuminating the scene, rotation of light field with respect to the surface, and depth of surface relief. The luminance histogram skewness model (fourth row) is a one-dimensional feature space; some random vertical jitter has been added for easier visualisation. Most world factors are thoroughly intermingled in most of these alternative models, with a few exceptions. High and low gloss images are reasonably well separated by the simple statistic of luminance histogram skewness, within this dataset of cleanly bimodal gloss. Illumination fields are roughly separated within the pixel MDS and simple autoencoder feature spaces, likely because they influence the colour distributions in images. Interestingly, surface relief is best represented in the models consisting of texture features or features from a DNN trained to recognise objects in natural images, which also predict human judgements best among the alternative models.

**A** Human data and model predictions for each test sequence

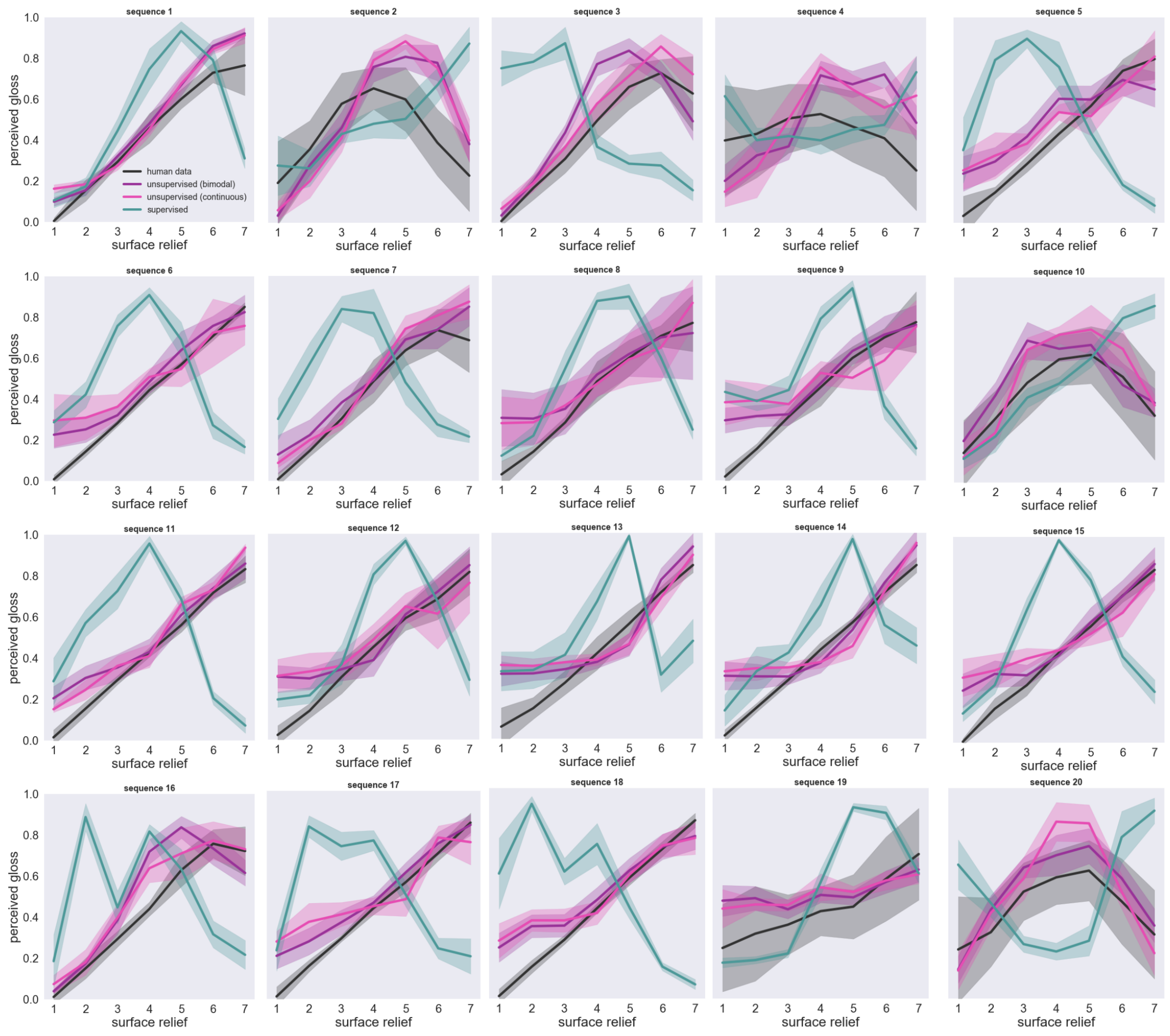


**Supplementary Figure 3: Full data for Experiment 3**

**(A)** Full data from each of the twenty constancy sequences summarised in **Figure 5B-C** of the main text, including predictions from a new set of five PixelVAE models trained on an independent dataset in which gloss varies continuously rather than bimodally ("PixelVAE continuous-gloss" model referred to in main text and figures).

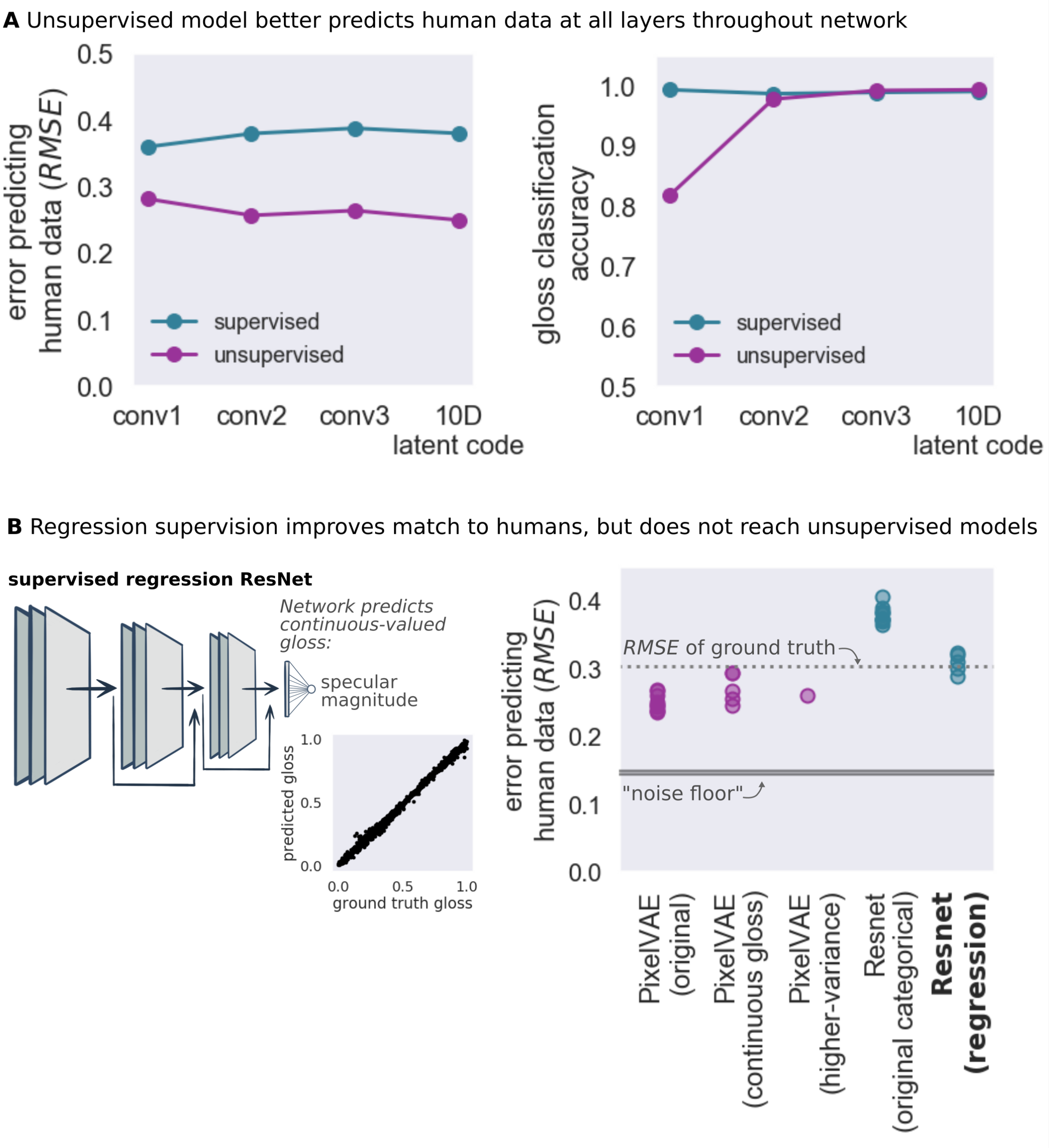


**Supplementary Figure 4: Main results are robust to changes in layer chosen for readout or training objective of supervised network.**

**(A)** Average error in predicting human gloss judgements across three psychophysical experiments (left) and objective gloss-classification accuracy (right) for successive convolutional layers of the unsupervised (purple) and supervised (teal) models, for one model training instance of each. The rightmost data points in each plot show the performance of the 10-dimensional latent code layer that forms the basis of all other analyses. The unsupervised model consistently better predicts human data across all its layers, whereas the supervised model displays near-perfect objective gloss classification throughout its layers.

**(B)** Schematic (left) of the ResNet architecture adapted to output a single scalar value. After supervised training to predict specular magnitude, the model approximates ground-truth well (inset scatterplot shows true and predicted gloss levels for 500 unseen test images for one training instance of the network). Plot (right) shows average error in predicting human gloss judgements, across three psychophysical experiments, for the five training instances of the regression-supervised ResNet model (rightmost column). For comparison, performance is also shown for each training instance of (from left to right) the original unsupervised model, an unsupervised model trained on a dataset in which specular magnitude and concentration vary randomly and independently, an unsupervised model trained on a dataset in which surface geometry and illumination vary more widely, and for the original categorisation-supervised Resnet model. For reference, the dotted grey line indicates how well human judgements can be predicted by ground truth specular reflectance, and the shaded grey bar indicates how well human judgements can be predicted by data from other humans (the “noise floor”, or lowest possible model error).

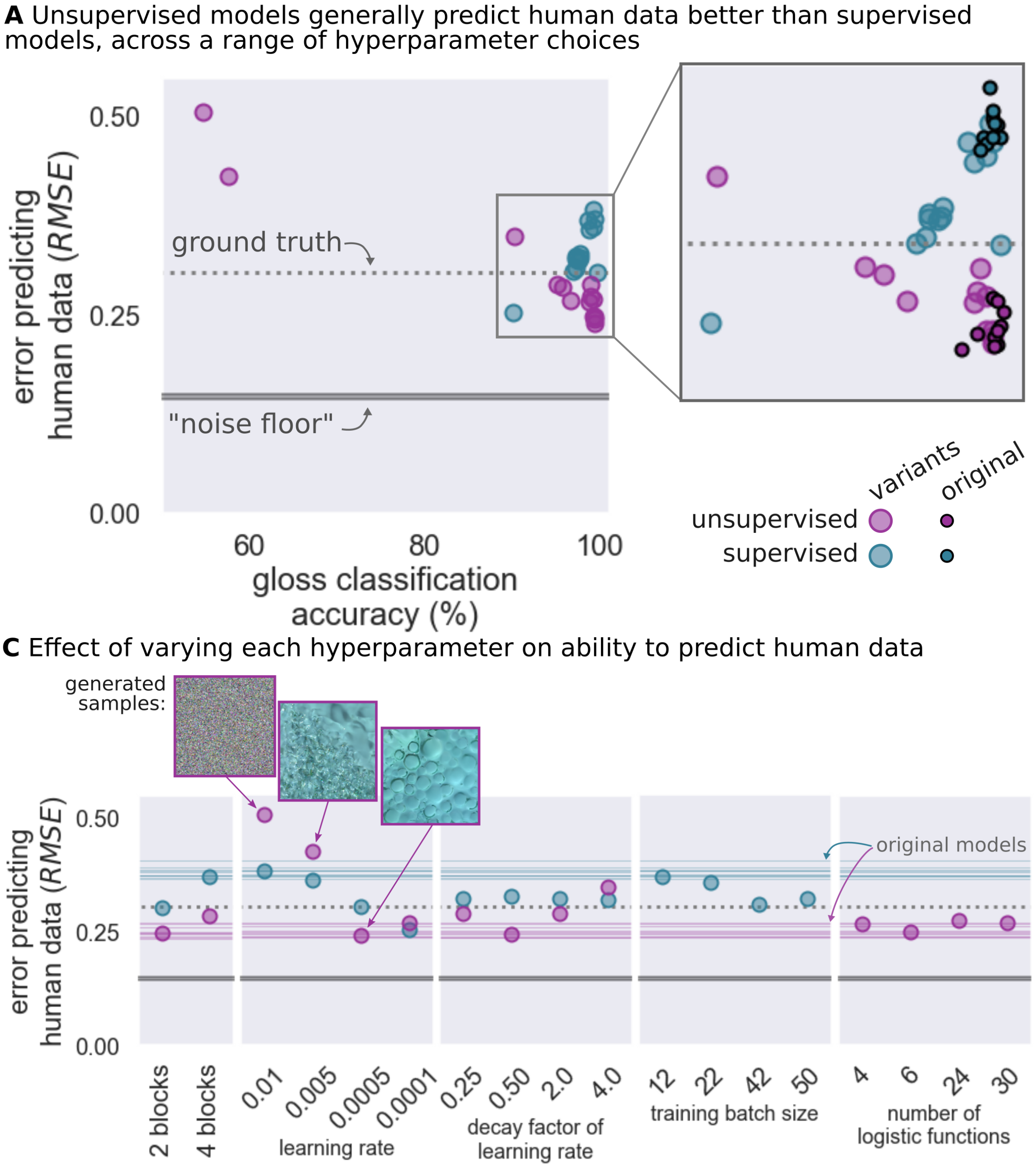


**Supplementary Figure 5: Main results are robust to hyperparameters of networks.**
**(A)** Scatterplot showing (y-axis) error in predicting human gloss judgements (average RMSE across three psychophysical experiments) against (x-axis) accuracy of high-vs-low gloss classifier trained on each network's 10D latent code, for all 14 hyperparameter variants of the (purple) unsupervised PixelVAE and (teal) supervised Resnet model. Inset shows a magnified version of the region in which most models lie, excluding variants that failed to train well. Smaller black-outlined dots show the range of performances for the 10 original training instances of both model types. The dotted grey line indicates how well human judgements can be predicted by ground truth specular reflectance, and the shaded

grey bar indicates how well human judgements can be predicted by data from other humans (the “noise floor”, or lowest possible model error).

**(B)** Performance in predicting human gloss judgements (y-axis) for each of the 28 hyperparameter variant networks. Unsupervised networks were generally superior to supervised networks, with the exception of the two unsupervised networks with large learning rates which failed to converge during training. Inset images are samples generated from each of the PixelVAE networks with learning rates of 0.01, 0.005, and 0.0005, showing that at the largest learning rates, the model fails to learn the structure in the data. Models that were able to train, generally well predicted human perceptual judgements. The noise floor and ground-truth performance are shown as in A. Faint horizontal lines indicate the performance of each of the ten training instances of the original unsupervised (purple) and supervised (teal) models, giving an indication of the expected variation in performance due to differences in random initialisation.

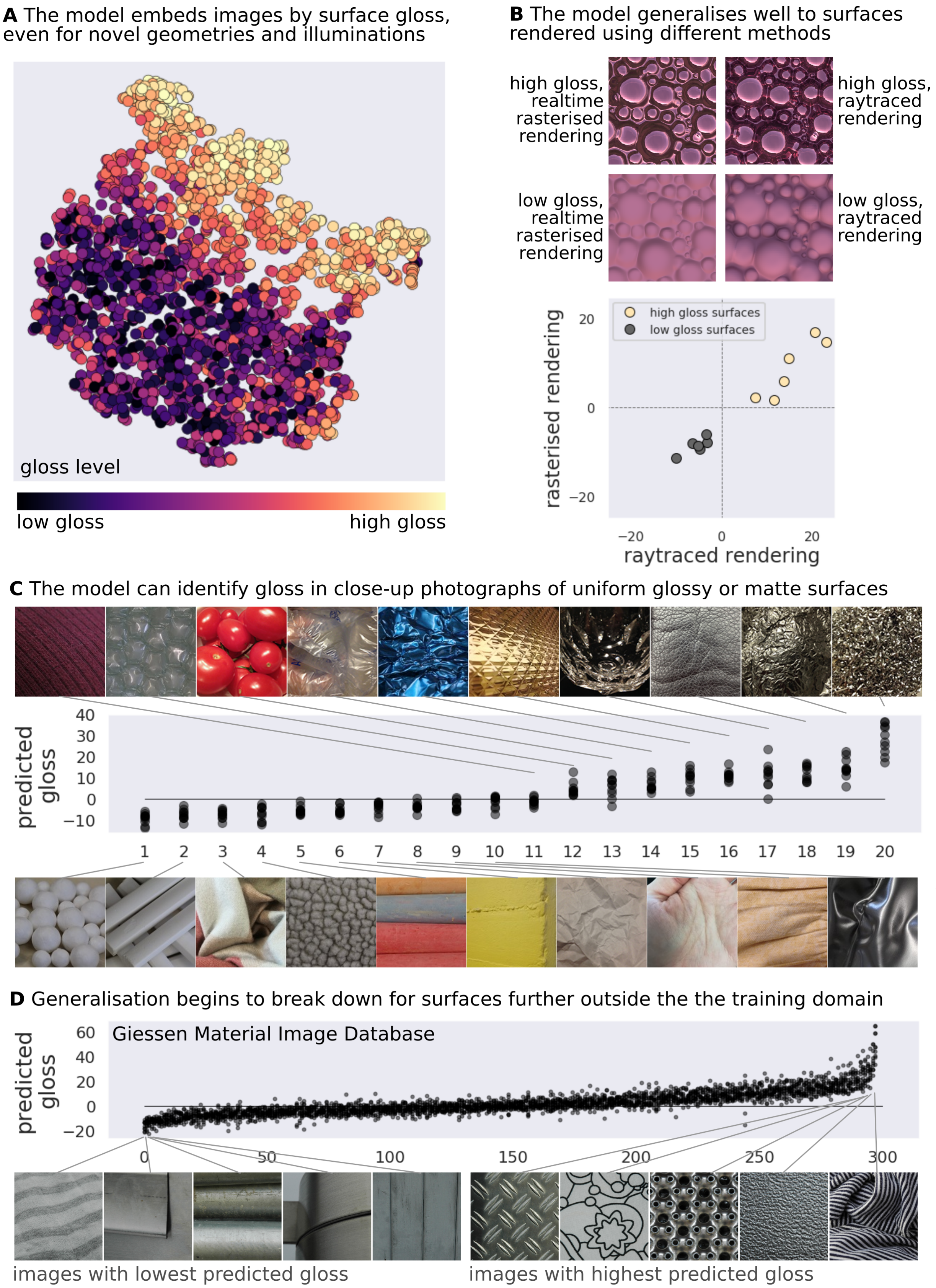
A The model embeds images by surface gloss, even for novel geometries and illuminations
gloss level
low gloss
high gloss
B The model generalises well to surfaces rendered using different methods
high gloss, realtime rasterised rendering
high gloss, raytraced rendering
low gloss, realtime rasterised rendering
low gloss, raytraced rendering
high gloss surfaces
low gloss surfaces
rasterised rendering
raytraced rendering
20
0
−20
C The model can identify gloss in close-up photographs of uniform glossy or matte surfaces
predicted gloss
40
30
20
10
0
−10
1 2 3 4 5 6 7 8 9 10 11 12 13 14 15 16 17 18 19 20
D Generalisation begins to break down for surfaces further outside the the training domain
Giessen Material Image Database
predicted gloss
60
40
20
0
−20
0 50 100 150 200 250 300
images with lowest predicted gloss
images with highest predicted gloss

**Supplementary Figure 6 [previous page]: Generalisation to images outside the training set.**
**(A)** The trained networks organise images lawfully by material even for renderings that fall outside the parameters of the training dataset. Plot shows tSNE embedding of 2500 images generated using five surface geometries and eight lighting environments never sampled from within the network's training dataset (see Methods: *Geometry and lighting generalisation test set)*. Points are colour coded by the strength of specular reflectance (dark purple = matte; cream = highest gloss).
**(B)** Example images (top) of the same surface geometry with either high or low gloss, created using either approximate rasterised rendering (left two images) as in the experimental training and testing datasets, or physics-simulating raytraced rendering (right two images). Scatterplot (bottom) of gloss level predicted by the unsupervised PixelVAE models for each of 12 such surfaces when rendered using rasterisation as in the original experiments (x-axis) or ray-tracing (y-axis). Gloss values are closely correlated, and all images were correctly classified (i.e. high-gloss images receive positive gloss values, and low-gloss images receive negative values), regardless of rendering method.
**(C)** Predicted gloss (y-axis) for twenty photographs of real-world glossy or matte surfaces, sorted by average predicted gloss. Each dot shows the prediction of one training instance of the unsupervised model. Different training instances make similar gloss predictions, and all but one surface is correctly categorised (i.e. matte surfaces receive negative gloss values (bottom row) and glossy surfaces receive positive values (top row)).
**(D)** Predicted gloss for all 300 images of assorted wood, fabric, metal and stone surfaces comprising the Giessen Material Image Database (https://www.allpsych.uni-giessen.de/MID/), sorted by average predicted gloss. Bottom row shows the five images with the lowest (left) and highest (right) predicted gloss. Although the model correctly identifies gloss in e.g. polished metal surfaces, it also incorrectly predicts high gloss values for surfaces with high-contrast textures, such as patterned fabrics.

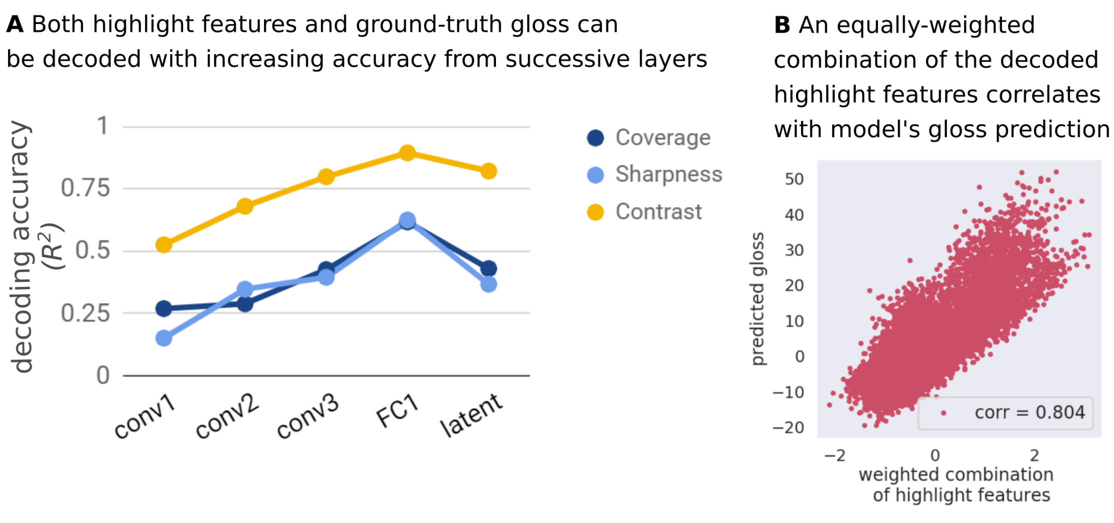


**Supplementary Figure 7. Highlight coverage, sharpness and contrast correlate with model-predicted gloss level.**

**(A)** The coverage, sharpness and contrast of highlights can be decoded from representations within one of the trained networks (here, we chose to investigate the model training instance with highest correlation with human-perceived gloss in Experiment 1). The decodability of mid-level highlight features increases throughout the network's layers, until the bottleneck latent code, in which there is a slight decline. (However, note that the dimensionality of the feature space reduces from 1000 units in the final fully connected layer to only 10 in the latent layer, while only minimally affecting gloss classification performance).

**(B)** Combining (via equal weighting) the estimates of the three highlight features, as decoded from the fully-connected layer immediately preceding the latent layer, yields a summary highlight measure which correlates well with the gloss level predicted from the latent layer.
