# Supplementary material for: Unsupervised learning predicts human perception and misperception of gloss
Source: Nat Hum Behav. 2021 May 6;5(10):1402–17. doi: 10.1038/s41562-021-01097-6 (PMC8526360; doi:10.1038/s41562-021-01097-6)
Supplement: Supplementary file 2 — Reporting Summary [file 41562_2021_1097_MOESM2_ESM.pdf]

nature research

Corresponding author(s): Katherine Storrs

Last updated by author(s): Feb 24, 2021

Reporting Summary

Nature Research wishes to improve the reproducibility of the work that we publish. This form provides structure for consistency and transparency in reporting. For further information on Nature Research policies, see our Editorial Policies and the Editorial Policy Checklist.

Statistics

For all statistical analyses, confirm that the following items are present in the figure legend, table legend, main text, or Methods section.

n/a	Confirmed	
☐	☒	The exact sample size (*n*) for each experimental group/condition, given as a discrete number and unit of measurement
☐	☒	A statement on whether measurements were taken from distinct samples or whether the same sample was measured repeatedly
☐	☒	The statistical test(s) used AND whether they are one- or two-sided<br>*Only common tests should be described solely by name; describe more complex techniques in the Methods section.*
☒	☐	A description of all covariates tested
☐	☒	A description of any assumptions or corrections, such as tests of normality and adjustment for multiple comparisons
☐	☒	A full description of the statistical parameters including central tendency (e.g. means) or other basic estimates (e.g. regression coefficient) AND variation (e.g. standard deviation) or associated estimates of uncertainty (e.g. confidence intervals)
☐	☒	For null hypothesis testing, the test statistic (e.g. *F*, *t*, *r*) with confidence intervals, effect sizes, degrees of freedom and *P* value noted<br>*Give P values as exact values whenever suitable.*
☒	☐	For Bayesian analysis, information on the choice of priors and Markov chain Monte Carlo settings
☒	☐	For hierarchical and complex designs, identification of the appropriate level for tests and full reporting of outcomes
☐	☒	Estimates of effect sizes (e.g. Cohen's *d*, Pearson's *r*), indicating how they were calculated

Our web collection on statistics for biologists contains articles on many of the points above.

Software and code

Policy information about availability of computer code

Data collection	Human data collection: PsychoPy 3.1 with Python 3.6; Deep neural network implementation and analysis: Tensorflow 1.14 with Python 2.7
Data analysis	All analyses of human and model data were performed in Python 2.7 or 3.6, using numpy v1.16.5, scikit-learn v0.21.3 and/or pinguoin v0.3.10 packages.

For manuscripts utilizing custom algorithms or software that are central to the research but not yet described in published literature, software must be made available to editors and reviewers. We strongly encourage code deposition in a community repository (e.g. GitHub). See the Nature Research guidelines for submitting code & software for further information.

Data

Policy information about availability of data

All manuscripts must include a data availability statement. This statement should provide the following information, where applicable:

- Accession codes, unique identifiers, or web links for publicly available datasets
- A list of figures that have associated raw data
- A description of any restrictions on data availability

All human and model data are available on Zenodo at http://doi.org/10.5281/zenodo.4495586, along with custom analysis code that reproduces all figures and statistical tests involving human data.

Field-specific reporting

Please select the one below that is the best fit for your research. If you are not sure, read the appropriate sections before making your selection.

☐ Life sciences ☒ Behavioural & social sciences ☐ Ecological, evolutionary & environmental sciences

For a reference copy of the document with all sections, see nature.com/documents/nr-reporting-summary-flat.pdf

Behavioural & social sciences study design

All studies must disclose on these points even when the disclosure is negative.

Study description	Quantitative psychophysics experiments measuring human visual perception.
Research sample	Three groups of 20 human observers were recruited for the five psychophysical experiments. In Experiments 1 and 2, participants were 16 women and 4 men with a mean age of 23.5. In Experiment 3a, participants were a different group of 16 women and 4 men, also with a mean age of 23.5 In Experiments 3 and 4, participants were 14 women and 6 men with a mean age of 24.3. Participants were primarily undergraduate students from the psychology programme at Justus Liebig University Giessen, Germany. Visual perception of materials likely varies somewhat with environmental and cultural exposure to different materials. We consider our sample of educated young adults to be representative of visual material perception within industrialised Western countries, but not necessarily representative of all humans.
Sampling strategy	A predetermined sample size of 20 participants was used in all experiments, as erring slightly above the conventional sample size in psychophysical studies of mid-level perception, e.g. groups of 12 in van Assen et al (2018) Current Biology (10.1016/ j.cub.2017.12.037), groups of 8-23 in Marlow et al (2012) Current Biology (https://doi.org/10.1016/j.cub.2012.08.009). Participants were recruited through the university's experimental volunteer system until the quota of 20 was filled for each experiment.
Data collection	All data were collected by having participants view a computer screen and respond via keyboard presses. Data collection was conducted by a research assistant who understood the broad programme of research but was blind to specific hypotheses in each experiment. Only the participant and research assistant were present in the room during data collection.
Timing	Data were collected between 12th April 2019 and 16th August 2019
Data exclusions	No data were excluded from analyses.
Non-participation	No participants dropped out, declined participation, or were excluded from participating.
Randomization	All experimental designs are within-subjects, so all participants took part in all conditions (with trials from different conditions presented in a random order). Assignment to each experiment was on the basis of which participants signed up to volunteer at the time we were running each experiment. Six individuals participated in two different experiments at separate times, but received no information about the design or hypothesis of the experiments after their first session.

Reporting for specific materials, systems and methods

We require information from authors about some types of materials, experimental systems and methods used in many studies. Here, indicate whether each material, system or method listed is relevant to your study. If you are not sure if a list item applies to your research, read the appropriate section before selecting a response.

Materials & experimental systems

n/a	Involved in the study	
☒	☐	Antibodies
☒	☐	Eukaryotic cell lines
☒	☐	Palaeontology and archaeology
☒	☐	Animals and other organisms
☐	☒	Human research participants
☒	☐	Clinical data
☒	☐	Dual use research of concern

Methods

n/a	Involved in the study	
☒	☐	ChIP-seq
☒	☐	Flow cytometry
☒	☐	MRI-based neuroimaging

Human research participants

Policy information about studies involving human research participants

Population characteristics	See above.
Recruitment	Participants volunteered via a university experimental volunteer system. They were primarily undergraduate psychology

Recruitment	students, and are likely more familiar with visual perception science and associated experimental methods than the average person. Since data analyses hinged on comparing how well a range of detailed predictions from different models could predict human perception across the specific experimental images used, any general knowledge participants had of material perception would not plausibly benefit any particular model in our tests.
Ethics oversight	Ethics approval was granted by the local ethics committee of Justus Liebig University Giessen, Germany.

Note that full information on the approval of the study protocol must also be provided in the manuscript.
