# Supplementary material for: Unsupervised learning predicts human perception and misperception of gloss
Source: Nat Hum Behav. 2021 May 6;5(10):1402–17. doi: 10.1038/s41562-021-01097-6 (PMC8526360; doi:10.1038/s41562-021-01097-6)
Supplement: Supplementary file 3 — Peer Review Information [file 41562_2021_1097_MOESM3_ESM.pdf]

natureresearch

Peer Review Information

Journal: Nature Human Behaviour
Manuscript Title: Unsupervised Learning Predicts Human Perception and Misperception of Gloss
Corresponding author name(s): Katherine R. Storrs

Editorial Notes:

Reviewer Comments & Decisions:

Decision Letter, initial version:

17th August 2020

Dear Dr Storrs,

Thank you once again for your manuscript, entitled "Unsupervised Learning Predicts Human Perception and Misperception of Gloss", and for your patience during the peer review process.

Your Article has now been evaluated by 3 referees. You will see from their comments copied below that, although they find your work of considerable potential interest, they have raised quite substantial concerns. In light of these comments, we cannot accept the manuscript for publication, but would be interested in considering a revised version if you are willing and able to fully address reviewer and editorial concerns.

We hope you will find the referees' comments useful as you decide how to proceed. If you wish to submit a substantially revised manuscript, please bear in mind that we will be reluctant to approach the referees again in the absence of major revisions. We are committed to providing a fair and constructive peer-review process. Do not hesitate to contact us if there are specific requests from the reviewers that you believe are technically impossible or unlikely to yield a meaningful outcome.

In the revision of your manuscript, we ask you to focus on two editorial priorities.
First, you must strengthen the base of evidence for your results, responding to methodological concerns raised by Reviewers #2 and #3 through further analysis. Second, we ask that you respond to the list of issues raised by Reviewer #3 (primarily points 1-5), carefully revisiting how you describe the contribution of your work and ensuring that competing and previous accounts receive a fair treatment. As a general principle, any overstatements and novelty claims must be avoided.

Finally, your revised manuscript must comply fully with our editorial policies and formatting

requirements. Failure to do so will result in your manuscript being returned to you, which will delay its consideration. To assist you in this process, I have attached a checklist that lists all of our requirements. I have also attached a template manuscript file that exemplifies our policies and formatting requirements. If you have any questions about any of our policies or formatting, please don't hesitate to contact me.

If you wish to submit a suitably revised manuscript we would hope to receive it within 6 months. We understand that the COVID-19 pandemic is causing significant disruptions which may prevent you from carrying out the additional work required for resubmission of your manuscript within this timeframe. If you are unable to submit your revised manuscript within 6 months, please let us know. We will be happy to extend the submission date to enable you to complete your work on the revision.

With your revision, please:

• Include a “Response to the editors and reviewers” document detailing, point-by-point, how you addressed each editor and referee comment. If no action was taken to address a point, you must provide a compelling argument. This response will be used by the editors to evaluate your revision and sent back to the reviewers along with the revised manuscript.

• Highlight all changes made to your manuscript or provide us with a version that tracks changes.

Please use the link below to submit your revised manuscript and related files:

[REDACTED]

Note: This URL links to your confidential home page and associated information about manuscripts you may have submitted, or that you are reviewing for us. If you wish to forward this email to co-authors, please delete the link to your homepage.

Thank you for the opportunity to review your work. Please do not hesitate to contact me if you have any questions or would like to discuss the required revisions further.

Sincerely,

Marike Schiffer

Marike Schiffer, PhD
Senior Editor
Nature Human Behaviour

Reviewer expertise:

Reviewer #1: Visual perception, DNNs

Reviewer #2: Visual perception, DNNs

Reviewer #3: Visual perception, DNNs

REVIEWER COMMENTS:

Reviewer #1:
Remarks to the Author:
This paper explores how unsupervised deep learning models, specifically PixelVAE can explain and predict human gloss perception. It reports 4 different experiments, comparing behavioural data and model data, using different models as references and baselines.

Comparing DNNs to human behaviour is a very popular and active research area. In visual perception, it is more common to study object recognition. I find that studying lower level feature perception is very relevant, and in particular glossiness.

I find the paper to be very clear, and I enjoyed reading it. In particular, the different computational experiments, and how unsupervised DNNs can predict human behaviour better than supervised DNN in glossiness prediction and other supervised DNNs on object or texture recognition.

It's interesting that the object features show less error than the supervised model on glossy images. It could be because the supervision is too narrow, only 2 labels. Also that object DNN features are better than texture features. I'm curious to know more about the authors insights about this.
For instance, for experiment 1, why not compare to a model that has been trained on ground truth glossy ratings from 1 to 6, instead of high and low gloss only?

The naming of the experiments, experiment 1-4 is a bit confusing and hard to follow in the text and relate them in the figures. A suggestion could be to use more meaningful names for them. Maybe adding a feature with a methods summary for each of the experiments, or a brief summary for each of them in results would be helpful.

Minor comments:
10 instances of the models, means that that they only differ in the initialisation weights? It is not clear when first introduced.

Reviewer #2:
Remarks to the Author:
Summary:
Storrs and Fleming show that PixelVAE trained on images of naturalistic textures learns an unsupervised representation that disentangles image properties (such as gloss, light field, and surface relief) and predicts human gloss perception judgements on a per-image basis better than a variety of strong controls.

Comments:

I think this paper is excellent. It very clearly and beautifully exemplifies the type of careful model-

comparison that I think is the most informative way to approach questions in our field going forward. I think the paper: (1) addresses an interesting problem, (2) is written clearly, and (3) achieves well-motived and convincing results. [NB: I have also reviewed a previous version of this paper. I thought it was very good then, and worthy of publication. It is *significantly* stronger now.] I am highly positive on publication of this paper.

The work is especially good at including many important control models to compare to PixelVAE. In some ways, the key strength of this paper is the fact that so many real (as opposed to strawman) controls are shown. Bravo!

Why do I care about these controls so much? It's because the main piece of evidence that shown here that PixelVAE is an advance over previous unsupervised visual models, is a fairly unusual thing: consistency with human judgements on glossiness. That's not an observable that has previously been established as useful for measuring correctness of a model of visual representation. Frankly it seems kinda weird and a little bit idiosyncratic. I could imagine readers asking "Why did you choose to study glossiness? Why not something other more useful visual property such as shape or contrast?" I think the authors have made some good conceptual arguments for justifying this choice of observable. But they've actually done something stronger. Given that the authors show that across a wide range of strong controls, one model model is a significantly better account of their chosen observable than all the others, that *by definition* makes their chosen observable a good test of model correctness. In other words, the way we know if an observable is strong is by how well it separates bad vs good vs really good models of the human visual system. To my understanding, the authors here don't really care about "glossiness" as such as an endpoint, but rather have alighted on it as a quantitatively characterizable feature of the human visual system that serves benchmark for model comparison (maybe the discussion could make this a little more explicit?). To the extent that they have the widest possible range of comparison models and show that their glossiness observable differentiates between these models, the more convincing this result is. I think what they've done is pretty convincing.

One small concern (with two related instantiations):

-- I'm slightly worried about how some of the results might be dependent on various hyperparameters of the architectures and training procedures used. For example, training hyperparameters such as batch size, learning rate, etc. For example, the gloss-supervised resnet was trained with "batch size of 32 and a learning rate of 0.001" (though it is not said how the learning rate was decreased during training -- this is an important detail that should be included). Were these varied at all? It has been my experience (and than of many others) that things such as these little details can matter a lot in the final performance of a network. Typically, it's a bit dicey to make a comparison between one type of network (PixelVAE) and another (e.g. gloss-supervised resnet) without doing a hyperparameter search to ensure that good values of these parameters have been chosen for each network type. One doesn't want to unfairly tip the scales toward one class of networks by having chosen better hyperparaterms for it than for the comparison class. It would be great if the authors could do something to address this concern.

-- A similar concern arises w.r.t. layer of readout. It's not obvious that the "right" layer to read out gloss predictions is at the last layer of the deep network. It might be the case that good representations for those things exist in an early layer, especially for a network that's trained for a meaningful semantic task such as categorization. I could easily imagine doing a dimension reduction

at an intermediate layer and then learning SVMs (or whatever readout mechanism is used) downstream from that. If the authors could do something to evidence robustness of their results to this choice that would be great.

Reviewer #3:
Remarks to the Author:
This beautiful paper demonstrates that a deep neural network (DNN) can learn to mimic human perception of surface glossiness, without being taught explicitly via human-labelled examples of glossiness or physical parameters. In this sense the DNN is “unsupervised”. Instead the DNN implicitly learns glossiness by aiming to compress images into more efficient descriptions and to produce new images using that efficient code. The analysis suggests that human perception of “gloss” is determined by image statistics not by reconstruction of ground truth physical properties.
The study is thoroughly done, and its components clearly described, with helpful compact figures. It is a pleasure to read, and hard to find areas for improvement.
My “But” follows now. The “But” is related to the big idea wrapping it all. The idea is that the success of this unsupervised network in mimicking gloss perception and disentangling physical image-formation features in its latent code demonstrates a radical step forward in understanding of human vision. The idea is pitted against “inverse optics” (a bit of a straw man here) by claiming that the latter requires that the human visual system have access to the ground truth of the physical world it is trying to recover. The big idea set out here as new is that the brain can learn what is in images only, not what is in the world, because the visual system can access only images of the world, not the world itself. I do like the way the paper demonstrates the idea via a comprehensive DNN model of gloss perception. But the claim for its newness is a stumbling block for me.
A little more on why the claim seems overdone, with implicit suggestions as to how to soften it:
1. The notion, that the statistical regularities within the images – the spatial variation of RGB pixel values within individual images and across populations of images – carry information about the physical scene that gave rise to the image, is not new. It informs many “mid-level” classes of models of visual perception, and especially for gloss perception (Refs 80 and 81 in the paper, e.g.). The new idea is that it is the image statistics that the visual system is trying to learn, not the properties of the physical scene that gave rise to it.
Yet the inverse optics approach is also firmly tied to image statistics through estimating likelihoods that given physical scenes give rise to observed images, conditioned on prior assumptions , or natural constraints. The prior assumptions represent the prior probabilities of physical properties that give rise to the scene. These probabilities are also embedded in and would also be derivable from the DNN trained here. The DNN is trained on spatially smoothly varying bumps, with uniform body surface reflectance and varying specular reflectance, under a fixed collection of light fields. The probabilities of pixel values will map onto those probabilities of surface parameters. It’s that complex mapping which the analytical framework of “inverse optics” models attempt to describe. The DNN learns the mapping more thoroughly, and embeds it in its multiple layers. But it still means that the DNN is encoding the mapping, at the heart of which is the notion that a physical scene generates the image. At a brass tacks level, this isn’t different from the notion that a physical scene generates an image, which is at the heart of the “inverse optics” approach.
Put another way: The inverse optics approach isn’t a model of development but a description of the mapping between scene and image. The DNN is also a description of the mapping between scene and image, and also does not provide a model of how the visual system develops this mapping. As noted in the discussion, the network architecture is not a physiological simulation. The key notion is that

compressing the image statistics information into fewer dimensions via efficient coding leads to features within which specular reflectance, lighting direction, etc are parametrised. In my view, this key notion is not qualitatively different from our understanding that human gloss perception is related to the mapping between physical scene parameters and image pixel value variations.
2. Neither approach provides a testable hypothesis as to how the human visual system learns the mapping, in neural development. Both provide – at different levels of comprehensibility – an existence proof that the mapping depends on the constraints of the environmental input. The DNN is less comprehensible than an analytic summary. In this sense, the DNN is perhaps less informative than, e.g. the descriptions derived in mid-level models such as Ref 16, because the DNN's latent code is more black-box like.
3. The fact that in image formation confounds arise between lighting, shape and specular reflectance is already known. Where these unknowns are inherently confounded in the known image intensity values, no computational model, human or DNN is able to disentangle them. The confounding is a prior property, not one that emerges from unsupervised learning. So I disagree with the final statement of the Discussion that "unsupervised learning may account for failures of constancy" and would instead suggest that "loss of information in the image generation process" may account for failures of constancy. I might also rewrite the sentences in lines 28-32 as "Via extracting summary statistics, unsupervised deep learning achieves decomposition of image information into distinct physical causes."
4. The failure of the DNN to predict the human inability to see gloss when highlights do not align with surface geometry (as depicted from other visual cues) is significant. This failure calls into question the meaning of the model in separating surface relief from material properties. The organisation of surface relief in the latent code is likely a result of the organisation of second-order parameters such as coverage, sharpness and contrast. As surface relief smoothly varies in a connected surface the spread of highlights will also vary in a regular way.
In this sense the DNN isn't doing (or proving that the human visual system does) something qualitatively different from what the inverse optics models (or previous analyses of glossiness perception) but only doing it quantitatively better, by putting more muscle into the predictive model.
5. Another way to describe the goal of the paper is that it seeks to explain why the specular reflectance of materials alone does not predict glossiness perception perfectly. The answer isn't contained here. The DNN reproduces errors – in predicting that humans would perceive glossiness where specular reflectance is low and vice versa - but doesn't explain why it or the human makes these "misperceptions". In this sense, the DNN does not reveal anything fundamental about human vision that's not already known.

The above comments are not meant to downplay the importance of the construction of the DNN itself and the experiments in which its performance are compared with human perceptual behaviour. Neither are the following. I apologise that they are not in chronological order.

Specific comments on methodology:
6. Generalisability: Although a massive number of images were generated to train the DNN and the other networks with which it was compared, these images were nonetheless of a very restricted world: a patch of bumpy terrain, uniform in diffuse and specular reflectance across its surface, varying only in heights of bumps, and not much in cross-sectional circumference of bumps, and always viewed from directly above. Even with a large variation in camera angle and location, combined with 6 possible illuminations, the generative scenes are limited in scope. So the question arises as to how the DNN would do on images of other sorts of scenes with specular scenes? The question is whether the emergence of the material/lighting disentanglement in the DNN is a special case that results from the

constrained nature of the image generation process. This question is fairly well addressed by the tests summarised in Figure 7 and supp Figure 4. But the photographs seem specially chosen to resemble the original training images (front-parallel view, limited surface relief). It would be helpful to see more analysis of where and how the network fails.

7. In Experiments 1,3, and 4: are the images used in these experiments generated in the same way as for the main training set (i.e. from the original 6 HDRIs, and the original bimodal specular reflectance component distributions, and surface relief parameters)? And, therefore, for the comparison of the three PixelVAE DNNs, the images used were all from the main training set? I.e. none of the novel geometries or novel light fields were used to generate the images used in Experiments 1,3, and 4? Please clarify this in the manuscript.

One reason I ask this is because some of the novel geometries in Figure 7A I find very hard to read in terms of glossiness. It would be interesting to know what whether in these cases the glossiness predicted by the original PixelVAE model (or indeed one of the other two Pixel VAE models) agree with human perception of glossiness in these images.

8. The network is trained to summarise a training set of images by “probabilistic distributions of pixel values that captured the structure and variability in and across the training images across all spatial scales” and then to “generate plausible novel samples from that visual world”. It does so with two streams: the ‘conditioning’ network and the ‘autoregressive’ stream. It’s the former that encodes the images into a 10D latent code, and this is the code that’s used to test the success of the network in experiments 1,3 and 4.

My question: Does the autoregressive network feed into the conditioning stream and influence the development of the 10D latent code? It appears to in Figure 1, but this is not clear from the methods, or from the main text. It is not clear whether the success of the network in predicting gloss and gloss constancy (i.e. specular reflectance or human perception) depends only on the latent code, or on the spatial probability distribution learned by the autoregressive stream. The autoregressive stream is clearly necessary for generating new images, e.g. as used in Experiment 2, but it is not clear whether it is necessary for the other tests. In several places throughout the manuscript, it’s said that the success of the network depends on its two learning objectives – data compression and spatial prediction – but I am left wondering how essential the second learning objective is to the success demonstrated in experiments 1,3 and 4. These experiments depend on gloss ratings derived from an SVM classification via the 10D latent code only.

9. The related question is to what do the learning objectives of the unsupervised model correspond, in human development? Since the question is posed as to why or how a supervised training would get its supervision, we should ask the equivalent question of the unsupervised model. Why or how would the unsupervised model set its objectives in this way? The unsupervised human visual system would not necessarily need to develop the ability to synthesise novel images, but it might indeed wish to develop optimal encoding strategies, given physical limitations on neural mechanisms. Therefore, the balance between optimal encoding vs. ability to generate novel images might need some restating.

10. P5 line 10: “As models of human gloss perception, it is unclear from where the necessary training labels could come.” The statement implicitly refers to human development. I believe that, as stated earlier in the introduction, the idea is that a developing visual system would not be able to employ information from other senses as to the physical properties that give rise to gloss. The implication is that this problem is specific to gloss perception. But for learning of visual categories more generally, it remains a problem to determine how the supervised learning takes place in development. And the simple answer, that the supervision comes from other humans providing the training labels, would equally apply well to gloss perception.

11. The ability of the DNN to predict human perception of glossiness in synthetic (rendered) images was compared with at least 6 other models; a hugely commendably thorough approach. Importantly,

the model was compared with a simpler model based on image statistics, using the single statistical measure of luminance histogram skewness. That single measure doesn't predict human glossiness as well as the DNN does, but it seems likely that it contributes, given the previous demonstrations of its success in predicting glossiness. Therefore it would help us gain insight into what the DNN is doing if we were to see how luminance skewness paints the clusters in the latent code, similarly to the physical factors of light angle and light field, for example. Similarly, it would be interesting to see how the texture features of Ref 80 paint the clusters.

12. The Unity3D standard shader: this shader allows the diffuse (body) and specular reflections components of a surface to have different properties. It's not clear from what's written here whether the base color refers only to the diffuse component, and whether the specular reflectance component was different, and/or whether it was spectrally flat (whether it varied with wavelength). It's also not clear whether the indirect reflections between the bumps in the images were physically accurately modelled. Again, the relevance of this comment is that the model the DNN is learning might be a simplified model of the world, constrained by the limitations of the standard shader model used to generate the images.

Minor comments:

P 18: The last 2 paragraphs (from line 5) describe what must be experiment 4, but it isn't labelled as such.

P 18:, line 19-21. ".. the arrangement of stimuli in the unsupervised model's latent space…" This sentence needs some unpacking for me. Is the implication that distances between stimuli, as measured in the multi-dimensional latent variable space of the DNN, correspond to differences in the estimated gloss of pairs of images? If that's what meant, then this doesn't, I think, follow directly from the data. The human data shows almost no variation in "deviation from constancy" for the positive deviations – i.e. a flat horizontal for positive values in Figure 5E – whereas the model predicts values with much more variation (from 0 to 10). The human data make the decision look categorical, whereas the model makes it look continuous. Thus, the organisation of the DNN's perceptual space appears qualitatively different from the human perceptual space. I might have misunderstood the sentence. In any case, it needs to be made clearer what's mean, and then justified better.

Figure 7 caption: (A) x-axis and y-axis are cited the wrong way round. (B) "were were"

Author Rebuttal to Initial comments

Dear Dr Schiffer,

Thank you for your comments and for the insightful reviews. The three reviewers raised some excellent points which have helped us extend our analyses and improve the framing of the work. Specifically, you asked for two areas of revisions:

"First, you must strengthen the base of evidence for your results, responding to methodological concerns raised by Reviewers #2 and #3 through further analysis."

We have performed seven new analyses to address these concerns:

1. Visualisations of how world factors are represented within all alternative models (new **Supplementary Figure 2**). Addresses Reviewer #2.
2. Visualisation of how luminance histogram skewness relates to the representations in our unsupervised model (**figure included in this response letter**). Addresses Reviewer #2.
3. Analysis of performance for intermediate layers within both unsupervised and supervised models (new **Supplementary Figure 4A**). Addresses Reviewer #2.
4. Analysis of performance for a new version of the supervised model, trained to report continuous gloss levels rather than to categorise gloss (new **Supplementary Figure 4B**). Addresses Reviewer #1.
5. Analysis of performance for twenty-eight newly trained models, which explore a wide range of hyperparameter settings in both the unsupervised and supervised models (new **Supplementary Figure 5**). Addresses Reviewer #3.
6. Analysis of generalisation in unsupervised model for images rendered using an alternative (slower but more physically faithful) rendering method (new **Supplementary Figure 6B**). Addresses Reviewer #3.
7. Broader analysis of generalisation in unsupervised model for natural photographs of real-world surfaces (new **Supplementary Figure 6C-D**). Addresses Reviewer #3.

"Second, we ask that you respond to the list of issues raised by Reviewer #3 (primarily points 1-5), carefully revisiting how you describe the contribution of your work and ensuring that competing and previous accounts receive a fair treatment. As a general principle, any overstatements and novelty claims must be avoided."

We have extensively reworked the Introduction and Discussion to better explain the motivation and contribution of our work. In doing so, it was very helpful to involve Barton Anderson, who provided a fresh perspective and helped us more clearly express the theoretical issues at stake. He now joins as an author. We have toned down several parts of the Introduction and Discussion that may have been overstated in the original submission.

Below we provide a point-by-point response to each of the reviewers' comments, and indicate where we have made changes in the manuscript to address them. Within the manuscript, changed text is indicated in blue.

Yours sincerely,

Katherine Storrs, Barton Anderson and Roland Fleming

Reviewer #1:

This paper explores how unsupervised deep learning models, specifically PixelVAE can explain and predict human gloss perception. It reports 4 different experiments, comparing behavioural data and model data, using different models as references and baselines.

Comparing DNNs to human behaviour is a very popular and active research area. In visual perception, it is more common to study object recognition. I find that studying lower level feature perception is very relevant, and in particular glossiness.

I find the paper to be very clear, and I enjoyed reading it. In particular, the different computational experiments, and how unsupervised DNNs can predict human behaviour better than supervised DNN in glossiness prediction and other supervised DNNs on object or texture recognition.

It's interesting that the object features show less error than the supervised model on glossy images. It could be because the supervision is too narrow, only 2 labels.

Both the pretrained object-recognition-supervised network and our gloss-classification-supervised network are around 99% accurate at classifying ground truth gloss. However, as you say, the object-trained DNN much better predicts human perceptual judgements. We agree that it is likely that the broader visual experience and more complex task of the object-trained DNN has created a more diverse set of visual features, whereas the gloss-supervised DNN has learned only those features relevant to its task. Our intention when designing the gloss-supervised DNN was not to demonstrate that *no* supervised network could predict human perception well, but rather that learning *human-like* gloss features is not trivial; a model can learn to report gloss almost perfectly within this dataset while failing to capture human perception.

We have added tSNE visualisations of the feature spaces within all alternative models as a new Supplementary Figure 2. This may help guide intuitions on why the object-recognition-supervised network predicts human perception better than the strictly gloss-categorisation-supervised network. While the gloss network dedicates its resources to separating high vs low gloss surfaces, the object-trained network has learned a more complex feature space, capturing information about, for example, surface bump height in our images.

Also that object DNN features are better than texture features. I'm curious to know more about the authors insights about this.

In aggregate over the three experiments, texture features predict human data as well as, and even slightly better than, object-trained DNN features (see Figure 6). We consider them similar models; the object-supervised DNN can be thought of as a data-driven way of optimising sets of high-level statistics that are useful for capturing local structures in natural images. The Portilla & Simoncelli texture statistics have a similar goal, but are a more hand-crafted implementation. The tSNE visualisation of both feature spaces now added in **Supplementary Figure 2** supports the interpretation that they capture similar image features.

For instance, for experiment 1, why not compare to a model that has been trained on ground truth glossy ratings from 1 to 6, instead of high and low gloss only?

We agree that predicting more continuous gloss values is an interesting objective for the supervised model. **We have now trained five instances of such a model, from different random initial weights. Supplementary Figure 4B** shows the performance of these new "gloss-regression" networks, trained to output a continuously-valued prediction of the true specular reflectance magnitude. The feature spaces learned by these networks do predict human gloss judgements somewhat better than those of the original gloss-categorisation networks. On average, they predict human judgements as well as ground truth specular reflectance, which makes sense since they can be thought of as a deep learning implementation of an "ideal observer". However, only the unsupervised models reliably predict human perception *better than* ground truth.

The naming of the experiments, experiment 1-4 is a bit confusing and hard to follow in the text and relate them in the figures. A suggestion could be to use more meaningful names for them. Maybe adding a feature with a methods summary for each of the experiments, or a brief summary for each of them in results would be helpful.

We have now given the experiments names in addition to numbers to aid the reader. Throughout text and figures, we now refer to them as "*Experiment 1: Gloss ratings*", "*Experiment 2: Gloss manipulation*", "*Experiment 3: Patterns of gloss constancy*" and "*Experiment 4: Degrees of gloss constancy*."

Minor comments:

10 instances of the models, means that that they only differ in the initialisation weights? It is not clear when first introduced.

Yes, that's correct. **We have clarified this on page 6, line 24**.

Reviewer #2:

Summary:

Storrs and Fleming show that PixelVAE trained on images of naturalistic textures learns an unsupervised representation that disentangles image properties (such as gloss, light field, and surface relief) and predicts human gloss perception judgements on a per-image basis better than a variety of strong controls.

Comments:

I think this paper is excellent. It very clearly and beautifully exemplifies the type of careful model-comparison that I think is the most informative way to approach questions in our field going forward. I think the paper: (1) addresses an interesting problem, (2) is written clearly, and (3) achieves well-motived and convincing results. (NB: I have also reviewed a previous version of this paper. I thought it was very good then, and worthy of publication. It is *significantly* stronger now.) I am highly positive on publication of this paper.

The work is especially good at including many important control models to compare to PixelVAE. In some ways, the key strength of this paper is the fact that so many real (as opposed to strawman) controls are shown. Bravo!

Why do I care about these controls so much? It's because the main piece of evidence that shown here that PixelVAE is an advance over previous unsupervised visual models, is a fairly unusual thing: consistency with human judgements on glossiness. That's not an observable that has previously been established as useful for measuring correctness of a model of visual representation. Frankly it seems kinda weird and a little bit idiosyncratic. I could imagine readers asking "Why did you choose to study glossiness? Why not something other more useful visual property such as shape or contrast?" I think the authors have made some good conceptual arguments for justifying this choice of observable. But they've actually done something stronger. Given that the authors show that across a wide range of strong controls, one model is a significantly better account of their chosen observable than all the others, that *by definition* makes their chosen observable a good test of model correctness. In other words, the way we know if an observable is strong is by how well it separates bad vs good vs really good models of the human visual system. To my understanding, the authors here don't really care about "glossiness" as such as an endpoint, but rather have alighted on it as a quantitatively characterizable feature of the human visual system that serves benchmark for model comparison (maybe the discussion could make this a little more explicit?). To the extent that they have the widest possible range of comparison models and show that their glossiness observable differentiates between these models, the more convincing this result is. I think what they've done is pretty convincing.

We thank the reviewer for the enthusiasm, and for previous suggestions, which had already helped significantly improve the paper.

While glossiness is a particularly pertinent testbed for models of perceptual inference, we and many others in the field also consider gloss perception to be an important topic in its own right, as evidenced by many papers on the topic by us and others in leading journals in recent years, e.g., Motoyoshi et al. (2007), *Nature*; Doerschner et al, (2011), *Current Biology*; Kim et al (2012) *Nature Neuroscience*; Marlow et al (2012) and (2015) *Current Biology*; Muryy et al (2013) *PNAS*, and (2016) *Proc Roy Soc*; Mooney & Anderson (2014) *Current Biology*, to name just a few. The reason gloss perception is especially challenging is because specular structure varies so dramatically as a function of the light field, which makes it a particularly useful domain to assess models because the perceptual response can systematically diverge from ground truth.

One small concern (with two related instantiations):

-- I'm slightly worried about how some of the results might be dependent on various hyperparameters of the architectures and training procedures used. For example, training hyperparameters such as batch size, learning rate, etc. For example, the gloss-supervised resnet was trained with "batch size of 32 and a learning rate of 0.001" (though it is not said how the learning rate was decreased during training -- this is an important detail that should be included).

Both the PixelVAE models and ResNets were trained with a learning rate of 0.001 and a 'decay factor' of 1.0, meaning that learning rate was decayed gradually by multiplying on each epoch by the value (1 - decay factor*(learning rate/number of epochs)), which equated to 0.999995 for the PixelVAEs, and 0.99995 for the ResNets. **We have included this information on page 26, lines 21-23 and page 27, line 5, and provided a summary in Supplementary Table 1.**

Were these varied at all? It has been my experience (and than of many others) that things such as these little details can matter a lot in the final performance of a network. Typically, it's a bit dicey to make a comparison between one type of network (PixelVAE) and another (e.g. gloss-supervised resnet) without doing a hyperparameter search to ensure that good values of these parameters have been chosen for each network type. One doesn't want to unfairly tip the scales toward one class of networks by having chosen better hyperparameters for it than for the comparison class. It would be great if the authors could do something to address this concern.

We agree on the importance of demonstrating robustness to changes in the specific hyperparameter values chosen. **We have now trained 28 new models, 14 each of the unsupervised PixelVAEs and supervised ResNets, which explore a wide range of training hyperparameter and architecture differences**. The details and results are provided in the new **Supplementary Figure 5**, **Supplementary Table 1**, and accompanying text in **Supplementary Methods and Results**. With the exception of two unsupervised networks which did not train effectively (those with the lowest learning rates), unsupervised networks robustly outperformed supervised networks in predicting human gloss judgements. The two outlier models simply did not learn their objective function, so it is little surprise that they also perform poorly at gloss disentanglement, or predicting human judgments.

The performances of the networks used in the main experiments appear to be in the range of typical performances across hyperparameter variations, although they tend toward the edges (the unsupervised nets perform better than average, and the supervised nets worse than average). This is likely due to two reasons. First, we deliberately selected image stimuli for the human experiments over which the original set of supervised and unsupervised networks "disagreed" (i.e., on the pattern or degree of gloss constancy predicted for sets or pairs of images in Experiments 3 and 4, respectively). Performances for the original models on this stimulus set are therefore likely to be more divergent than for other models or other stimulus sets. Second, in the hyperparameter explorations, all networks were trained for the same number of epochs as for the original models. Some hyperparameter variants, e.g., the PixelVAE models with more complex pixel likelihood models (i.e., described using a mixture of a larger number of logistic functions), may benefit from more training. Overall, however, the finding that the unsupervised model better predicts human data than the supervised one seems robust to specific hyperparameter choices.

-- A similar concern arises w.r.t. layer of readout. It's not obvious that the "right" layer to read out gloss predictions is at the last layer of the deep network. It might be the case that good representations for those things exist in an early layer, especially for a network that's trained for a meaningful semantic task such as categorization. I could easily imagine doing a dimension reduction at an intermediate layer and then learning SVMs (or whatever readout mechanism is used) downstream from that. If the authors could do something to evidence robustness of their results to this choice that would be great.

This is another excellent suggestion. **We have performed this analysis**, and find that representations in the unsupervised model better predict human gloss judgements than those in the supervised model, at all layers throughout the models. See **Supplementary Figure 4A**.

Reviewer #3:

This beautiful paper demonstrates that a deep neural network (DNN) can learn to mimic human perception of surface glossiness, without being taught explicitly via human-labelled examples of glossiness or physical parameters. In this sense the DNN is "unsupervised". Instead the DNN implicitly learns glossiness by aiming to compress images into more efficient descriptions and to produce new images using that efficient code. The analysis suggests that human perception of "gloss" is determined by image statistics not by reconstruction of ground truth physical properties. The study is thoroughly done, and its components clearly described, with helpful compact figures. It is a pleasure to read, and hard to find areas for improvement.

We thank the reviewer for the encouraging comments.

My "But" follows now. The "But" is related to the big idea wrapping it all. The idea is that the success of this unsupervised network in mimicking gloss perception and disentangling physical image-formation features in its latent code demonstrates a radical step forward in understanding of human vision. The idea is pitted against "inverse optics" (a bit of a straw man here) by claiming that the latter requires that the human visual system have access to the ground truth of the physical world it is trying to recover. The big idea set out here as new is that the brain can learn what is in images only, not what is in the world, because the visual system can access only images of the world, not the world itself. I do like the way the paper demonstrates the idea via a comprehensive DNN model of gloss perception. But the claim for its newness is a stumbling block for me.

In retrospect, we agree that pitting the work against 'inverse optics' failed to capture the essence of our contribution and the distinction we were trying to raise. Accordingly, **we have removed reference to inverse optics from the manuscript.**

The key unsolved problem we are trying to address is how the brain disentangles the complex wealth of regularities in images in order to (approximately) provide information about different distal properties, *without ever being given explicit information about the true state of the world*. How does the visual system become aware of the different 'kinds' of distal scene properties that contribute to image structure, when all it has access to is the proximal stimulus? This is an open challenge for all theories of vision. Our general claim is that many of the important structures in the outside world can in fact be discovered statistically through observation, in a data-driven way, given sufficiently powerful statistical learning methods.

We have modified the introductory framing of our paper as follows (page 3):

"Somehow the visual system infers the combination of distal scene variables that most plausibly explain the proximal sensory input (4,8–11). Yet, a major unsolved question is how the candidate explanations of proximal inputs came to be known in the first place. Our visual systems did not – and do not – have access to ground truth information about the number or kinds of distal sources of image structure that operate in the world. Any knowledge about the world must have been acquired from exposure to proximal stimuli over evolutionary and/or developmental time scales (12–14).

Here we explore the intriguing possibility that visual systems might be able to discover the operation of distal scene variables by learning statistical regularities in proximal images, rather than through learning an explicit mapping between proximal cues and known distal causes."

We elaborate on the novelty of our contribution in response to the comments below.

A little more on why the claim seems overdone, with implicit suggestions as to how to soften it:

1. The notion, that the statistical regularities within the images – the spatial variation of RGB pixel values within individual images and across populations of images – carry information about the physical scene that gave rise to the image, is not new. It informs many "mid-level" classes of models of visual perception, and especially for gloss perception (Refs 80 and 81 in the paper, e.g.).

The new idea is that it is the image statistics that the visual system is trying to learn, not the properties of the physical scene that gave rise to it.

We agree that the general idea that the brain learns to encode statistical regularities in natural images has a long and celebrated history in vision science, dating back at least to Barlow (1961). Indeed, Barlow observed that the statistical regularities and redundancies in images provide tell-tale signatures of the world-structures that create them. However, we think that our work goes beyond what has been done previously in several important ways. First, we provide not just a theory or general notion that this might be possible, but a real-world implementation of the idea in a working model. Second, we show that an unsupervised statistical learning framework can generalize beyond the efficient encoding of images in low-level vision to the discovery of (approximations to) distal physical variables in mid-level vision. **We clarify this point in the paper as follows (page 4)**:

"The idea that our perceptual systems exploit statistical regularities to derive information about the world has a long and venerable history in both psychology and neuroscience (19–21). For example, neural response properties in early visual cortex are well predicted by models trained to generate sparse codes for natural image patches (19,21–24). Unfortunately, such 'efficient coding' approaches have not yet scaled beyond the initial encoding of images. One of the main motivations for the present work was to determine whether such ideas could provide leverage into mid-level scene understanding, i.e., inferring the distal physical causes of sense data."

There are other points of difference between our approach and those articulated previously. One key difference is that our goal is to explain the *specific pattern of successes and errors* that humans make on an image-by-image basis. This is different from other approaches that focus on ideal performance in recovering some (known) distal scene property. One of our most significant findings is that unsupervised model predicts the successes *and* failures of human perception better than alternative models, based on either supervised learning or alternative features. This demonstrates that the acquired properties of the unsupervised learning model tested here are not a trivial consequence of the overall challenge of the task.

Yet the inverse optics approach is also firmly tied to image statistics through estimating likelihoods that given physical scenes give rise to observed images, conditioned on prior assumptions, or natural constraints. The prior assumptions represent the prior probabilities of physical properties that give rise to the scene.

We agree with that characterization of the inverse optics approach (although we are no longer using that term in our paper). But there are a number of unsolved problems created by this conceptualization. How did the visual system become aware of the set of physical properties that give rise to image structure? Through what specific computational process can knowledge of priors (or indeed likelihoods) about events in the outside world (and their relationship to image structure) be acquired, and what traces does the acquisition process leave in the perceptual judgments humans make? Distal physical properties (e.g., specular and diffuse reflectance, shape, illumination) had to be discoverable on the basis of the different impacts they have on image structure. We are trying to provide some insight into how the visual system became aware of these distal properties without any prior knowledge of the number or kinds of scene variables to be estimated.

These probabilities are also embedded in and would also be derivable from the DNN trained here. The DNN is trained on spatially smoothly varying bumps, with uniform body surface reflectance and varying specular reflectance, under a fixed collection of light fields. The probabilities of pixel values will map onto those probabilities of surface parameters.

We agree that an omniscient "experimenter" could demonstrate that the pixel likelihood distributions learned by the unsupervised DNN relate to the prior probabilities of scene properties like surface material in the network's training data. For example, the model is extremely unlikely to generate an image of a surface with a striped texture pattern, since such a surface never occurs in

its training world. Indeed, any visual system that can support useful reports and behaviours *must* in some sense embody a mapping between proximal and distal properties.

The crucial question is *how* a visual system can come to embody probabilities about diverse properties of the outside world, without first having access to those properties from which to learn. Indeed, it is one of the key things we are trying to show: not just that these probabilities are 'embedded in and derivable from the DNN' but that the DNN spontaneously clusters the variability in images in ways that closely accord with the distal scene variables, without any prior knowledge of either the number of distal variables to be estimated or any a priori knowledge of the physical constraints that shape them.

It's that complex mapping which the analytical framework of "inverse optics" models attempt to describe. The DNN learns the mapping more thoroughly, and embeds it in its multiple layers. But it still means that the DNN is encoding the mapping, at the heart of which is the notion that a physical scene generates the image. At a brass tacks level, this isn't different from the notion that a physical scene generates an image, which is at the heart of the "inverse optics" approach.

We appreciate the line of argument that the reviewer is raising here. **By removing all reference to inverse optics from the manuscript, we believe we have fully addressed this point**. But in the interest of scientific discussion, here are a few comments in response.

First, it's worth noting that this discussion is somewhat tangential to the main point we are trying to make. The inverse optics approach (or any approach that exploits physical constraints or priors about distal scene variables) needs to explain how the visual system acquired a representation specifying which different classes of distal scene properties there are to begin with. We are trying to provide some insight into this question. We show that it is possible to tease apart distal scene properties without being explicitly taught any mapping between pixels and distal scene variables. We think this is interesting as it potentially resolves an important open challenge for all theories of vision.

Second, we think the reviewer's sense of the term 'inverse optics' is not specific enough to distinguish between competing models of vision. In this sense, *any* visual system that succeeds at distinguishing different distal causes on the basis of proximal input must in some way encode a mapping between pixel values and (probabilities of) surface parameters, because that's built into the definition of 'succeeding' at the task.

However, this obscures an important distinction in what the competing models are trained to do. We think here is it crucial to emphasise the difference between the mappings learned by our supervised and unsupervised models. In a Bayesian perception framework, a supervised DNN is explicitly trained to report the posterior: *the probability of a scene property* (in our case, gloss), *given the information in the image*. Therefore, the *supervised* model can indeed be thought of as a machine learning implementation of an inverse optics model (albeit limited to inferring a single scene property). It receives true information about scene properties in its inputs during training and produces guesses about them as its output. The objective function used to train the network explicitly enforces the learning of the mapping between proximal and distal stimulus.

The unsupervised model, by contrast, has no conceptualisation of scene properties in either its inputs or outputs. The objective function makes no reference to any mapping between proximal and distal stimuli. It learns only *the probability of the sensory information* (i.e., the prior in the denominator in Bayes' rule); that is, it learns a model of the ways in which pixel values tend to vary within and across its training images.

So, to the extent that scene properties like gloss can be read out from the representations in both supervised and unsupervised models, they in some sense "encode a mapping" between proximal and distal. Yet we think there is nevertheless a substantial theoretical difference between a model that encodes the mapping because it has been explicitly trained to do so (which, as we and others

argue is biologically implausible), and one in which information about scene properties has spontaneously emerged in the process of modelling image statistics.

Put another way: The inverse optics approach isn't a model of development but a description of the mapping between scene and image. The DNN is also a description of the mapping between scene and image, and also does not provide a model of how the visual system develops this mapping. As noted in the discussion, the network architecture is not a physiological simulation. The key notion is that compressing the image statistics information into fewer dimensions via efficient coding leads to features within which specular reflectance, lighting direction, etc are parametrised. In my view, this key notion is not qualitatively different from our understanding that human gloss perception is related to the mapping between physical scene parameters and image pixel value variations.

We do not fully agree with this assessment. The DNN is not given any explicit information about the scene or even that images are the result of a mapping of a scene onto an image. In contrast to supervised learning approaches, or analytical inverse optics approaches, its objective function makes no reference to the outside world. It just gets a family of images and is trying to discover some latent variables that best capture the variability across the family of images it has been exposed to. It is not trivial that the set of latent variables discovered by the network map onto the distal scene variables that generated the images in the absence of any information about the number of variables that were varied, what those variables were, or in the absence of any supervised labelling.

Indeed, the representation that it learns does not perfectly align with the true physical properties of the world: and it is precisely the specific form of the deviations that make the model particularly interesting as a model of human perception. We find that the unsupervised network 'misclassifies' the latent variables in a manner that closely matches human behaviour. Moreover, we show that alternative mappings, learnt from the same image set and which objectively perform the task of estimating physical gloss just as well *do not* predict human perception. These are the points of 'qualitative' difference: *any* theory of perception is grounded on the recognition that there is a mapping between physical scene variables and image structure; our contribution is to provide some insight into how the set of physical scene variables could be discovered based solely on properties of the input.

We have significantly rewritten the introduction to the manuscript to address these points (p3-4). As mentioned above, we have removed all reference to inverse optics, so the tone and context in which we present our contribution has changed. We believe that as a result, there is no overstatement about the novelty of the work.

2. Neither approach provides a testable hypothesis as to how the human visual system learns the mapping, in neural development. Both provide – at different levels of comprehensibility – an existence proof that the mapping depends on the constraints of the environmental input. The DNN is less comprehensible than an analytic summary. In this sense, the DNN is perhaps less informative than, e.g. the descriptions derived in mid-level models such as Ref 16, because the DNN's latent code is more black-box like.

We agree that neither the approach of the current paper nor of 'inverse optics' provides a testable theory of neural development, and did not present the paper as such. We also do not believe that the DNN will replace other more hand-engineered approaches, such as those of Marlow, Kim and Anderson (2012) (now Ref 35 in the revised manuscript). Indeed, we continue to pursue such approaches ourselves in addition to using deep learning. However, are there in fact any analytical inverse optics model that successfully infer gloss from images and correctly predict human judgments? We believe no such model actually exists.

The different physical attributes that Ref. 35 used to characterize specular reflections also begs the same question as discussed in our response to 1. This paper did not reveal how the visual system extracted the specular component of reflection; rather, the authors of this work (the senior author of which is now a co-author of the present manuscript) demonstrated that certain properties of the

perceived specular component predicted *perceived* gloss. However, this, by itself, provides no insight into how the visual system identifies, segments, and assesses the dimensions of the perceived specular component. The DNN work we present here demonstrates that it's possible for an unsupervised system to learn that there's more than one kind of visual event class in the images used in these two studies, which cluster around distal scene variables.

While it is true that DNN's tend to be more 'black box like' than hand-coded models, it is worth noting that we are careful not to make any claims about a mapping between the inner workings of the DNN and the inner workings of the human visual system. We see them as one kind of tool that allows us to answer one kind of question, which is complementary to more traditional approaches.

Specifically, the level of analysis we are addressing is not so much algorithmic as computational. We are testing a hypothesis about what kinds of learning objectives allow us to infer useful latent variables from the input alone, and what consequences this has for human judgments.

Future work can help explore the content of different representations. But it is noteworthy that the particular ways that the network fails to disentangle scene variables can predict similar perceptual failures, which was not address in Ref 35.

3. The fact that in image formation confounds arise between lighting, shape and specular reflectance is already known. Where these unknowns are inherently confounded in the known image intensity values, no computational model, human or DNN is able to disentangle them. The confounding is a prior property, not one that emerges from unsupervised learning. So I disagree with the final statement of the Discussion that "unsupervised learning may account for failures of constancy" and would instead suggest that "loss of information in the image generation process" may account for failures of constancy. I might also rewrite the sentences in lines 28-32 as "Via extracting summary statistics, unsupervised deep learning achieves decomposition of image information into distinct physical causes."

We certainly agree that information is lost, and in general, failures of constancy are likely due to this loss. Indeed, this was a key motivation for the present study. However, the question is not just whether we can predict an overall decline in performance, but the specific pattern of errors that humans make.

Where failures are just due to loss of information, all models—and humans—should fail in the same way and make the same errors on an image-by-image basis. Importantly, however, we find that this is not the case.

Although both supervised and unsupervised models perform roughly equally well at the gloss estimation task, they make different predictions on an image-by-image basis. So, while both models do indeed predict failures of constancy, they predict it for *different* images. This allows us to test which model predicts humans better.

When we test the predictions of the models against humans, we find that the unsupervised model predicts the specific pattern of errors where alternative models do not.

Thus, the errors humans make cannot be attributed to an overall loss of information, but rather reveal specific aspects of the internal representations, which are well captured by the unsupervised model.

So, while we understand the motivation for the reviewer's comments, we think it fails to address some of the nuances of our results.

We have clarified in the Introduction that we are not seeking to predict the mere occurrence of failures of gloss constancy, but rather the *specific pattern* of failures (**p4, lines 26-31**), **and addressed the concern in the Discussion as follows (p17-18)**:

"One of our more intriguing results is that the unsupervised model predicted human perception better than the supervised model tested. It is important to note that this is not because humans and unsupervised networks were better at extracting ground truth in these stimuli. Categorisation-supervised networks categorised gloss almost perfectly (**Figure 3b**), and regression-supervised networks predicted continuous gloss levels almost perfectly (**Supplementary Figure 4b**), yet both predicted human judgements less well than unsupervised networks. This implies that the systematic errors exhibited by humans and the PixelVAE model are not a trivial consequence of some inherent impossibility in recovering specular reflectance for these stimuli. Nor is it explained by the supervised model reporting ground truth specular reflectance *too* faithfully. Although the supervised model is near-perfect at coarsely categorising surfaces as having high or low specular reflectance, it still predicts different *degrees* of glossiness for different images, including sometimes erroneously. Yet the supervised and unsupervised models make *different* predictions on an image-by-image basis, with the latter more closely matching those made by humans. We propose that this shared pattern of deviation from 'ideal performance' may arise from shared characteristics in how the human visual system and unsupervised model learn to encode images."

4. The failure of the DNN to predict the human inability to see gloss when highlights do not align with surface geometry (as depicted from other visual cues) is significant. This failure calls into question the meaning of the model in separating surface relief from material properties. The organisation of surface relief in the latent code is likely a result of the organisation of second-order parameters such as coverage, sharpness and contrast. As surface relief smoothly varies in a connected surface the spread of highlights will also vary in a regular way.
In this sense the DNN isn't doing (or proving that the human visual system does) something qualitatively different from what the inverse optics models (or previous analyses of glossiness perception) but only doing it quantitatively better, by putting more muscle into the predictive model.

This is an interesting an important point, and one we have discussed more fully in the revised manuscript. It is clear that the current model does not fully capture the photogeometric constraints that have been shown to be critical for human gloss perception, and we agree that it does provide some insight into what is and isn't being separated in terms of material and surface relief (shape). We believe that it is highly likely that the DNN doesn't have a representation of 3D shape, or at most a highly impoverished one. This doesn't seem entirely surprising given the diet of single, static, fronto-parallel images it was trained on. We believe that these photogeometric constraints may emerge when unsupervised DNNs are exposed to geometrically richer inputs (e.g., including binocular or motion structure), which is the focus of ongoing work. It is nevertheless interesting that no such cues are required to account for a wide range of successes and failures of constancy.

We have extended the Discussion of this point as follows (p18-19):

"One of the most notable failures of the PixelVAE in capturing human data is its insensitivity to photogeometric constraints known to affect human surface perception, such as the alignment of specular highlights with diffuse shading (7,88,89) (**Figure 8**). We believe that this failure is likely due to the relative poverty of 3D shape information in its training set. The link between highlights and diffuse shading arises from constraints imposed by the 3D shape of a surface (90,92). It seems implausible to expect any visual system trained solely on monocular, static images to develop good sensitivity to these constraints, and without a detailed representation of 3D shape, no model is likely to explain all aspects of human gloss perception (90,91,97). We tailored the training sets towards modelling variations in perceived glossiness for physically realistic surfaces, where highlights are assumed to align with surface shading. An important direction for future research is testing whether unsupervised DNNs can also learn photogeometric relationships, if training sets provide additional information about shape (e.g. through motion, stereo, occlusion, or larger variations in geometry)."

5. Another way to describe the goal of the paper is that it seeks to explain why the specular reflectance of materials alone does not predict glossiness perception perfectly. The answer isn't contained here. The DNN reproduces errors – in predicting that humans would perceive glossiness where specular reflectance is low and vice versa - but doesn't explain why it or the human makes these "misperceptions". In this sense, the DNN does not reveal anything fundamental about human vision that's not already known.

We do not fully agree with this assessment. We don't just seek to explain why in *general* there are deviations between gloss perception and specular reflectance of materials, but rather the *specific* pattern of errors that humans make across changes in other scene variables. **We identified the sentence that likely led to this impression and have removed it from the introduction (see new p4, lines 26-31)**.

We believe that the 'answer' as to why specular reflectance alone doesn't predict gloss perception is because the image structure *attributed* to the 'specular' component (by humans or the DNN) isn't just a function of specular reflectance alone. This was the point of Ref 35's paper, but that paper didn't have a *model* of when specular reflections failed to be separated from diffuse reflectance; it simply showed that the amount and strength of what *did* get segmented predicted perceived gloss (by using human observers to make those judgments).

Here, a model system has been constructed which leads to the same classification failures as humans. We agree that is not a complete answer as to why, in the sense that we have focussed at the computational level—by investigating the role of objective functions in determining perceptual judgments—rather than drilling down to the local responses of individual units within the network. However, there are also good reasons for not doing this (at least at this stage of the research program). We have created an equivalence class of 10 networks that produce similar choices when trained on the same training data with the same objective function. Yet, they differ from one another in their unit-to-unit weights. Just as human brains are physically different from one another, but still make similar perceptual judgments, this suggests that focussing too much at the level of individual units or features may not be the optimal one for seeking computational explanations of perceptual phenomena. Of course, the inner workings of the models are also interesting, but they don't inform us about the core question of interest, which is: 'how does a visual system learn to see the outside world without access to ground truth?' Our findings suggest that some kind of unsupervised learning may be responsible for the perceptual 'misjudgements', and this had not been suggested or shown previously.

Incidentally, it's also worth pointing out that alternative approaches, including single-unit recordings, fMRI or explicit process models also don't provide *complete* explanations on their own, which is why we need convergent approaches, including, we believe, what we have done here.

Moreover, our findings don't show that any old DNN trained on gloss perception will reproduce the errors that humans make. Rather, we show that different objective functions can lead to equivalent performance in categorising physical gloss, but still differ substantially in their ability to predict human judgments. **We have emphasised this point in the Discussion (p18, paragraph quoted above** in response to Point 3).

Finally, in answer to the question 'why' humans make the specific errors they make, we think we have learnt something new. The errors result at least in part because of what the visual system is optimised for. Previous theories of gloss perception have tended to take it for granted that the goal of the visual system is to estimate surface properties and that errors reflect short-cuts towards this goal, or simply reflect the inherent ambiguity of the stimuli (like the errors an ideal observer makes). Our findings suggest that at least some characteristics of human gloss perception likely result from *efficient encoding of statistical image structure*, and that gloss-related midlevel visual representations emerge as a (highly valuable) by-product of this goal. At least the specific successes and errors humans make (as distinct from an 'ideal observer') are consistent with representations that emerge from such learning processes. We are unaware of any other study showing this.

The above comments are not meant to downplay the importance of the construction of the DNN itself and the experiments in which its performance are compared with human perceptual behaviour. Neither are the following. I apologise that they are not in chronological order.

Specific comments on methodology:

6. Generalisability: Although a massive number of images were generated to train the DNN and the other networks with which it was compared, these images were nonetheless of a very restricted world: a patch of bumpy terrain, uniform in diffuse and specular reflectance across its surface, varying only in heights of bumps, and not much in cross-sectional circumference of bumps, and always viewed from directly above. Even with a large variation in camera angle and location, combined with 6 possible illuminations, the generative scenes are limited in scope. So the question arises as to how the DNN would do on images of other sorts of scenes with specular scenes? The question is whether the emergence of the material/lighting disentanglement in the DNN is a special case that results from the constrained nature of the image generation process. This question is fairly well addressed by the tests summarised in Figure 7 and supp Figure 4. But the photographs seem specially chosen to resemble the original training images (front-parallel view, limited surface relief). It would be helpful to see more analysis of where and how the network fails.

We have explored the unsupervised model's processing of real photographic images more systematically. **Supplementary Figure 6C** now shows all twenty photographs from the original test, along with their predicted gloss values. As you say, we had deliberately taken photographs that at least roughly conformed to the sorts of images the model had seen during training: close-up photographs of non-patterned surfaces with a single material, which was either clearly glossy or matte, as the limits of generalizing beyond the training set are well known. **We have also performed a more wide-ranging test, by presenting the models with the 300 images in the Giessen Material Image Database** (Wiebel, Valsecchi & Gegenfurtner, 2013), which are of diverse wood, metal, fabric, and stone surfaces. **Supplementary Figure 6D** shows the predicted gloss values for all images, along with the five images predicted to have lowest and the five predicted to have highest gloss. Although the model shows some broad successes (e.g., classing dull metallic surfaces as lower gloss and polished ones as higher gloss), it also shows clear failure cases. For example, some of the highest gloss values are assigned to matte fabrics that have high contrast stripes or patterns.

Given that the training objective of the model is to learn detailed spatial structures within a highly controlled domain of surface images, and that the linear gloss classifier used to predict gloss values from the model is also fitted on such data, it should not be surprising that gloss predictions for images far outside the training data are no longer sensible. However, its moderate success at generalising to photographs of close-ups of single-colour, single-material surfaces suggests that the gloss features it has learned do not hinge on artefacts of its artificial training domain.

7. In Experiments 1,3, and 4: are the images used in these experiments generated in the same way as for the main training set (i.e. from the original 6 HDRIs, and the original bimodal specular reflectance component distributions, and surface relief parameters)? And, therefore, for the comparison of the three PixelVAE DNNs, the images used were all from the main training set? I.e. none of the novel geometries or novel light fields were used to generate the images used in Experiments 1,3, and 4? Please clarify this in the manuscript.

Yes, this is correct. **We have clarified this on page 21, lines 28-31.**

One reason I ask this is because some of the novel geometries in Figure 7A I find very hard to read in terms of glossiness. It would be interesting to know what whether in these cases the glossiness predicted by the original PixelVAE model (or indeed one of the other two Pixel VAE models) agree with human perception of glossiness in these images.

The example images in this figure were perhaps confusing choices, since they varied not only in geometry but also in colour, glossiness, and illumination. We have replaced them with examples of the different geometries rendered with identical materials and illuminants. We hope you agree that they are more perceptually parsable now. **See updated Figure 7A**.

8. The network is trained to summarise a training set of images by "probabilistic distributions of pixel values that captured the structure and variability in and across the training images across all

spatial scales" and then to "generate plausible novel samples from that visual world". It does so with two streams: the 'conditioning' network and the 'autoregressive' stream. It's the former that encodes the images into a 10D latent code, and this is the code that's used to test the success of the network in experiments 1,3 and 4.

My question: Does the autoregressive network feed into the conditioning stream and influence the development of the 10D latent code? It appears to in Figure 1, but this is not clear from the methods, or from the main text. It is not clear whether the success of the network in predicting gloss and gloss constancy (i.e. specular reflectance or human perception) depends only on the latent code, or on the spatial probability distribution learned by the autoregressive stream. The autoregressive stream is clearly necessary for generating new images, e.g. as used in Experiment 2, but it is not clear whether it is necessary for the other tests.

The autoregressive stream does not feed into the conditioning stream (only vice versa). However, it definitely influences the development of features within the conditioning stream. The two streams are trained end-to-end in a single backpropagation sweep, with the only objective being learning a good generative pixel model at the end of the autoregressive net. The features we examine, within the 10D latent code at the end of the conditioning stream, are therefore those which have proved, over training, to provide useful information to the pixel-sampling-process in the autoregressive stream. Broadly, the PixelVAE architecture appears to divide its statistical learning by capturing whole-image properties in the latent code (e.g. surface colour and material), versus local spatial dependencies (e.g. the shape and size of our surfaces' "bumps") in the autoregressive pixel likelihood model.

In several places throughout the manuscript, it's said that the success of the network depends on its two learning objectives – data compression and spatial prediction – but I am left wondering how essential the second learning objective is to the success demonstrated in experiments 1,3 and 4. These experiments depend on gloss ratings derived from an SVM classification via the 10D latent code only.

Given the connection to the following point, we respond to both below.

9. The related question is to what do the learning objectives of the unsupervised model correspond, in human development? Since the question is posed as to why or how a supervised training would get its supervision, we should ask the equivalent question of the unsupervised model. Why or how would the unsupervised model set its objectives in this way? The unsupervised human visual system would not necessarily need to develop the ability to synthesise novel images, but it might indeed wish to develop optimal encoding strategies, given physical limitations on neural mechanisms. Therefore, the balance between optimal encoding vs. ability to generate novel images might need some restating.

Learning a good generative pixel model is likely quite important to the overall success of the PixelVAE. Our "simple autoencoder" model, which *only* learns compression, is relatively poor at predicting human gloss judgements. It is interesting to speculate whether the human visual system does in fact use an internal generative model to help it learn good representations. This is far from outlandish: many other perceptual models invoke predictive coding, and almost all models of motor learning involve an internal model that predicts the sensory consequences of actions, with errors between the model's predictions and observed action outcome driving learning. Having such a perceptual generative model may also be useful in its own right, for setting up top-down information streams for visual imagery or attentional selection. This is of course complete speculation, but we think our findings prompt new research directions to test such ideas.

Moreover, the separation between the compressive and generative aspects might not be so distinct in the human visual system as in the PixelVAE architecture. We think (speculate) that one of the main values of the generative component is to ensure representations that smoothly interpolate and extrapolate between training samples (rather than learning a very 'clumpy' representation, centred around each training item). There may be some way of achieving this locally without

explicitly plotting out pixel colours in a sequence, like the PixelVAE does. But this is an open research question. Due to the speculative nature of this discussion, we felt that it would be premature to say too much about it in the manuscript, but we are currently looking into it in follow-up projects.

10. P5 line 10: "As models of human gloss perception, it is unclear from where the necessary training labels could come." The statement implicitly refers to human development. I believe that, as stated earlier in the introduction, the idea is that a developing visual system would not be able to employ information from other senses as to the physical properties that give rise to gloss. The implication is that this problem is specific to gloss perception. But for learning of visual categories more generally, it remains a problem to determine how the supervised learning takes place in development. And the simple answer, that the supervision comes from other humans providing the training labels, would equally apply well to gloss perception.

We intended this to be a general problem statement, relevant to any organism learning to see, **and have modified the text as follows (p5, line 25)**:

"As models of biological gloss perception, it is unclear from where the necessary training labels could come."

It is worth noting that while linguistic labels could potentially play a role in certain aspects of human perceptual development, they obviously cannot account for perceptual learning in other species—which also end up with very similar perceptual representations (see, e.g., work comparing responses in human and monkey IT).

It is also worth noting that infant-perspective movie recordings of their experience during development indicate that the frequency of occurrence of word labels accompanying visual stimuli is totally swamped by the occurrence of visual stimuli without verbal labels. So even if such supervision signals play some role in perceptual learning, there must also be a significant proportion of visual learning that does not take advantage of such signals (i.e., unsupervised learning).

11. The ability of the DNN to predict human perception of glossiness in synthetic (rendered) images was compared with at least 6 other models; a hugely commendably thorough approach. Importantly, the model was compared with a simpler model based on image statistics, using the single statistical measure of luminance histogram skewness. That single measure doesn't predict human glossiness as well as the DNN does, but it seems likely that it contributes, given the previous demonstrations of its success in predicting glossiness. Therefore it would help us gain insight into what the DNN is doing if we were to see how luminance skewness paints the clusters in the latent code, similarly to the physical factors of light angle and light field, for example. Similarly, it would be interesting to see how the texture features of Ref 80 paint the clusters.

The figure below shows a 2D tSNE visualisation of the latent feature space in one unsupervised model, colour-coded by luminance histogram skewness, as suggested. The bottom left panel shows the same for one supervised model. It seems that low vs high skewness images are reasonably well separated in both model spaces, and that images are arranged relatively smoothly by their skewness in the unsupervised model. The figure below also includes the same 2D embedding for both models coloured by each of the other world factors, for comparison. Note that this is an identical analysis to that shown in Figure 2A of the main manuscript, but since the tSNE embedding algorithm is stochastic, the exact arrangement of points is slightly different each time it is run.

Evaluating the correlations with other models (e.g., the Portilla-Simoncelli model) is not so straightforward as they are multidimensional, so it is unclear what 'colour' to paint each dot. To address this we do, however, **we have now included tSNE visualisations of all alternative models in a new Supplementary Figure 2,** including texture and skewness.

Since our image set consists of a bimodal distribution of low-gloss and high-gloss images, it is to be expected that skewness correlates quite strongly with physical specular reflection. The clustering of high vs low gloss images along the skewness dimension, as well as the reasonably good accuracy of a gloss classifier based on skewness alone (Figure 6) demonstrates that skewness is indeed a correlate of gloss within our training dataset, and it is therefore necessarily represented within the unsupervised model's latent code to some degree if the model also separates images by their surface gloss.

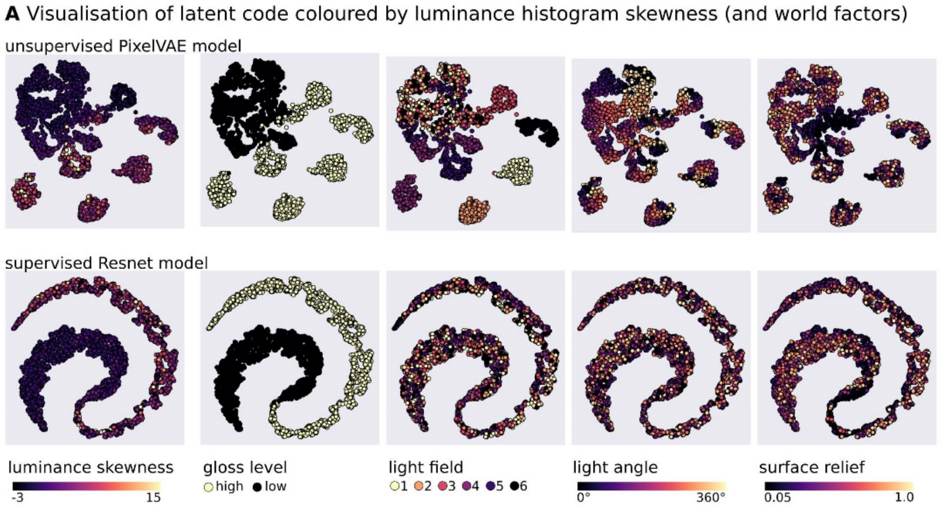

12. The Unity3D standard shader: this shader allows the diffuse (body) and specular reflections components of a surface to have different properties. It's not clear from what's written here whether the base color refers only to the diffuse component, and whether the specular reflectance component was different, and/or whether it was spectrally flat (whether it varied with wavelength). It's also not clear whether the indirect reflections between the bumps in the images were physically accurately modelled. Again, the relevance of this comment is that the model the DNN is learning might be a simplified model of the world, constrained by the limitations of the standard shader model used to generate the images.

The base colour refers only to the diffuse component. The specular component was always white, as is typical for dielectric materials. The realtime rasterised rendering we used in Unity3D did not render inter-reflections between bumps. To minimise the impact of this, we constrained maximum bump heights and maximum specular reflectance magnitudes to settings at which the appearance of inter-reflections was limited.

We have explored the possible dependence of our model on the limitations of the specific rendering methods used, by rendering a small number of surfaces with identical geometries, lighting, and base colours, once using rasterisation (the Eevee realtime rendering engine in Blender) and once using ray-tracing for physically accurate inter-reflections (the Cycles rendering engine in Blender). The unsupervised DNN model made very similar gloss value predictions for both rendering types, and correctly classified the gloss level (high vs low) of all surfaces, regardless of rendering method (**see new Supplementary Figure 6B**).

Minor comments:

P 18: The last 2 paragraphs (from line 5) describe what must be experiment 4, but it isn't labelled as such.

Thank you, we have fixed this.

P 18:, line 19-21. ".. the arrangement of stimuli in the unsupervised model's latent space…" This sentence needs some unpacking for me. Is the implication that distances between stimuli, as measured in the multi-dimensional latent variable space of the DNN, correspond to differences in the estimated gloss of pairs of images? If that's what meant, then this doesn't, I think, follow directly from the data. The human data shows almost no variation in "deviation from constancy" for the positive deviations – i.e. a flat horizontal for positive values in Figure 5E – whereas the model predicts values with much more variation (from 0 to 10). The human data make the decision look categorical, whereas the model makes it look continuous. Thus, the organisation of the DNN's perceptual space appears qualitatively different from the human perceptual space. I might have misunderstood the sentence. In any case, it needs to be made clearer what's mean, and then justified better.

We believe the way the conclusion was presented is supported by the data, as the statistical tests of this claim are statistically significant. We agree that there is not a linear mapping between distance in the DNN's latent space and difference in perceived gloss, but we already state this, as fitting a logistic function increases the proportion of variance explained. However, to avoid confusion **we have simply removed the sentence from the manuscript (see p12, line 29 – p13, line 4)**.

Figure 7 caption: (A) x-axis and y-axis are cited the wrong way round. (B) "were were"

Thank you, both now fixed.

Decision Letter, first revision:

20th January 2021

*Please ensure you delete the link to your author homepage in this e-mail if you wish to forward it to your co-authors.

Dear Dr Storrs,

Thank you once again for submitting your revised manuscript, entitled "Unsupervised Learning Predicts Human Perception and Misperception of Gloss," and for your patience during the re-review process.

Your manuscript has now been evaluated by our referees, and in the light of their advice I am delighted to say that we can in principle offer to publish it. First, however, we would like you to revise your paper to address the points made by the reviewers, and to ensure that it complies with our Guide to Authors at http://www.nature.com/nathumbehav/info/gta.

Reviewer #3 mentions a number of interesting discussion points. While we fully agree that incorporating comments on these issues would broaden the scope of the discussion for expert readers, we will leave it at your discretion to decide in how far the discussion would benefit from each individual point, keeping both expert readers and our broader audience in mind.

One of the main reasons for delays in formal acceptance is failure to fully comply with editorial policies and formatting requirements. To assist you with finalizing your manuscript for publication, I attach a checklist that lists all of our editorial policies and formatting requirements. I also attach a template document, which exemplifies our policies and formatting requirements.

Please attend to *every item* in the checklist and upload a copy of the completed checklist with your submission. I have highlighted in the checklist items that require your attention. I also mention here a few points that are frequently missed and can cause delays:

1) Ensure that all corresponding authors have linked their ORCID to their account on our online manuscript handling system. This is very frequently missed and invariably causes delays in formal acceptance.

2) Ensure that you provide all of the materials requested in the attached checklist and below with your final submission.

3) Pay close attention to our guidelines for statistics reporting and interpretation. I have included a few examples for where your manuscript does not comply with our guidelines on the attaches checklist.

Nature Human Behaviour offers a transparent peer review option for new original research manuscripts submitted from 1st December 2019. We encourage increased transparency in peer review by publishing the reviewer comments, author rebuttal letters and editorial decision letters if the

authors agree. Such peer review material is made available as a supplementary peer review file. Please state in the cover letter ‘I wish to participate in transparent peer review’ if you want to opt in, or ‘I do not wish to participate in transparent peer review’ if you don’t. Failure to state your preference will result in delays in accepting your manuscript for publication.
Please note: we allow redactions to authors’ rebuttal and reviewer comments in the interest of confidentiality. If you are concerned about the release of confidential data, please let us know specifically what information you would like to have removed. Please note that we cannot incorporate redactions for any other reasons. Reviewer names will be published in the peer review files if the reviewer signed the comments to authors, or if reviewers explicitly agree to release their name. For more information, please refer to our FAQ page.

We hope to hear from you within 10 days; please let us know if the revision process is likely to take longer.

To submit your revised manuscript, you will need to provide the following:
• Cover letter
• Point-by-point response to the reviewers (if applicable)
• Manuscript text (not including the figures) in .docx or .tex format
• Individual figure files (one figure per file)
• Extended Data & Supplementary Information, as instructed
• Reporting summary
• Editorial policy checklist
• Third-party rights table (if applicable)
• Suggestions for cover illustrations (if desired)

Consortia authorship:
For papers containing one or more consortia, all members of the consortium who contributed to the paper must be listed in the paper (i.e., print/online PDF). If necessary, individual authors can be listed in both the main author list and as a member of a consortium listed at the end of the paper. When submitting your revised manuscript via the online submission system, the consortium name should be entered as an author, together with the contact details of a nominated consortium representative. See https://www.nature.com/authors/policies/authorship.html for our authorship policy and https://www.nature.com/documents/nr-consortia-formatting.pdf for further consortia formatting guidelines, which should be adhered to prior to acceptance.

Reviewer Recognition:
In recognition of the time and expertise our reviewers provide to Nature Human Behaviour’s editorial process, we would like to formally acknowledge their contribution to the external peer review of your manuscript entitled "Unsupervised Learning Predicts Human Perception and Misperception of Gloss". For those reviewers who give their assent, we will be publishing their names alongside the published article.

Forms:
Nature Human Behaviour has now transitioned to a unified Rights Collection system which will allow our Author Services team to quickly and easily collect the rights and permissions required to publish your work. Once your paper is accepted, you will receive an email in approximately 10 business days providing you with a link to complete the grant of rights. If you choose to publish Open Access, our

Author Services team will also be in touch at that time regarding any additional information that may be required to arrange payment for your article.

Please note that you will not receive your proofs until the publishing agreement has been received through our system.
If you have any questions please contact ASJournals@springernature.com.

Please use the following link for uploading these materials:
[REDACTED]

If you have any further questions, please feel free to contact me.

With best regards,

Marike Schiffer

Marike Schiffer, PhD
Senior Editor
Nature Human Behaviour

Reviewer #1:
Remarks to the Author:
The authors have addressed all my comments, and made substantial changes to the manuscript that makes it a strong submission. For that reason, I recommend its acceptance.

Reviewer #2:
Remarks to the Author:
Thanks for addressing my concerns! I think this paper is ready for publication.

Reviewer #3:
Remarks to the Author:
I thank the authors very much for taking such care to consider and address the first review. The response and the revised paper are both a pleasure to read. I enjoy the engagement with the important issues of motivation and interpretation, and indeed philosophical viewpoint. The computational work (and I like its description as such, as distinct from algorithmic work) is very strong, particularly since, as the authors point out so well in their response, yes, there is no good analytic inverse optics-type model around that successfully predicts human gloss judgments. I've got only two minor changes to suggest (in points 7 and 12 below). Otherwise, just comments.
1-2. Re the responses to points 1 and 2, and the corresponding edits in the paper: I am totally happy with these.
It is a very engaging discussion and one very much worth having, at the heart of understanding vision, or at least at the heart of understanding models of vision. Of course I do not disagree that the

core question is how the visual system can infer physical properties from the proximal image, and of course I recognise that the visual system is not taught a priori what classes and instances of physical distal properties exist before it deduces the mapping between these and proximal image variations.
I agree that the general notion of an “inverse optics” model is too general to help with distinguishing between competing models of vision. So I entirely applaud the removal of the claim from the earlier version of the manuscript which pitted this specific DNN against inverse optics models.
In my comparison between the mappings of an inverse optics model – an analytic model that relates proximal image variations to distal physical properties – and the mapping learned by the DNN, the equivalence I drew was between the mappings once they exist, not between how they are derived. The DDN learns the model in an unsupervised way, driven not by the goal to calculate the most likely set of predefined physical parameters (e.g. in a Bayesian formulation), but instead to find the most efficient code to represent all encountered variations in image pixels.
3. I like this clarification on predictions of failures of constancy. It’s very helpful.
4. I agree with the conclusion that the network hasn’t learned to represent 3D shape well most likely due to the lack of 3D shape variation in the training stimuli. My point here was that in the latent code the model still looks like it’s distinguishing between surface relief and material properties, but in fact it might not be. It will be interesting to see how the latent code alters when the network is trained on a richer set of shape stimuli. It would also be interesting to know whether a naïve human visual system that saw only such impoverished stimuli would behave like this network when confronted with unaligned highlights, the argument being here that the reason the network fails to predict this particular feature of human gloss perception lies in the difference of visual diet. Not an easy experiment to do in humans, but maybe with monkeys?
5. This response is all very well-argued – thank you. I am just wondering – and this isn’t something you need to answer – whether there is any proof that this DNN produces the *most* efficient encoding of statistical image structure possible. I.e. does another statistical method exist which improves on the DNN?
6. So the network doesn’t generalise well beyond its training set. That’s not a deal breaker for me, in the context of the computational point you are trying to make.
7. Thanks, I do find these easier to parse perceptually. Would you add into the caption the specular reflectance parameter for the particular sample novel images shown in Figure 7A?

8-9. This is a *really* interesting avenue to explore. Excited to hear about further progress you make here.
12. For clarity and technical accuracy, I think you should explicit refer to the diffuse reflectance component RGB values, not “base colour” or “colour” as in used in the description of the images on p 6 (Results).
The generalisation to outputs of more physically accurate models is nice to see.

Author Rebuttal, first revision:

Editorial checklist comments:

Please always report statistics in full, as listed here; this applies for example to reports of t-tests, main effects of ANOVAs and correlations.

All statistical tests are now accompanied by effect sizes and confidence intervals.

For your model comparison., can you specify whether the models had the same number of free parameters? If this was not the case, could you explain how your model comparison corrects for that (you use RSME scores)?

All models have zero free parameters, at the time of evaluation against human data. Although the deep neural network models of course have a very large number of trainable parameters, these are frozen after training, and training does not involve any exposure to human perceptual data. The gloss predictions of all models are treated as fixed. We have included a statement to this effect in a new "Statistical Analysis" section of the Methods (**see p28, lines 23-25**).

Statements such as the following need to be revised:
"However, in the supervised models, only clustering by gloss was apparent (Figure 2E; gloss t9 = 112.2, p = 3x10-28; lighting t9 = 1.77, p = 0.08)"

This sentence suggests the clustering was specific to gloss, but since the statistics offered for lightning do not afford the interpretation of no effect, specificity has not been demonstrated.

To argue for a difference in the strength of effect, an interaction contrast would be a suitable solution; to demonstrate specificity, you would need to employ statistics that offer positive evidence for no effect, i.e., Bayesian statistics or equivalence tests.

We have provided a test of the interaction, and reworded the claim accordingly. **See p7, lines 17-31**.

You note that your experiments were conducted in accordance with the Declaration of Helsinki, but you don't specify which issue. Note that to be compliant with the last version, the experiments would need to have been preregistered (amongst all other requirements)

Thank you for identifying this. We have specified that human experiments were in accordance with the previous, 6th Edition of the Declaration. **See Methods, p18, line 12**.

Please include a separate Data Availability Statement and include a link to your data deposition (Thank you for making it publically available).

Please include a separate Code Availability Statement and include a link to your code deposition (Thank you for making it publically available).

We have now deposited human data, model predictions, and analysis code at the **Zenodo repository: http://doi.org/10.5281/zenodo.4495586**, and provided separate data and code availability statements in the Methods, **see p28, lines 27-32**.

Please note that the only item we were not able to respond positively to in the Checklist was the confirmation that the main text is less than 5,000 words. Currently it is 5,673 words. While preparing the previous revision, we asked for permission to exceed the word limit since reviewers asked for extensive additional analyses, which was given by Dr Schiffer (email dated 19th November 2020).

Reviewer #3:

I thank the authors very much for taking such care to consider and address the first review. The response and the revised paper are both a pleasure to read. I enjoy the engagement with the important issues of motivation and interpretation, and indeed philosophical viewpoint. The computational work (and I like its description as such, as distinct from algorithmic work) is very strong, particularly since, as the authors point out so well in their response, yes, there is no good analytic inverse optics-type model around that successfully predicts human gloss judgments.

We thank the reviewer for their engaging and thought-provoking reviews!

I've got only two minor changes to suggest (in points 7 and 12 below). Otherwise, just comments.
1-2. Re the responses to points 1 and 2, and the corresponding edits in the paper: I am totally happy with these.

It is a very engaging discussion and one very much worth having, at the heart of understanding vision, or at least at the heart of understanding models of vision. Of course I do not disagree that the core question is how the visual system can infer physical properties from the proximal image, and of course I recognise that the visual system is not taught a priori what classes and instances of physical distal properties exist before it deduces the mapping between these and proximal image variations.

I agree that the general notion of an "inverse optics" model is too general to help with distinguishing between competing models of vision. So I entirely applaud the removal of the claim from the earlier version of the manuscript which pitted this specific DNN against inverse optics models.

In my comparison between the mappings of an inverse optics model – an analytic model that relates proximal image variations to distal physical properties – and the mapping learned by the DNN, the equivalence I drew was between the mappings once they exist, not between how they are derived. The DDN learns the model in an unsupervised way, driven not by the goal to calculate the most likely set of predefined physical parameters (e.g. in a Bayesian formulation), but instead to find the most efficient code to represent all encountered variations in image pixels.

3. I like this clarification on predictions of failures of constancy. It's very helpful.

4. I agree with the conclusion that the network hasn't learned to represent 3D shape well most likely due to the lack of 3D shape variation in the training stimuli. My point here was that in the latent code the model still looks like it's distinguishing between surface relief and material properties, but in fact it might not be. It will be interesting to see how the latent code alters when the network is trained on a richer set of shape stimuli. It would also be interesting to know whether a naïve human visual system that saw only such impoverished stimuli would behave like this network when confronted with unaligned highlights, the argument being here that the reason the network fails to predict this particular feature of human gloss perception lies in the difference of visual diet. Not an easy experiment to do in humans, but maybe with monkeys?

5. This response is all very well-argued – thank you. I am just wondering – and this isn’t something you need to answer – whether there is any proof that this DNN produces the *most* efficient encoding of statistical image structure possible. I.e. does another statistical method exist which improves on the DNN?

We don’t believe the PixelVAE architecture and learning objective should necessarily arrive at the *most* efficient encoding of statistical structure. For example, the structures learnable by any given PixelVAE network will be constrained by architectural details of that network, such as the size and scaling of receptive fields across its layers. Developments in machine learning theory may improve our understanding of the relative optimality of different unsupervised learning objectives, over the coming years.

6. So the network doesn’t generalise well beyond its training set. That’s not a deal breaker for me, in the context of the computational point you are trying to make.

7. Thanks, I do find these easier to parse perceptually. Would you add into the caption the specular reflectance parameter for the particular sample novel images shown in Figure 7A?

We have added this information to the **Figure 7 caption**.

8-9. This is a *really* interesting avenue to explore. Excited to hear about further progress you make here.

12. For clarity and technical accuracy, I think you should explicit refer to the diffuse reflectance component RGB values, not “base colour” or “colour” as in used in the description of the images on p 6 (Results).

We have adopted this more precise terminology, **see Methods p18 line 30 – p19 line 1**.

The generalisation to outputs of more physically accurate models is nice to see.

Final Decision Letter:

Dear Dr Storrs,

We are pleased to inform you that your Article "Unsupervised Learning Predicts Human Perception and Misperception of Gloss", has now been accepted for publication in Nature Human Behaviour.

Before your manuscript is typeset, we will edit the text to ensure it is intelligible to our wide readership and conforms to house style. We look particularly carefully at the titles of all papers to ensure that they are relatively brief and understandable.

Once your manuscript is typeset and you have completed the appropriate grant of rights, you will receive a link to your electronic proof via email with a request to make any corrections within 48 hours. If, when you receive your proof, you cannot meet this deadline, please inform us at rjsproduction@springernature.com immediately. Once your paper has been scheduled for online

publication, the Nature press office will be in touch to confirm the details.

Acceptance of your manuscript is conditional on all authors' agreement with our publication policies (see http://www.nature.com/nathumbehav/info/gta). In particular your manuscript must not be published elsewhere and there must be no announcement of the work to any media outlet until the publication date (the day on which it is uploaded onto our web site).

Nature Human Behaviour is a Transformative journal and offers an immediate open access option through payment of an article-processing charge (APC) for papers submitted after 1 January, 2021. In the event that authors choose to publish under the subscription model, Nature Research allows authors to self-archive the accepted manuscript (the version post-peer review, but prior to copy-editing and typesetting) on their own personal website and/or in an institutional or funder repository where it can be made publicly accessible 6 months after first publication, in accordance with our self-archiving policy. Please review our self-archviing policy for more information.

Several funders require deposition the accepted manuscript (AM) to PubMed Central or Europe PubMed Central. To enable compliance with these requirements, Nature Research therefore offers a free manuscript deposition service for original research papers supported by a number of PMC/EPMC participating funders. If you do not choose to publish immediate open access, we can deposit the accepted manuscript in PMC/Europe PMC on your behalf, if you authorise us to do so.

If you have posted a preprint on any preprint server, please ensure that the preprint details are updated with a publication reference, including the DOI and a URL to the published version of the article on the journal website.

An online order form for reprints of your paper is available at https://www.nature.com/reprints/author-reprints.html. All co-authors, authors' institutions and authors' funding agencies can order reprints using the form appropriate to their geographical region.

We welcome the submission of potential cover material (including a short caption of around 40 words) related to your manuscript; suggestions should be sent to Nature Human Behaviour as electronic files (the image should be 300 dpi at 210 x 297 mm in either TIFF or JPEG format). Please note that such pictures should be selected more for their aesthetic appeal than for their scientific content, and that colour images work better than black and white or grayscale images. Please do not try to design a cover with the Nature Human Behaviour logo etc., and please do not submit composites of images related to your work. I am sure you will understand that we cannot make any promise as to whether any of your suggestions might be selected for the cover of the journal.

You can now use a single sign-on for all your accounts, view the status of all your manuscript submissions and reviews, access usage statistics for your published articles and download a record of your refereeing activity for the Nature journals.

To assist our authors in disseminating their research to the broader community, our SharedIt initiative provides you with a unique shareable link that will allow anyone (with or without a subscription) to

read the published article. Recipients of the link with a subscription will also be able to download and print the PDF.

As soon as your article is published, you will receive an automated email with your shareable link.

In approximately 10 business days you will receive an email with a link to choose the appropriate publishing options for your paper and our Author Services team will be in touch regarding any additional information that may be required.

You will not receive your proofs until the publishing agreement has been received through our system.

If you have any questions about our publishing options, costs, Open Access requirements, or our legal forms, please contact ASJournals@springernature.com

We look forward to publishing your paper.

With best regards,

Marike Schiffer

Marike Schiffer, PhD
Senior Editor
Nature Human Behaviour

P.S. Click on the following link if you would like to recommend Nature Human Behaviour to your librarian http://www.nature.com/subscriptions/recommend.html#forms

** Visit the Springer Nature Editorial and Publishing website at www.springernature.com/editorial-and-publishing-jobs for more information about our career opportunities. If you have any questions please click here.**
